# A Theory-Driven Game-Based Assessment of Collaborative Problem-Solving

**DOI:** 10.3390/jintelligence14090197

**Published:** 2026-09-01

**Authors:** Shaoyang Guo, Yinmei Xu, Xiangdong Yang, Yanlei Chen, Xiaoyu Li, Xuefei Tian

**Affiliations:** 1 School of Education and Intelligent Education Research Center, Yangzhou University, Yangzhou 225009, China; syguo1992@yzu.edu.cn (S.G.); xuyinmei129@outlook.com (Y.X.); lixiaoyu0228@outlook.com (X.L.); 008644@yzu.edu.cn (X.T.); 2 Department of Educational Psychology, East China Normal University, Shanghai 200062, China; 3 School of Educational Science, Liaocheng University, Liaocheng 252059, China; chenyanlei@lcu.edu.cn

**Keywords:** collaborative problem-solving, theory-driven assessment, games, measurement

## Abstract

Collaborative problem-solving (CPS) is an essential 21st-century competency, yet its valid and ecologically sound assessment remains challenging due to inconsistent frameworks, poorly aligned task design, and over-reliance on behavior frequency counting in scoring. This study developed and provided empirical support for a theory-driven game-based CPS assessment embedded in a real-time strategy game, using a customized Collaborative Siege Task with asymmetric roles and ill-structured problems, to elicit more realistic collaborative interactions. Based on a validated three-facet CPS framework from Chinese cultural context, the task systematically triggers social and cognitive processes of collaboration across five progressive stages. By sampling from the eastern provinces of China, a pilot study with 36 participants confirmed task feasibility and indicator effectiveness, while a formal study with 298 valid participants examined psychometric properties using item response theory and confirmatory factor analysis. Findings supported the hierarchical higher-order factor structure, satisfactory item difficulty, discrimination, reliability, and criterion-related validity against peer assessment, reasoning ability, and personality. The assessment showed no sex, major, or role bias. This study provides a reliable gamified assessment tool for CPS measurement with demonstrated potential for scalability pending further development of automated scoring procedures, offering implications for large-scale evaluation and competency-oriented instruction.

## 1. Introduction

The dawn of the intelligent era has brought unprecedented transformations to the structure of modern work and education, characterized by increasingly refined division of labor and the critical need for real-time, interconnected collaboration among individuals across diverse contexts (Andrews-Todd & Forsyth, 2020). In this landscape, collaborative problem solving (CPS) has emerged as a cornerstone 21st-century skill, recognized as indispensable for student success in academic, professional, and personal domains by intergovernmental bodies and leading educational organizations worldwide (Griffin et al., 2012). The Organization for Economic Co-operation and Development (OECD), through its Programme for International Student Assessment (PISA), has defined CPS as a domain-independent competency that enables individuals to effectively engage in team-based problem solving by pooling shared knowledge, skills, and efforts through communication and collective understanding (OECD, 2017). Similarly, the Assessment and Teaching of 21st Century Skills (ATC21S) project and the Partnership for 21st Century Learning have prioritized CPS as a high-priority competency to be integrated into formal education, from K-12 to higher education (Fadel & Trilling, 2009; Griffin & Care, 2015).

As a core component of students’ comprehensive development, the cultivation of CPS competency holds multifaceted significance. CPS not only fosters deep learning through collaborative knowledge construction but also shapes students’ open and inclusive mindsets, enhances their interpersonal skills, and reduces risks of anxiety and depression by building robust social support systems (Graesser et al., 2020; Guo & Yang, 2023). Moreover, CPS has become a critical criterion in corporate recruitment, with proficient collaborative problem solvers gaining distinct competitive advantages in the global job market (O’Neill et al., 2012). To effectively nurture this competency in education, it is imperative to establish a rigorous, operationalizable framework for CPS that can guide its assessment and instruction (von Davier et al., 2017).

CPS is fundamentally conceptualized as a coordinated, joint activity where two or more individuals combine their expertise and efforts to transform a problem state into a desired goal state (OECD, 2013; Roschelle & Teasley, 1995). At its core, CPS is an integrated construct that inextricably links cognitive skills for problem structuring, solution planning, and execution monitoring with social skills for perspective-taking, negotiation, and team dynamics maintenance (Care et al., 2016). Unlike decontextualized individual problem-solving or generic teamwork skills, CPS is fully expressed only through interpersonal interactions, requiring team members to establish shared understanding of the problem, align on solution strategies, regulate collective progress, and navigate disagreements while upholding positive team functioning. This integrated nature of CPS has made game-based assessment an increasingly promising approach for its measurement (Sun et al., 2025). The gamified environments can elicit more realistic CPS behaviors with increased ecological validity relative to traditional assessments in low-stakes, engaging contexts, reduce test anxiety through stealth assessment, and capture fine-grained process data of collaborative interactions (Rahimi et al., 2026; Stoeffler et al., 2020; Sun et al., 2020).

Although the educational value of CPS and the potential of game-based assessment have been widely acknowledged, three fundamental challenges remain unresolved in this field, which will be elaborated in the Section 2. First, existing CPS assessment frameworks lack general consensus and empirical support (Care et al., 2016; Sun et al., 2020). Second, assessment tasks fail to closely align with theoretical frameworks, especially for the indicator-level (Rosen, 2015; Wu et al., 2019). Third, scoring approaches prioritize quantity over substantive quality (Stoeffler et al., 2020; Yuan et al., 2019). These obstacles hinder the effective teaching, evaluation and cultivation of CPS competence, and they have also driven this study to explore a theory-driven game-based assessment approach that integrates theoretical guidance into every step of instrument development and validation.

Specifically, this study grounds the conceptualization and operationalization of CPS in a Delphi-validated framework tailored to the Chinese cultural context (Guo & Yang, 2025). This framework follows a hierarchical structure encompassing an overarching competency, three facets, six sub-facets, and 17 observable elements, each with three-level performance descriptors that connect abstract competencies to concrete behaviors. The game-based task is systematically aligned with this framework through cognitive task analysis, using ill-structured problems and asymmetric interdependent roles to elicit authentic collaboration. A three-dimensional trigger system activates the required social and cognitive processes, and five progressive stages cover the complete CPS process. For scoring, this study adopts a quality-focused approach that captures the substantive meaning and functional value of collaborative interactions instead of mere behavioral frequency. Empirical testing using item response theory (IRT) and confirmatory factor analysis (CFA) supports the factor structure and psychometric properties, closing the theory-evidence loop.

By unifying framework design, task development, evidence extraction, and scoring within a single coherent system, this study addresses the misalignment between theory and measurement, enhances task validity, and improves scoring interpretability. Unlike previous game-based CPS assessments that often focus on the technological implementation of assessment environments, the primary contribution of this work lies in establishing a complete theory-to-measurement pipeline. This pipeline proceeds from a validated competency framework, through systematically aligned task design and indicator definition, to empirical psychometric validation. This coherent integration, rather than the game itself, constitutes the core scientific contribution of the study. To further test whether the theory-driven game-based approach proposed by this study can effectively improve the CPS assessment, four interrelated research questions will be investigated: (1) How to design the gamified assessment tasks based on the CPS assessment framework? (2) How to extract behavioral indicators for CPS competency assessment based on gaming processes and conduct a qualitative evaluation? (3) To what degree does the factor structure derived from empirical data collected in game-based CPS tasks align with the theoretical structure of the assessment framework? (4) What is the item quality, reliability, and validity in the gamified CPS assessment tasks?

The following article is organized as follows: First, a literature review is conducted to identify existing gaps in current CPS assessment and directions for improvement. Second, the design of gamified tasks will be discussed. Then, a small-scale pilot study for cognitive task analysis is performed to verify the feasibility of gamified task design and feature extraction methods. Subsequently, research involving 298 participants was carried out to examine the item quality, reliability, and validity of the game-based assessment. Finally, the main findings are summarized and discussed, and directions for future research are proposed.

## 2. Literature Review

The dawn of the intelligent era has brought unprecedented transformations to the structure of modern work and education, characterized by increasingly refined division of labor. 

### 2.1. The Assessment Framework of CPS

CPS is an integrated competency that combines cognitive problem-solving processes and social interactive skills (Care et al., 2016; OECD, 2017). A clear, operational framework is essential for valid assessment. Two authoritative models have shaped global research: the ATC21S framework and the PISA 2015 framework. The ATC21S framework divides CPS into separate social and cognitive dimensions (Griffin et al., 2012). Although it partially supports behavioral coding, its separate dual-structure fails to reflect the integrated nature of real-world collaboration (Guo & Yang, 2023). The PISA 2015 framework adopts a process-oriented and integrated design (OECD, 2017), yet it was developed for 15-year-old students in human–agent settings, limiting its applicability to unstructured, adult human–human collaboration (Hesse et al., 2015; Yuan & Liu, 2016). To address these limitations, Sun et al. (2020) developed a generalized CPS model integrating cognitive and social dimensions into three validated core facets (constructing shared knowledge, negotiation/coordination, and maintaining team function), overcoming the shortcomings of prior frameworks.

Building on this, it can be seen from Table 1 and Supplementary Materials A that Guo and Yang (2025) further expanded Sun et al. (2020) and Griffin et al. (2012)’s framework for Chinese students, and established explicit mappings between observable behavioral indicators and CPS competency levels, providing a robust theoretical foundation for empirical research and game-based CPS assessment. This framework consists of three facets, namely Reaching Team Consensus (RTC), Organizing Team Actions (OTA), and Maintaining Team Function (MTF), and these three facets are conceptually interdependent rather than independent. RTC serves as the foundation for establishing shared mental models, without which coordinated action cannot proceed. OTA translates such shared understanding into goal-directed execution through planning and real-time adjustment. MTF sustains the collaborative process by monitoring team dynamics and providing socio-emotional support. This integrated structure aligns with the conceptualization of CPS advanced by Care et al. (2016) and OECD (2017). Specifically, effective RTC reduces ambiguity in OTA, successful OTA builds team confidence that strengthens MTF, and strong MTF encourages open communication and constructive risk-taking.

Nevertheless, the research conducted by Guo and Yang (2025) was mainly based on theoretical reasoning and the expert Delphi method; their research conclusions were not supported by empirical data. Therefore, it is necessary to empirically examine whether the factor structure extracted from actual game-based CPS task data is consistent with this theoretically constructed framework, so as to verify its validity and applicability in real-world CPS assessment.

### 2.2. The Task Design of CPS

Task design translates theoretical frameworks into observable behaviors and determines the ecological validity of CPS assessment (Mislevy et al., 2012). Early tasks were often well-structured with lower task difficulty, which simplified administration but failed to elicit complex collaborative processes (Griffin et al., 2012; Yuan, 2017). Gamified and simulation-based tasks have improved engagement and authenticity (Stoeffler et al., 2020; Sun et al., 2020), yet misalignment between tasks and frameworks remains a critical issue.

The ideal CPS tasks should align with theoretical frameworks, target specific cognitive and social processes, and build ecologically valid scenarios for participant interdependence. However, existing regular and gamified CPS tasks typically use only abstract or high-level concepts to guide design (Heinzen et al., 2015; Nicholson, 2015). Specifically, developers focus on whether the task elicits collaboration in general rather than whether it systematically targets the specific cognitive and social processes outlined in the CPS framework (Shute et al., 2016; Stoeffler et al., 2020; Yuan et al., 2019). Even studies that adopt established frameworks such as ATC21S often fail to analyze how task components map onto indicator-level behaviors and cognitive processes. Instead, they use general criteria of the facet level, including participation, generating solutions, and commenting on actions to judge task validity (Care et al., 2018). This gap leads to incomplete indicator elicitation, reactive observation, and poor replicability. To overcome these problems, tasks must be designed to proactively trigger framework-specified behaviors through structured scenarios, asymmetric roles, and ill-structured problems.

### 2.3. The Scoring Approaches of CPS

Scoring converts behavioral data into valid evidence of CPS proficiency, and the traditional methods rely heavily on frequency counting of predefined behaviors or keywords (Yuan & Liu, 2016). For instance, the theoretical framework of ATC21S describes high-quality interaction as initiative, responsiveness, and facilitation rather than simple quantity; but in practice, assessments intend to measure how students interact but end up measuring how often they interact (Griffin et al., 2012). While efficient, this approach overvalues high-frequency low-value behaviors and underrepresents rare but critical interactions such as perspective-taking and strategic coordination (Guo & Yang, 2023). Consequently, scores reflect behavioral quantity rather than collaborative quality, distorting construct interpretation.

Empirical data from Sun et al. (2020) further show that behavioral indicators differ sharply in observable frequency; some actions, such as proposing solutions, appear dozens of times per session, while others, such as responding to teammates or interrupting, occur rarely. When these highly skewed frequencies are directly used as observed scores or converted into ordinal data, the covariation among indicators is artificially shaped by baseline observability instead of the underlying CPS facets. As a result, the explanatory power of statistical models is greatly weakened and fails to accurately differentiate between varying levels of CPS proficiency. Therefore, it is critical to explore how to meaningfully extract behavioral indicators from gaming processes for qualitative CPS evaluation and to verify the item quality, reliability, and validity of the resulting gamified assessment to ensure accurate and interpretable measurement of CPS competence.

## 3. Task Design of Collaborative Siege Task in a Real-Time Strategy Game

### 3.1. Middle-Level Design: Overview

**Figure 1:**
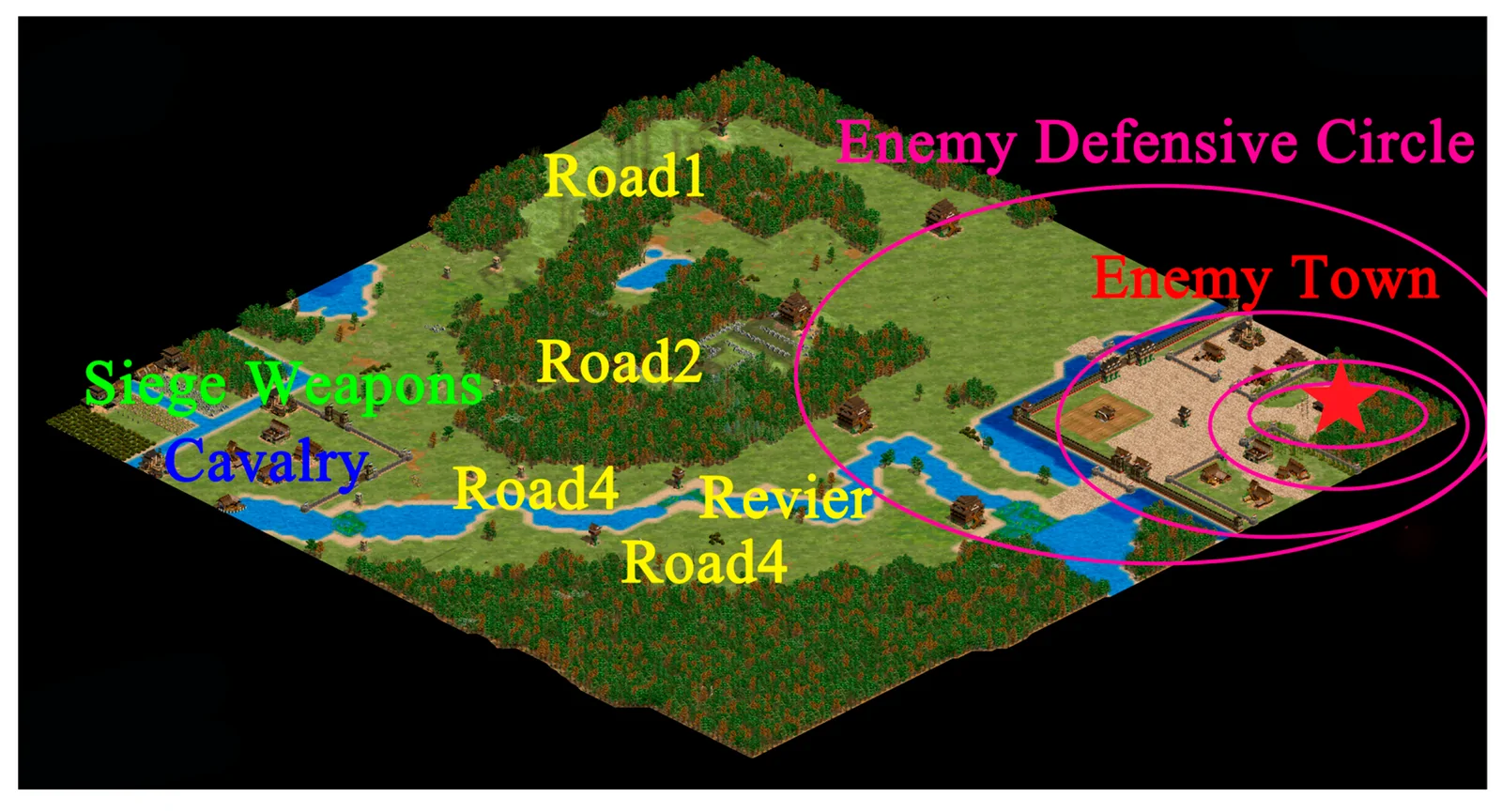
The game maps for the collaborative siege task.

This study developed a Collaborative Siege Task embedded in a real-time strategy (RTS) game environment, set in an ancient warfare context and specifically designed for dyadic CPS. This task is concretely scenario-based, tightly anchored to Guo and Yang (2025)’s CPS competency framework, and designed to elicit observable, framework-aligned CPS behaviors through a progressive, ill-structured dyadic task. Figure 1 presents a top-down map of the Collaborative Siege Task. As illustrated, the task involves three forces. Two participants form an allied force, assuming the roles of Cavalry Commander (blue) and Siege Weapon Commander (green), respectively, positioned on the left side of the map. The enemy force (red), stationed on the right side, is controlled by pre-programmed artificial intelligence and engages only in passive defense.

At the start of the task, each member of the allied collaboration team owns an independent base for troop training and logistical supply. The enemy occupies a heavily fortified stronghold surrounded by a moat (marked by the pink defensive perimeter). The core task for the two collaborators is to communicate, engage in coordinated combat, and jointly destroy the enemy’s Town Center (marked by the red five-pointed star) as quickly as possible. Between the alliance and the enemy lies a large forest and a river. The alliance may launch offensives via four land routes through the forest and one river route. Meanwhile, the enemy has deployed various defensive structures along these routes for perimeter defense. Following the principle of task completion sequence and progressive difficulty, the task is divided into five subtasks.

**Task 1. Resource Preparation and Unit Training Task:** Watch and read the video and paper-based instructions, enter the game interface, recognize, collect, and utilize resources within the allied base to train respective troops.
**Task 2. Exploration and Path Selection Task:** Lacking battlefield intelligence, the alliance is unaware of the enemy stronghold’s location. They need to dispatch units to explore the map, determine the advance route, break through the outermost defensive perimeter of the enemies, and clear pathways for the subsequent siege phase.
**Task 3. Outer Gate Breaching Task:** Due to the moat, only two city gates are accessible for attack. The alliance needs to probe the firepower of both gates, select one to launch a concentrated assault, and break through the second defensive perimeter.
**Task 4. Turret Destruction Task:** After breaking through the city gates, the enemies have built powerful turrets inside the inner city. The alliance must collaborate to destroy these turrets and breach the third defensive perimeter.
**Task 5. Town Center Capture Task:** Following the destruction of the turrets, the remaining enemy main forces will hold firm at the fourth defensive perimeter in front of their headquarters. The alliance achieves victory by eliminating the enemy troops and destroying the Town Center.

### 3.2. Underlying-Level Design: Mechanism

All tasks were designed to activate the three facets through distinct mechanisms. RTC is triggered by information asymmetry and role interdependence, compelling participants to exchange information and negotiate strategies. OTA is elicited by ill-structured problems that require goal setting, problem decomposition, and real-time execution. MTF is activated by high-difficulty, high-stakes contexts that create opportunities for collective awareness and socio-emotional support. This three-pronged design ensures systematic elicitation of framework-specified behaviors. To intuitively present the task logic and CPS measurement mechanism, the core segment of the whole task, namely the Turret Destruction Task, is adopted as a concrete example for detailed elaboration. The detailed design of all five tasks is provided in Supplementary Materials B. The Turret Destruction Task is a typical ill-structured collaborative problem-solving link, which can fully trigger the integrated behaviors of cognitive and social dimensions of CPS, and is the key segment to distinguish the level of participants’ CPS competency. After breaking through the outer city defense, the participants enter the inner city and find that the only path to the enemy’s town center is blocked by a high-attack turret, which cannot be bypassed and must be destroyed cooperatively.

**Figure 2:**
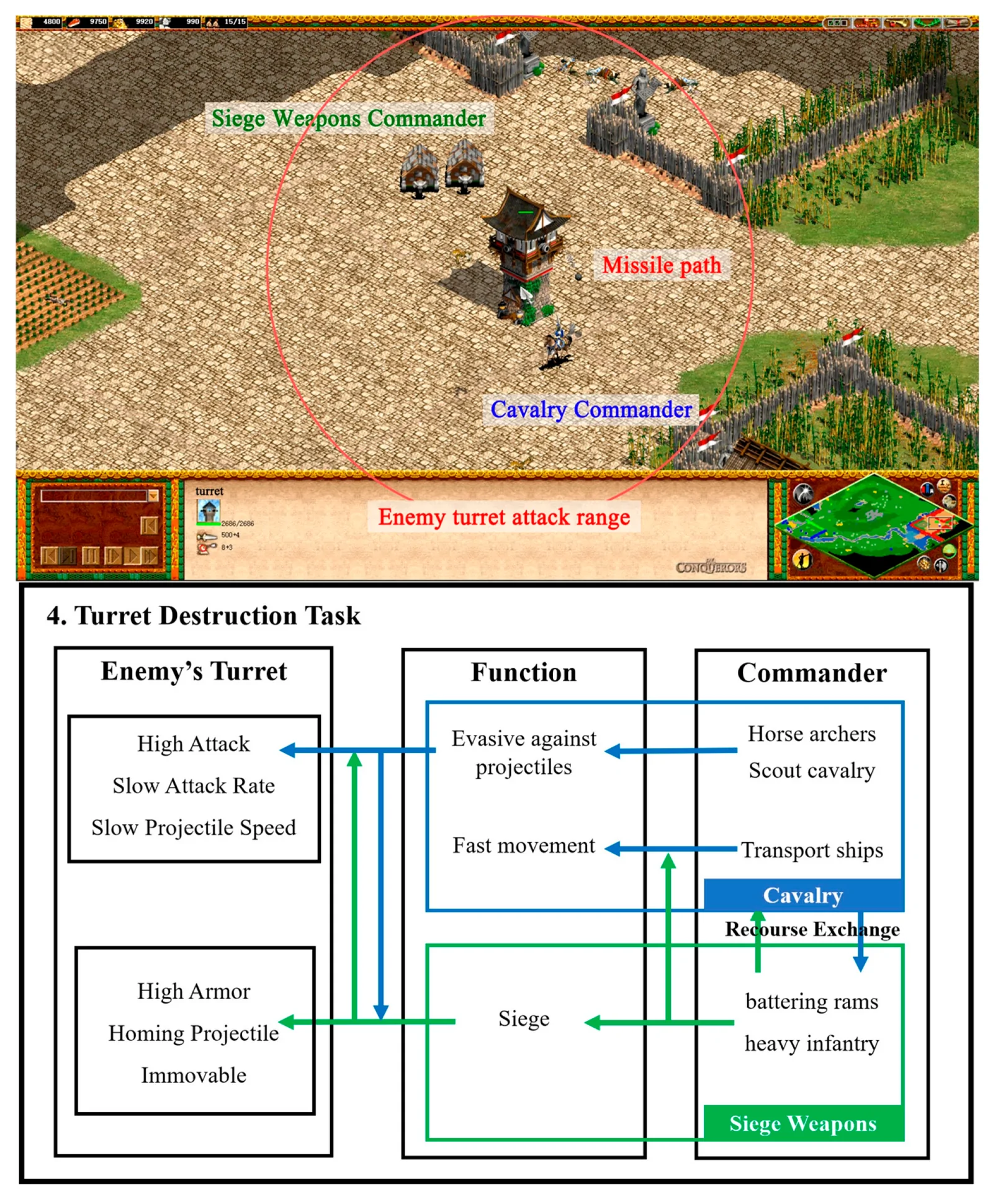
The design of collaborative logic and task graphics in the Turret Destruction Task.

It can be seen from Figure 2, the task sets clear unit attributes and constraint rules: The enemy turret has one-hit lethal damage, long attack interval and slow projectile trajectory, and will automatically track the first unit entering its attack range, and can only be damaged by siege carts; the scout cavalry and horse archers controlled by the Cavalry Commander have high movement speed, can avoid shell attacks through circular movement, but cannot cause any damage to the turret; the siege carts controlled by the Siege Weapons Commander can cause high damage to the turret, but move slowly and cannot avoid shell attacks. Under such rule constraints, the only effective solution is that the two participants carry out highly coordinated collaborative operations. The Cavalry Commander sends a single cavalry unit to attract the turret’s fire, and continuously evades attacks by using speed and trajectory prediction, while the Armored Siege Commander controls all siege carts to approach the turret from multiple directions and launch an attack, and the two sides adjust their positions and timing in real time through voice communication.

To ensure that all elements of the theoretical framework and their performances at different levels can be reflected in the assessment task, the researchers further analyzed the task characteristics as shown in Table 2. In terms of reaching team consensus, participants need to accurately understand the turret’s attack rules, their own and their partner’s role advantages and limitations, and clearly express strategic intentions, carry out perspective-taking and view negotiation; in terms of organizing team actions, participants need to formulate a coordinated attack plan based on unit characteristics, decompose the problem of destroying the turret into two complementary operational steps, and adjust the strategy in time according to the battle results; in terms of maintaining team function, participants need to maintain collective awareness, focus on task progress, and provide operational support and emotional encouragement to their partners.

Different levels of CPS competency are reflected in distinct behavioral patterns: participants with low CPS competency tend to launch blind attacks independently without communication, experience repeated failures and make no strategic adjustment; those with medium competency can carry out basic communication, but fail to coordinate operational timing and unit allocation; those with high competency can take the initiative to analyze task rules, design clear collaborative plans, and achieve precise synchronization of operations. This design realizes the accurate correspondence between task operations and CPS behavioral indicators, and provides objective and observable evidence for subsequent competency scoring.

### 3.3. Top-Level Design: Validation

The top-level design of the Collaborative Siege Task is formulated to solve the core pain points of traditional CPS tasks, such as over-simplified situations, low collaborative interdependence, and insufficient measurement of integrated cognitive-social processes. First, in terms of task positioning, this study abandons the well-structured problem scenarios commonly used in previous CPS assessments, which often reduce the authenticity of collaborative behaviors for the convenience of operation. Instead, it takes the RTS game as the basic form of the task, and constructs a dyadic collaborative task that does not involve disciplinary knowledge, relies on high interdependence, and presents an ill-structured problem. The task is set in a virtual situation with independent scenarios and information technology support, in which two participants have equal status but undertake asymmetric task roles, cannot obtain all task information at the initial stage, and need to complete the goal through real-time voice communication and observable interactive operations. This positioning ensures that the task focuses on the general CPS competency of individuals, rather than domain-specific knowledge or game operation skills, and enhances the ecological realism and generalization of the assessment.

For the selection of development platform and tools, the task is developed based on *Age of Empires II: The Conquerors (AOE2)*, a classic RTS game with high stability and open customization functions, and adopts the modified Hawk Edition SP3 (Hawk Teams, 2014) as the operating platform, with the Advanced Genie Editor (AGE) and Map Editor (ME) as core development tools. This platform selection is based on three practical and theoretical considerations: First, AOE2 has a built-in multiplayer online function and highly free map and unit editing modules, which can realize the customized design of collaborative scenarios, unit constraints and resource rules required for CPS assessment; second, the game has extremely low hardware requirements and good system compatibility, which can be applied to most computer laboratory environments, reducing the operational threshold for large-scale assessment; third, the simple and intuitive operation interface and clear unit identification avoid excessive extraneous cognitive load on participants, so that they can concentrate on cognitive processing and collaborative interaction rather than adapting to game operations.

In terms of the CPS behavior trigger mechanism, this study draws on the trigger model of collaborative problem-solving behaviors proposed by Yuan (2017), and constructs a three-dimensional integrated trigger system of shared goal, problem posing, and information asymmetry for the RTS game scenario, which can actively induce participants to carry out collaborative cognition and interaction rather than passively waiting for sporadic behaviors. Specifically, the shared goal unifies the behavioral motivation of the two participants by setting the same ultimate task objective; the problem posing creates specific collaborative dilemmas through the constraint relationship between game units and defensive facilities; the information asymmetry realizes the non-substitutability of roles through the allocation of exclusive resources and unit capabilities to the two participants, forcing them to carry out information transmission, perspective-taking, and coordinated negotiation. This trigger mechanism runs through the whole task process, ensuring that all core elements of the CPS framework can be activated and observed in specific operational situations.

## 4. A Pilot Study for the Feasibility of Collaborative Siege Task

The core objective of this pilot study is to preliminarily examine the feasibility of the Collaborative Siege Task, establish a supporting quantitative scoring rubric, and test its psychometric properties, so as to lay a solid foundation for the final development of a standardized assessment tool. Specifically, through systematic Cognitive Task Analysis (CTA), this study aims to address the following issues of the Collaborative Siege Task. First, are the actual behaviors and cognitive processes of participants completing the Collaborative Siege Task consistent with the theoretical expectations of task design? Second, what is the feasibility of the preset CPS competency indicators for the five sub-tasks, and to what extent do they align with the theoretical descriptions of the competency framework? Third, is the operating difficulty level of the Collaborative Siege Task appropriate for the target participant population, and are there any design flaws or procedural loopholes, and what targeted optimizations are required? Fourth, does the behavioral observation scoring system constructed based on the task possess favorable psychometric properties (difficulty, discrimination, correlation)?

### 4.1. Methods

**Participants.** Thirty-six adult undergraduate students were recruited from two universities in eastern China using random sampling, with an equal sex distribution (18 males and 18 females). The sample comprised 12 freshmen within their first month of enrollment, 12 sophomores and above, and 12 postgraduate students. To control for the potential effects of grade level and sex on task performance, the 36 participants were divided into 18 dyadic collaborative teams.

**Examiners and Observers.** Six postgraduate students with a major in education or psychology and experience in interview and behavioral observation research were recruited as examiners and observers. After standardized training, all personnel mastered the entire procedure of the Collaborative Siege Task, the semi-structured cognitive interview technique, and the behavioral observation scoring criteria, meeting the implementation requirements of this study.

**Collaborative Siege Task with Draft of Observation Scoring Rubric.** The Collaborative Siege Task developed by this study will be administered to 36 participants. Moreover, to explore how to score using the preset behavioral indicators with distinct level differences in Table 2, a draft observation scoring rubric was developed to accompany the Collaborative Siege Task. This rubric was scored by examiners and observers based on gameplay recordings and logs after participants completed the task. The structure of the scoring rubric was highly consistent with the factors and proficiency levels in the assessment framework. Each of the 17 elements in the framework was assessed by one item per task stage, resulting in a total of 5 × 17 = 85 observation indicators across the five sub-tasks.

As shown in Table 3, the observation rubric included three types of items: First, multiple-select items, in which all behaviors consistent with the rubric descriptions and exhibited by participants during the task were checked. Second, frequency-count items require observers to identify and record the occurrence of corresponding behaviors performed by participants according to each descriptor in the rubric. Third, forced-choice items require observers to select the most appropriate option from three alternatives based on participants’ performance. Based on the observation, the scoring methods in the last column of Table 3 were used to derive observation scores consistent with the description levels of the assessment framework, where 0 represents low proficiency, 1 represents moderate proficiency, and 2 represents high proficiency.

**Cognitive Task Rating Scale.** The cognitive task rating scale evaluates the CPS assessment indicators (see sample indicators in Table 2 and Table 3) across the five task stages by 36 participants on two dimensions: observability (the extent to which predefined behaviors can be observed by others) and consistency (the degree to which behavioral performance matches the theoretical descriptions of different CPS proficiency levels), using a three-level rating system: Good, Fair, and Poor.

**Cognitive Interview Outline.** The cognitive interview outline was used to guide six examiners in conducting cognitive interviews with 36 participants. The cognitive interview outline adopts a semi-structured design, focusing on task completion processes, difficulty, operational issues, design flaws, and optimization suggestions. It also includes in-depth follow-up questions for items rated “Poor” on the Cognitive Task Rating Scale to explore underlying causes and directions for improvement.

### 4.2. Research Procedure

Thirty-six participants were assigned to 18 collaborative dyads after balancing for sex and grade level. The 18 dyads completed the pilot test in six sessions, with three dyads per session. After task completion, two examiners separately conducted cognitive interviews with the two participants in each dyad. The pilot test was administered in a computer lab equipped with 30 Windows 7 computers, full local area network coverage, and complete headsets with microphones. The two participants in each dyad operated computers face-to-face (with no view of each other’s screens) and communicated via headsets. Both participant computers were connected to one examiner computer, allowing the examiner to record video and audio data of the gameplay process. Upon task completion, the examiner distributed the Cognitive Task Rating Scale to participants. After participants finished responding, the examiner conducted individual cognitive interviews with each participant separately.

Before the formal tests, all participants need to complete a 15 min standardized pre-task training to eliminate the performance deviation caused by individual differences in game experience. The training includes a pre-recorded teaching video demonstrating basic operations and a guided practice task with real-time voice and text prompts, so that all participants can master resource collection, unit training, map exploration, and other basic operations before the formal task, ensuring that the assessment results truly reflect CPS competency rather than game proficiency. After pre-task training, the two participants will first watch the instructional video and read the paper-based guidelines. The guidelines provide a basic description of the game task and its requirements, clarifying that their core goal is to communicate with each other, engage in coordinated operations, and jointly destroy the enemy’s red town center as quickly as possible.

The research procedure and data transcription method for the pilot test and cognitive interviews are as follows: (1) Participants watched an instructional video and then completed a practice task. (2) Participants reviewed the task instructions and then began the Collaborative Siege Task. (3) Examiners provided gameplay replay recordings and the Cognitive Task Rating Scale, and participants completed the scale based on the recordings and recall. (4) Examiners collected the completed rating scales and identified items with poor ratings as supplementary interview questions, then conducted cognitive interviews following the interview outline and recorded the interviews. (5) Within one week after the interviews, examiners transcribed the audio recordings verbatim. After transcription, examiners contacted participants to verify the accuracy of the transcribed texts.

Following completion of the pilot test and cognitive interviews, the transcribed texts were coded following the thematic analysis procedure proposed by Braun and Clarke (2006). Based on the results of the cognitive interviews, the observation scoring rubric was revised and optimized. The revised behavioral observation form for the Collaborative Siege Task was then used as the research instrument to code behaviors and obtain observation scores. To ensure consistent application of the scoring criteria, in addition to providing standardized training for six observers, the gameplay video of each participant was independently rated by at least two observers. Any items with discrepant ratings were referred to a third observer for final judgment.

Given the relatively small sample size of the pilot test, Classical Test Theory (CTT) was adopted using SPSS 25 to analyze the rating data from the observation scoring rubric, providing evidence for evaluating item quality under the proposed scoring rubric. Specifically, descriptive statistics were first conducted for each item to examine its distribution. Next, item difficulty and discrimination were assessed by calculating the passing rate, corrected item–total correlation if the item were deleted, and Cronbach’s *α* coefficient as indicators of internal consistency. Finally, Pearson product–moment correlation was used to analyze the relationships among sub-facets.

### 4.3. Results

**Matching Analysis between Cognitive Processes and Behaviors.** Overall, participants’ cognitive and behavioral performance during the Collaborative Siege Task was largely consistent with the expectations of the task design. Given the complexity of the overall design of the Collaborative Siege Task and the length of the transcribed texts, as shown in Table 4, an excerpt of interview text from one participant related to the “Turret Destruction Task” is presented to illustrate the evidence for pilot study analysis.

Taking the Turret Destruction Task as an example, it can be seen from Question 1 of Table 4 that participants knew they needed to search for the town center inside the city, though a small number forgot this objective. In any case, as their troops advanced, they inevitably came under fire from the turret and thus shifted their target to destroying the turret. When confronting the turret, participants’ first reaction was to launch a direct attack. However, the turret’s one-hit kill capability and splash damage ensured that frontal assaults would inevitably fail. Following failure, participants typically attempted to bypass the turret based on their experience from earlier siege stages. Yet the turret was placed along the only viable path by design, forcing them to attack the turret again. Participants could observe that the turret’s weaknesses included single-round firing, low rate of fire, and slow projectile trajectory. Nevertheless, most participants failed to formulate effective strategies targeting these weaknesses. For instance, some participants tried sending siege rams to attack from three directions, but the rams’ slow movement led to their elimination before approaching the turret. Similarly, some noticed that horse archers or scout cavalry could evade turret fire due to their high speed, but failed to realize that their low attack power could not effectively damage the turret. Worse still, some participants assumed that heavy infantry, as a hidden unit, could withstand shell attacks. Of course, a small number of participants devised targeted strategies. They used the high mobility of cavalry to draw and evade turret fire, then sent siege rams to destroy the turret at close range. Thus, task difficulty increased markedly in this stage.

In summary, efficient coordination between cavalry and siege rams was the only way to destroy the turret and proceed to the next stage. This aligned with task design expectations and successfully elicited participants’ behavioral manifestations in challenging tasks that required collaboration to complete. These findings provide preliminary support for the gamified task design approach proposed in this study, suggesting that it is broadly aligned with the assessment framework. Research Question 1 is therefore addressed at the pilot stage, pending further validation in the formal study.

**The Feasibility of the Preset CPS Competency Indicators.** Based on an analysis of consistency and observability responses on the Cognitive Task Rating Scale, the average number of “Poor” ratings selected by the 36 participants across the 17 indicators were 2.1, 2.8, 3.9, 3.4, and 3.1 for consistency, and 2.5, 3.4, 5.7, 6.2, and 5.1 for observability in the five stages respectively. Combined with the interview transcripts from Question 4 in Table 4, the preset indicators demonstrate generally good consistency with the theoretical framework, yet several observability problems were identified. First, observation formats varied across behavioral indicators; the dialog-dependent indicators, such as perspective-taking, might repeat multiple times within a stage, whereas indicators such as goal setting might occur only once. Using mere occurrence frequency as the observation outcome for behavioral indicators was, therefore, inappropriate. Second, although concrete examples were provided for dialog-dependent indicators such as perspective-taking, observers struggled to infer the essential differences between proficiency levels from these examples alone. Examples should thus serve only as training materials for observers rather than as observation criteria themselves. Third, indicators such as outcome evaluation and team support required specific conditions (e.g., if the task encounters difficulties) to be observable; judgments were impossible when such conditions were unmet, necessitating supplementary rules. Fourth, some in-game parameters negatively affected observation results and required adjustment and optimization. For example, an overly large splash radius increased the error rate. At this point, the behavioral indicator extraction method proposed in this study, which is consistent with the assessment framework, has been partially verified, and Research Question 2 is accordingly addressed.

**The Operating Difficulty and Design Flaws Analysis.** Regarding problem difficulty, it can be seen from Question 2 and 3 of Table 4 that the five stages of the Collaborative Siege Task increased progressively in difficulty. Nevertheless, the vast majority of participants reached at least Stage 3, and approximately one-seventh completed all stages, indicating that the overall task difficulty was appropriate for the sample. In terms of operational difficulty, participants generally found the task easy to operate; all actions required only left and right mouse clicks, with low demands on frequency or skill. Participants commented that operations were “similar to Happy Landlord” and “much simpler than Honor of Kings”. Regarding communication difficulty, most participants reported smooth and trouble-free interaction with teammates, except for a few who noted hardware issues such as low headset volume or intermittent sound.

Analyses of the pilot test and cognitive interviews revealed no critical design flaws or procedural loopholes in the Collaborative Siege Task developed in this study, though some details required refinement. These included excessively slow movement speed of siege rams, moderate speed increases for transport ships, adjusted health or armor for selected units, revised attack ranges for ranged units, reduced turret splash radius, and resource exhaustion (e.g., wood) under heavy casualties with no means of replenishment. These issues were systematically revised and optimized in the present study.

**CTT-based Psychometric Analysis.** Descriptive statistics showed that the distribution ratio of scores 0, 1, and 2 was approximately 2:5:3. Ratings for most items clustered at the moderate level, and the proportion of high-level scores was slightly higher than that of low-level scores. This distribution matched the study’s expectations for low-, moderate-, and high-level performance with no extreme skewness. Furthermore, the 85 items showed moderate item difficulty (percent score: *M* = 0.53, *SD* = 0.11) and relatively high discrimination (item–total correlation: *M* = 0.45, *SD* = 0.16), indicating moderate positive correlations. Corrected Cronbach’s *α* values after item deletion were mostly lower than the *α* values of the corresponding sub-facets, suggesting that the items contributed positively to test consistency. Except for the peer relationship sub-facet (*α* = 0.65), *α* values for the other five sub-facets ranged from 0.81 to 0.88, meeting basic psychometric standards. Meanwhile, 8 of the 85 items showed low discrimination, with correlation coefficients between 0.02 and 0.22. These items were primarily from the Team Support and Task Performance sub-facets, where behavioral variability was limited due to the collaborative nature of the task. Given the small pilot sample and the absence of substantial negative impacts on overall reliability, these items were retained for the formal study. The relatively low internal consistency for peer relationship (*α* = 0.65) may be attributed to the small number of items and low item discrimination within this sub-facet.

Total scores for each sub-facet were obtained by summing item scores, and correlational analyses were conducted to examine relationships among sub-facets. As shown in Table 5, the two sub-facets under each main facet were significantly and moderately positively correlated (*r*_21_ = 0.41, *r*_34_ = 0.54, *r*_56_ = 0.59). Sub-facets under different main facets also showed mostly weak to moderate positive correlations (some coefficients were non-significant due to small sample size), consistent with the basic logic of the study’s assessment framework.

## 5. Test Quality and Result Analysis of Collaborative Siege Tasks

This study aims to conduct a systematic validation of the gamified assessment tool for CPS competency set within the Collaborative Siege Task, and to address the research questions proposed in the Section 1.

### 5.1. Methods

**Participants, Examiners, and Observers.** A total of 312 undergraduate students were recruited from a university in eastern China using convenience sampling. Due to equipment malfunctions and withdrawal of participation, valid data were finally obtained from 298 participants, with an effective response rate of 95.51%. The sample size was determined based on the requirement of item response theory analyses, for which a minimum of 200 respondents is generally recommended (Baker & Kim, 2004), and the final sample of 298 exceeded this threshold. Specifically, among the 298 valid participants, 119 were male and 179 were female, yielding a sex ratio of approximately 2:3. There were 223 freshmen and 75 sophomores, representing a grade ratio of roughly 3:1. In terms of majors, 90 were liberal arts students, 92 were science students, 97 were engineering students, and 19 were from other majors, resulting in a major ratio of about 3:3:3:1. To balance participant willingness and sex composition, the 298 participants were divided into 149 collaborative dyads (two participants per dyad). These included 45 male–male dyads (30%), 29 male-female dyads (20%), and 75 female–female dyads (50%). To minimize testing and observation bias, the six examiners and observers from the pilot study continued to serve as test administrators and observers in the formal assessment.

**Collaborative Siege Task with Observation Scoring Form.** The optimized Collaborative Siege Task developed by this study will be administered to 298 participants. Moreover, the observation scoring form optimized from the pilot study was applied to accompany the Collaborative Siege Task, and the data from a total of 5 × 17 = 85 observation indicators across the five sub-tasks will be analyzed. The complete scoring form can be seen in Supplementary Materials C.

**CPS Peer Assessment Questionnaire.** The CPS Peer Assessment Questionnaire was developed by Yuan (2017) and contains 2 dimensions and 19 multiple-choice items, including 11 items for the problem-solving dimension and 8 items for the collaboration dimension. Data for this questionnaire were obtained from mutual ratings by two participants in the same collaborative dyad after task completion. The problem-solving ability and collaboration ability of each participant were derived by summing item scores within each dimension. The questionnaire demonstrated satisfactory internal consistency and reliability. Evaluated using Cronbach’s *α* and maximal reliability *H*, reliability coefficients for the problem-solving dimension ranged from 0.700 to 0.788, and those for the collaboration dimension ranged from 0.761 to 0.821. This questionnaire served as a key criterion for validating the gamified assessment results.

**Raven’s Advanced Progressive Matrices (Short Form).** The short-form Raven’s Advanced Progressive Matrices was adapted and revised from the original scale by Arthur and Day (1994), consisting of 12 dichotomous (0–1 scored) figural reasoning items. Participants’ figural reasoning ability was calculated as the total score across all items. The short-form scale showed good reliability; based on Cronbach’s *α* and maximal reliability *H*, its internal consistency reliability ranged from 0.735 to 0.876. In CPS research, figural reasoning is commonly used to examine the relationship between problem-solving ability and general intelligence, with a significant positive correlation typically reported in prior studies.

**Mini-IPIP Big Five Personality Inventory.** The Mini-IPIP Big Five Personality Inventory was developed by Gosling et al. (2003), including 10 adjective self-report items rated on a 7-point Likert scale (0–6) (e.g., anxious, easily discouraged). Participants rated the extent to which each adjective described themselves, with higher scores indicating greater self-descriptiveness. The inventory measures five dimensions: Extraversion, Agreeableness, Conscientiousness, Emotional Stability, and Openness. It is designed for brief, albeit less precise, personality assessment, with relatively modest reliability. Evaluated using Cronbach’s *α* and maximal reliability *H*, internal consistency coefficients for the five dimensions were 0.770–0.861 (Extraversion), 0.614–0.760 (Agreeableness), 0.466–0.531 (Conscientiousness), 0.526–0.605 (Emotional Stability), and 0.626–0.675 (Openness), respectively. In CPS research, Agreeableness from the Big Five is typically positively correlated with the collaboration dimension.

**Online Game Flow Experience Scale.** The Online Game Flow Experience Scale was developed by Huang and Zhu (2021), using 7 self-report items rated on a 5-point Likert scale (0–4) to measure participants’ hedonic experience in game contexts. Higher scores indicated a better gaming experience. The scale demonstrated excellent internal consistency reliability; based on Cronbach’s *α* and maximal reliability *H*, reliability coefficients ranged from 0.927 to 0.958. In this study, flow experience was used to examine the relationship between gamified assessment outcomes and gaming experience, with a non-significant or weak positive correlation expected.

**Self-Developed RTS Game Experience Questionnaire.** This questionnaire consisted of 21 five-point Likert items, with the total score used to index participants’ RTS game experience (higher scores indicated greater experience). It was mainly used to assess the relationship between CPS competency and game experience. The example items were as follows: (1) How often do you play RTS games on average? (2) How long do you usually play RTS games per session on average? (3) How skilled are you at playing RTS games? The Cronbach’s *α* of this questionnaire is 0.916.

### 5.2. Research Procedure

The formal test was conducted in the same computer laboratory as the pilot study. The 149 dyads were tested in 15 sessions, with 8 to 11 dyads per session. On average, each observer was responsible for testing and data collection for two dyads, including video and audio recordings of gameplay and questionnaire data. In contrast to the pilot study, in which examiners provided prompts when necessary, no assistance other than addressing technical issues was given during the formal test. Upon completion of the game task, participants were asked to complete the following five validity questionnaires: the CPS Peer Assessment Questionnaire, Raven’s Advanced Progressive Matrices (Short Form), Mini-IPIP Big Five Personality Inventory, Online Game Flow Experience Scale, and the Self-Developed RTS Game Experience Questionnaire.

After data collection for all 149 dyads was completed, the six observers conducted behavioral observations and rating assessments on the gameplay videos using the Observation Scoring Form. To ensure consistent application of the scoring rubric, in addition to standardized observer training, each participant’s video was rated independently by at least two observers. Any items with discrepant ratings were referred to a third observer for final adjudication. Subsequently, the scoring rules presented in Table 3 were applied to derive polytomous rating data (three levels: 0, 1, 2) for subsequent statistical analyses.

### 5.3. Data Analysis

First, within the framework of IRT, the item quality and test reliability of the gamified assessment tool are systematically analyzed to verify its reliability. Based on rating data from the behavioral observation form, a Bayesian two-level multidimensional partial credit model in Equations (1) and (2) is applied to establish the relationships between items and sub-facets (first level), as well as the relationship between items and elements (second level). Specifically, the mathematical expression for the first level of the model is as follows:

(1) $$\begin{array}{l} P_{jx}\left(\theta_{i}\right)=P\left(X_{ij}=x,q_{j}|\theta_{i},b_{j},d_{j,0},d_{j,1},d_{j,2}\right)\\ =\frac{\exp\left(x\left(\theta_{i}^{T}q_{j}-b_{j}\right)-\sum_{l=0}^{x}d_{j,l}\right)}{\sum_{k=0}^{2}\exp\left(k\left(\theta_{i}^{\prime }q_{j}-b_{j}\right)-\sum_{l=0}^{k}d_{j,l}\right)}, \end{array}$$

where $P_{jx}\left(\theta_{i}\right)$ denotes the probability that the participant $i\left(i=1,2,\ldots I\right)$ with ability (6 sub-facets) $\theta ={\left[\theta_{1},\theta_{2},\ldots ,\theta_{V}\right]}^{T}$ obtains a score of $x\in \left[0,1,2\right]$ on the item $j\left(j=1,2,\ldots ,J\right)$; $q_{j}=\left[q_{j1},q_{j2},\ldots ,q_{jV}\right]$ indicates whether the item j measures the ability in θ, with a value of 1 indicating measurement and 0 indicating no measurement. Since each item in this study measures only one ability, $\theta_{i}^{T}q_{j}$ effectively identifies which type of ability in $\theta_{i}^{T}$ the participant i uses to respond to item j; $b_{j}$ is the difficulty parameter of item j, and $d_{j,0},d_{j,1},d_{j,2}$ represent the threshold parameters for item j corresponding to scores of 0, 1, 2, respectively. For model identification, the constraints $d_{j,0}=0$ and $d_{j,2}=-d_{j,1}$ are imposed.

The mathematical expression for the second level of the model is as follows:

(2) $$\begin{array}{l} b_{j}\sim N\left(\mu_{bI\left(j\right)},\sigma_{bI\left(j\right)}\right),\;d_{j,0}\sim N\left(\mu_{dI\left(j\right),0},\sigma_{dI\left(j\right),0}\right),\\ d_{j,1}\sim N\left(\mu_{dI\left(j\right),1},\sigma_{dI\left(j\right),1}\right),\;d_{j,2}\sim N\left(\mu_{dI\left(j\right),2},\sigma_{dI\left(j\right),2}\right), \end{array}$$

where μ and σ represent the mean and variance of the normal distribution hyperparameters governing the item parameters, respectively. $I\left(j\right)$ is an indicator function indexed by item j, specifying which elements the item j belongs to. For example, suppose there are 3 items labeled A1–A3, where A1–A3 belong to Element A. The difficulty parameters of A1-A3 follow a normal distribution with mean $\mu_{b\left(A\right)}$ and variance $\sigma_{b\left(A\right)}$, and researchers can then evaluate the central tendency and dispersion of each element using μ and σ. Specifically in this study, due to model identification constraints and the 3-point scoring scheme with $d_{j,0}=0$ and $d_{j,2}=-d_{j,1}$, the second level of the model only involves the distributions of two types of parameters: $b_{j}$ and $d_{j,1}$. Following Johnson and Sinharay (2005), this study used the rstan (version 2.26.11) package (Stan Development Team, 2022) to obtain parameter estimates via the No-U-Turn Sampler (NUTS). To ensure estimation accuracy, following Gelman and Rubin (1992), four Markov chains were constructed, with $\hat{R}<1.1$ as the convergence criterion. After obtaining item parameters, the custom model function in the mirt package (Chalmers et al., 2016) was used to import estimates from rstan, and further compute item discrimination, model fit indices, etc.

The reliability analysis within the IRT framework is generally based on the Fisher Information (FI) function. On the one hand, FI can be used to calculate the test standard errors for each participant in Equation (3), and the empirical reliability of the entire test can then be derived using the ability estimation errors across all participants in Equation (4). On the other hand, FI can also directly indicate the total reliability of the test, thereby determining the ability range for which the test is most suitable.

(3) $$FI\left(\theta \right)=\sum \frac{{\left[P_{j}\left(\theta \right)\prime \right]}^{2}}{P_{j}\left(\theta \right)\left(1-P_{j}\left(\theta \right)\right)},\;SE\left(\theta \right)=\frac{1}{\sqrt{I\left(\theta \right)}},\;$$

(4) $$r\left(\theta \right)=1-{\left[SE\left(\theta \right)\right]}^{2},\;r=E\left[\int r\left(\theta \right)d\theta \right].$$

Then, a higher-order factor model is used to establish the internal connections among sub-facets, main facets, and overall CPS competency via Mplus 8.4. The structural validity of the assessment tool is examined via CFA, while the structure of the CPS competency assessment framework is validated and refined, providing empirical support for the framework. Third, the criterion-related validity of the gamified CPS assessment is examined through correlation analysis with criteria, including peer assessment, Raven’s reasoning, and agreeableness, strengthening its validity evidence. Finally, the applicability of the Collaborative Siege Task to participants of different proficiency levels is verified by analyzing the matching between demographic characteristics and participant ability estimates, offering theoretical and empirical support for the widespread application of the assessment tool.

### 5.4. Results

**Item and Element Quality Analysis.** The complete item parameters are presented in Supplementary Materials D. Overall, the 85 items demonstrated favorable discrimination, with item–sub-facet correlations (${\bar{r}}_{xy}$) ranging from 0.20 to 0.82 and a mean of 0.59 (*SD* = 0.11). Items with relatively lower discrimination were predominantly concentrated in Task 5. This was partly attributable to the nature of these items, as many belonged to the Team Support and Task Performance sub-facets where behavioral variability was inherently constrained by collaborative task demands. It was also partly due to data missingness, given that 13.4% and 20.8% of participants failed to reach Task 4 and Task 5, respectively. The model fitted well, with infit and outfit mean square values ranging from 0.52 to 1.24. The item difficulty parameters ($\bar{b}$) had a mean of 0.04 (*SD* = 0.67, range: −2.09 to 1.62), indicating a moderate overall difficulty level. The step thresholds (${\bar{d}}_{1}$) were predominantly negative (mean = −1.48, *SD* = 0.92, range: −4.59 to 0.05), suggesting that participants could generally obtain a score of 1 without difficulty, whereas reaching score 2 required higher ability.

Table 6 presents the element quality analysis results based on the formal test sample. Consistent with item difficulty, the average difficulty of the 17 elements was moderate. Specifically, Behavioral Norms, Outcome Evaluation, View Expression, and Team Support showed relatively low difficulty, while Perspective-Taking, Problem Deconstruction, and Resource Management exhibited relatively high difficulty. In addition, the variance parameters differed substantially across facets. On the one hand, variances for facets including View Expression, View Negotiation, and Cooperativeness were greater than 1, indicating large differences in difficulty among items within these elements, and that item difficulty was strongly affected by task variation. On the other hand, variances for elements such as Problem Decomposition and Resource Management were less than 0.1, suggesting negligible difficulty differences among items within these elements; item difficulty was driven mainly by the elements themselves.

**Figure 3:**
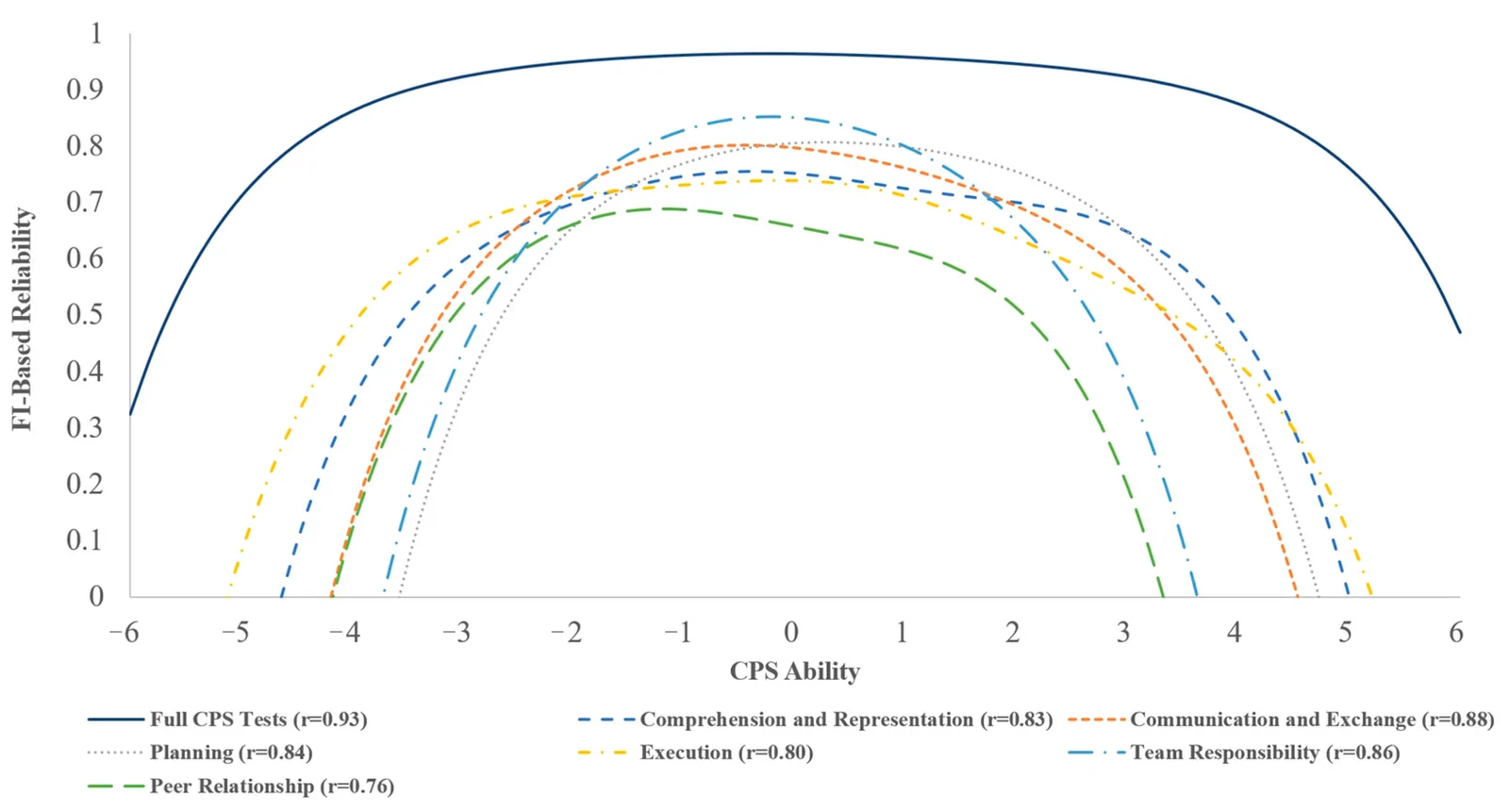
The FI-based reliability with empirical reliability and for the six sub-facets.

**Fisher Information based Reliability Analysis.** As shown in Figure 3, the empirical reliability of the six sub-facets ranged from 0.76 to 0.88. Except for the Team Support sub-dimension, which showed slightly lower reliability due to a smaller number of items, the reliability values of all other sub-facets were above 0.80, and the reliability of full CPS test were above 0.90, indicating satisfactory internal consistency reliability of the assessment. In addition, the figure reveals that the Collaborative Siege Task is primarily suitable for measuring participants with ability levels between −2 and +2. Measurement error tends to increase for participants with extremely high or extremely low ability levels. Moreover, the Cronbach’s *α* for the entire test is 0.933, and the coefficients for the 6 sub-facets are 0.839, 0.914, 0.835, 0.746, 0.869, and 0.782, respectively.

**Test Structure Validity Analysis.** The factor analysis is typically based on participants’ item responses and analyzes the relationships between items and dimensions, as well as among dimensions, using the covariance structure among items. However, the assessment framework adopted in this study is relatively complex, consisting of a four-level structure: main facet–sub-facet–element–item. Modeling directly at the item level would likely result in poor model fit due to constraints related to sample size, degrees of freedom, and other factors.

Given that the relationships among sub-facets, elements, and items were already established using a Bayesian two-level multidimensional partial credit model, and to simplify the model for practical analysis, this study conducted structural analyses based on sub-facet scores (*θ*).

**Figure 4:**
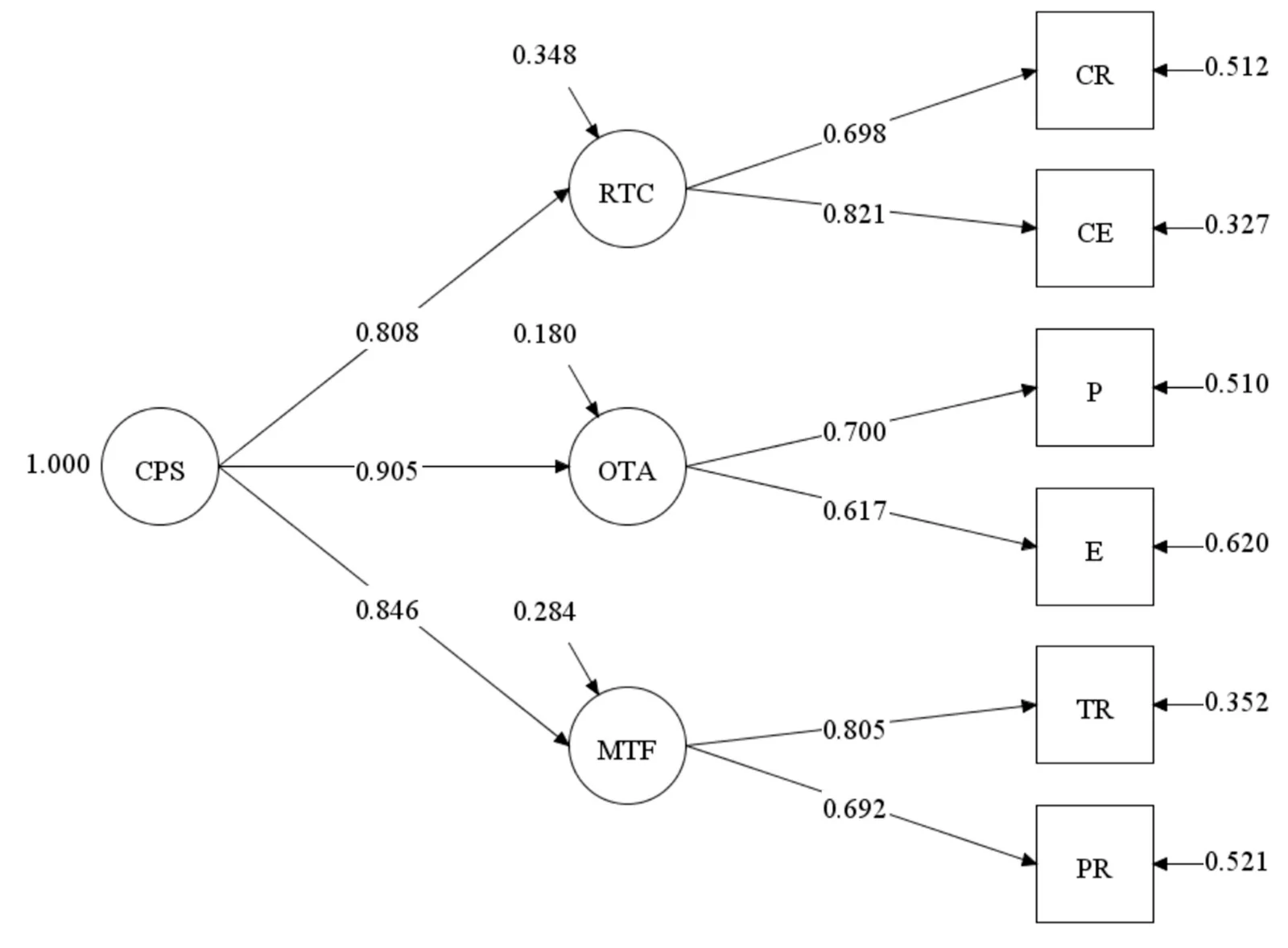
Factor loadings of the higher-order factor structure (Model 2). Note. CPS for collaborative problem solving, RTC for Reaching Team Consensus, OTA for Organizing Team Actions, MTF for Maintaining Team Function, CR for Comprehension and Representation, CE for Communication and Exchange, P for Planning, E for Execution, TR for Team Responsibility, PR for Peer Relationship.

As shown in Table 7, four competing models were compared. The bi-factor model was discarded because it failed to converge during parameter estimation. Among the remaining three models, the one-factor model yielded the poorest fit, while the three-factor model and the higher-order factor model produced nearly identical and acceptable fits to the sample data. Given that the higher-order factor model and the three-factor model had the same degrees of freedom, the higher-order factor structure (Model 2) was retained for greater interpretability. This structure indicates that the three main dimensions can be accounted for by a higher-order general CPS competency factor. The standardized factor loadings are presented in Figure 4. Thus, the consistency between the data structure of participants’ behavioral responses in the task and the theoretical structure of the assessment framework is supported by the empirical evidence, and Research Questions 2 and 3 are therefore addressed.

**Figure 5:**
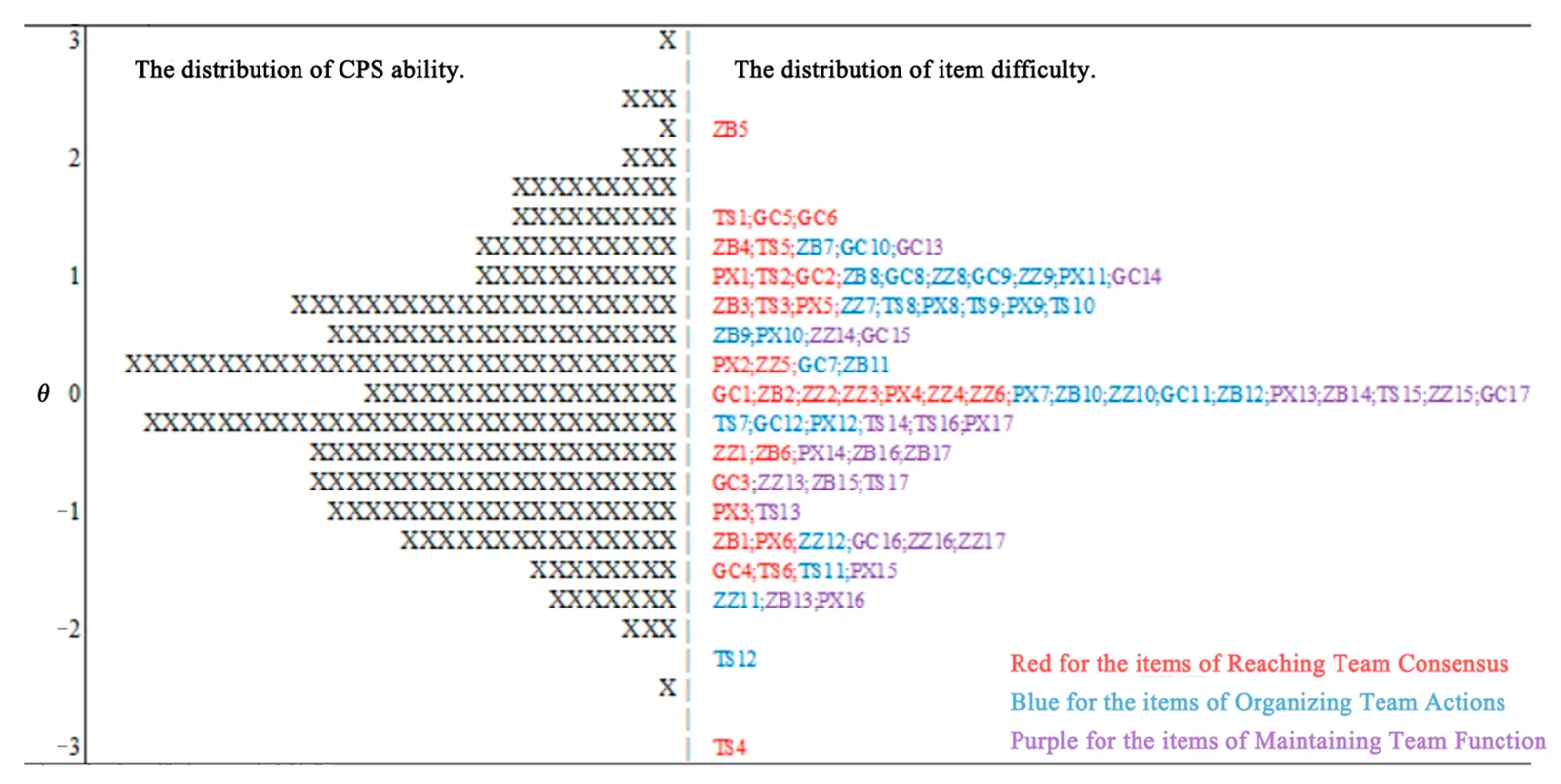
Distribution of CPS ability and item difficulty (each X represents 1.17 participants).

**Analysis of Testing Matching.** Figure 5 illustrates the matching analysis between participants’ overall CPS ability and item difficulty. Overall, participants’ ability followed an approximately normal distribution, and item difficulty adequately covered participants across different ability levels, indicating a satisfactory overall matching degree. In detail, items related to reaching team consensus were evenly distributed across the entire difficulty spectrum. Items concerning organizing team actions were generally more difficult, whereas items associated with maintaining team functioning tended to be easier. This pattern suggests that reaching team consensus serves as the foundation of collaborative problem solving; organizing team actions acts as a critical indicator distinguishing individual ability levels; and maintaining team functioning plays a foundational supporting role in the collaborative process.

**Criterion-Related Validity Analysis.** Criterion-related validity was examined using the CPS competency factor scores derived from the higher-order factor model. Table 8 presents the correlations between CPS competency and all criterion variables. The table consists of upper and lower triangular matrices: the lower triangle shows Pearson correlation coefficients, while the upper triangle displays partial correlation coefficients after controlling for game flow experience and RTS game experience.

As shown in the lower triangle, CPS competency was moderately, positively, and significantly correlated with both collaboration ability and problem-solving ability measured by the peer assessment questionnaire. Weak but significant positive correlations were also found between CPS competency and Raven’s reasoning, agreeableness, game flow experience, and RTS game experience. This suggests that although CPS competency assessed via the gamified task shows acceptable convergence with peer ratings, figural reasoning, and agreeableness, such convergence might be partly attributable to game experience and game-related flow. Thus, further examination of criterion-related validity was warranted. Accordingly, partial correlations controlling for game experience and flow experience were computed. As indicated in the upper triangle, the significant associations between CPS competency and peer-rated collaboration and problem-solving remained robust. The correlations with figural reasoning and agreeableness decreased slightly but remained at least marginally significant. These partial correlations, which controlled for potentially confounding gaming experience variables, essentially served a similar purpose to regression-based approaches in isolating the unique contribution of the assessment.

To further examine the joint contribution of these criterion variables, we conducted a multiple regression analysis with CPS competency as the dependent variable and all criterion and demographic variables as predictors. Peer-rated collaboration (*β* = 0.362, *p* < 0.001) and problem-solving (*β* = 0.204, *p* < 0.001) emerged as the strongest predictors, providing robust support for criterion-related validity. Agreeableness (*β* = 0.137, *p* < 0.01) and conscientiousness (*β* = 0.122, *p* < 0.05) also showed significant positive effects, consistent with theoretical expectations. Importantly, RTS game experience (*p* = 0.446) was not a significant predictor, suggesting that the assessment captures CPS competence rather than game proficiency. Raven’s reasoning was also not significant (*p* = 0.208), reinforcing that the tool measures CPS as an integrated construct rather than individual reasoning ability. Demographic variables (sex, grade, major) were all non-significant (*p*s > 0.05), supporting the fairness of the assessment across subgroups. Taken together, these findings demonstrate that CPS competency measured by the collaborative siege task possesses satisfactory criterion-related validity. Thus, Research Question 4 is addressed.

**Demographic Variable Analysis.** The demographic differences in CPS competency assessment across three variables: sex (male, female), major (liberal arts, science, engineering, other), and game role (cavalry commander, siege weapon commander) will be examined via a three-way ANOVA. As shown in Table 9, no significant differences were found in CPS competency for sex, major, game role, or their interaction effects. Based on the statistical results of the current sample, the collaborative siege task does not produce significant systematic bias for participants with different sexes, academic backgrounds, or game roles, indicating a wide applicability of the assessment.

## 6. Conclusions and Discussion

### 6.1. Main Conclusions

This study developed and provided empirical support for a theory-driven, game-based assessment of collaborative problem solving. The theoretically grounded CPS competency framework proposed by Guo and Yang (2025) receives consistent empirical support. The higher-order factor structure includes three core facets and six sub-facets. It presents an acceptable model fit and matches closely with the data structure obtained from game-based behavioral responses. The integrated and process-oriented framework works well for real human–human interaction in higher education.

The Collaborative Siege Task shows strong feasibility and provides support for its ecological validity. It maintains close alignment with the established assessment framework. The design uses asymmetric roles and ill-structured problem settings along with a progressive five-stage structure. These elements consistently bring about the complete range of CPS behaviors specified in the assessment rubric. Participants find the task easy to follow with little unnecessary cognitive burden. The difficulty level is appropriate for the target population. These qualities confirm that the task can be applied effectively in large-scale assessment.

A qualitative-oriented scoring approach is adopted in this study. The approach focuses on behavioral proficiency levels instead of simple behavior frequency counts. It captures valid and meaningful evidence related to CPS performance. A total of 85 observation indicators are included in the IRT framework. These indicators show moderate difficulty and high discrimination power. They also achieve satisfactory reliability values. This scoring method avoids the common problem of overusing quantitative frequency counts. Such overuse often reduces the interpretability of measurement models, as noted in prior methodological critiques.

Strong validity evidence supports the use of this gamified assessment tool. CPS scores show meaningful and significant correlations with peer-rated collaboration and problem solving. They also correlate with reasoning ability and agreeableness in expected directions. The scores show no meaningful variation related to sex, major, or in-game role assignment. These findings confirm that the tool possesses satisfactory criterion-related validity. The tool is fair and generalizable across different subgroups of undergraduate students. Additional analysis of ability and difficulty matching reveals a well-calibrated assessment system. The difficulty range of items covers the full spectrum of participant ability levels. Specifically, Organizing Team Actions stands out as the main feature that distinguishes different proficiency levels, and maintaining Team Function acts as a fundamental supporting component. This pattern is consistent with the hierarchical development logic of collaborative problem-solving competence.

### 6.2. Discussion and Implications

Beyond providing empirical support for the framework, this study provides a new perspective for a core theoretical contradiction in CPS measurement, the structural validity paradox. Previous empirical investigations have yielded conflicting factor structures, some supporting a two-factor collaboration vs. problem-solving model (Griffin et al., 2012; Yuan, 2017), others a bi-factor or three-factor model with a dominant general factor (He, 2019; Sun et al., 2020). By tightly anchoring task design to theoretical specifications at the indicator level, the current research produced a stable, replicable factor structure that matches theoretical predictions. This outcome underscores a key theoretical insight that inconsistent structural validity in prior CPS research stems not from inherent instability of the CPS construct, but from misalignment between assessment frameworks, task design, and evidence extraction. When the three core components of the evidence-centered design (ECD) competency model, task model, and evidence model are coherently integrated, the underlying structure of CPS becomes empirically discernible. This underscores what this study considers to be the central contribution of the present work. It is not the game environment per se, but rather the demonstration that CPS can be validly measured when theoretical specification, task design, indicator extraction, and psychometric modeling are brought into coherent alignment. The study also extends evidence-centered design theory by operationalizing a qualitative, level-based scoring paradigm to replace the dominant frequency count approach. As emphasized in methodological critiques (Sun et al., 2020), scoring CPS by behavioral frequency distorts construct meaning high-frequency behaviors overwhelm low-frequency but high-value behaviors, reducing model interpretability. The present rubric, which scores performance against three proficiency levels (low/medium/high) rather than sheer occurrence, captures the quality of collaboration rather than mere quantity. This approach not only improves the validity of scoring but also provides a theoretical foundation for diagnostic CPS assessment, an urgent direction for the field as measurement shifts from summative evaluation to formative support.

For educational assessment practice, the Collaborative Siege Task establishes a more realistic model for gamified CPS measurement that balances ecological validity and administrative feasibility. While the current implementation relies on manual observation and coding, the task design and standardized procedure make it well-positioned for future automation. The use of off-the-shelf game engines and the systematic nature of the behavioral indicators provide a foundation upon which machine learning-based scoring can be built, which would be a critical step toward achieving large-scale deployment. Traditional computer-based CPS assessments require bespoke simulation engines and high development costs (Stoeffler et al., 2020), limiting widespread adoption. By repurposing the stable, widely accessible platform Age of Empires II with custom map and rule editing, this study demonstrates that more realistic, ill-structured collaborative tasks can be implemented using off-the-shelf tools, lowering technical barriers for schools and researchers. The task’s asymmetric role design (Cavalry Commander/Siege Weapons Commander) creates compulsory interdependence, ensuring that participants cannot succeed through individual effort alone, which is a critical feature often missing in weakly structured CPS tasks.

The assessment yields practical diagnostic utility by distinguishing differential difficulty across CPS components. Item-level analyses revealed that Organizing Team Actions (Planning, Execution) was the most difficult dimension, while Maintaining Team Function (Team Responsibility, Peer Relationship) was relatively easier. This pattern carries clear instructional implications: educators should prioritize training in goal setting, problem decomposition, and coordinated execution, while treating team cohesion and communication as foundational supporting skills. Furthermore, the absence of significant sex, major, or role-related bias confirms the tool’s fairness for large-scale assessment, supporting its use in program evaluation, admissions screening, and workplace recruitment contexts where CPS is increasingly a key criterion (O’Neill et al., 2012).

### 6.3. Limitations and Future Directions

Several limitations should be noted. The sample was convenience-sampled from one eastern Chinese university and predominantly comprised first-year undergraduates (74.8%), which may restrict generalizability to broader age groups and educational levels. Participants also came from six provinces, alleviating concerns of extreme regional homogeneity to some extent, and the sample size (*N* = 298) met the recommended threshold for IRT analyses. Future studies should recruit more balanced samples across grade levels and educational contexts to strengthen cross-population generalizability. Second, the CPS assessment was dyadic (two-person), whereas real-world teamwork often involves larger groups. The generalizability to larger teams remains to be examined. Third, although scoring was based on behavioral quality, manual observation and coding were labor-intensive, which currently limits the scalability of the approach. Future work should integrate automated behavioral logging and natural language processing to implement real-time, unobtrusive stealth assessment and explore NLP-driven automated scoring for collaborative discourse. Fourth, the study focused on assessment rather than instruction. While the tool identifies CPS profiles, it does not yet include embedded feedback or adaptive learning paths to foster growth.

Based on the findings, four lines of future research are proposed. First, expand the assessment to larger teams (3–5 members) and explore CPS dynamics in larger groups, including leadership, role differentiation, and subgroup interactions. Furthermore, in the era of artificial intelligence, it is also worth considering incorporating intelligent agents as collaborative partners and studying the mechanism of multi-party collaboration between humans and intelligent agents. Second, develop automated scoring using machine learning on process data (operation logs, voice dialog, eye movement) to achieve efficient and consistent scoring, which would be essential for realizing the full scalability potential of the assessment. A feasible approach would involve extracting time-stamped action sequences from game logs and applying NLP techniques to transcribe and code voice communications for key collaborative features such as perspective-taking and negotiation. This would enable continuous, data-driven scoring without interrupting task flow, reduce reliance on manual observation, and provide immediate feedback to support formative assessment. Third, integrate assessment with intervention by designing adaptive game tasks that provide real-time feedback and scaffold CPS development, creating a unified “assess-and-promote” system. Fourth, conduct cross-cultural and longitudinal studies to examine the invariance of the CPS framework across cultures and the developmental trajectory of CPS competencies throughout undergraduate education.

## Figures and Tables

**Table 1:** Guo and Yang (2025)’s CPS competency assessment framework.

Facet	Sub-Facet	Element
Reaching Team Consensus	Comprehension & Representation	Task context, own role, peer roles
	Communication & Exchange	View expression, perspective-taking, and view negotiation
Organizing Team Actions	Planning	Goal setting, problem decomposition, and resource management
	Execution	Task performance, coordination, outcome evaluation
Maintaining Team Function	Team Responsibility	Collective awareness, concentration, and task advancement
	Peer Relationships	Behavioral norms, team support

Note: See Supplementary Materials A for detailed mappings.

**Table 2:** Illustrative examples of CPS level-based task performance in the Turret Destruction Task.

Elements with Indicators	Task Performance at Different CPS Levels
**Own Role**: Degree of understanding of the role of one’s own role in collaboration	**For Cavalry Commander:** **Low level:** Misunderstands or fails to recognize the role of horse archers and scout cavalry. **Medium level:** Regarding the role of scout cavalry and horse archers in drawing fire and evading turret shells. **High level:** On the basis of the medium level, uses a single unit to probe the turret’s attack pattern. **For Armored Commander:** **Low level:** Misunderstands or fails to recognize the role of heavy infantry. **Medium level:** Recognizes that only siege vehicles need to be built to destroy the turret. **High level:** On the basis of the medium level, commands siege vehicles to assault the turret simultaneously from three directions.
**View Expression:** Expression of one’s own viewpoints or opinions	**The discussion is limited to resource exchange, unit formation, and turret attack.** **Example:** Intending to probe the turret’s attack pattern with teammates. **Low level:** Takes no initiative to propose; only agrees passively if the teammate proposes first. **Medium level:** Says, “You go attack the turret and test it first.” **High level:** Says “Cavalry is fast; send cavalry first to scout the turret’s firepower.”
**Problem Deconstruction:** Level of analyzing problems, exploring ideas, and determining solutions	**Low level:** Repeats and emphasizes encountered problems, such as how to attack and what to do if the attack fails. **Medium level:** Frames the core problem as “destroying the turret”, without considering efficiency factors such as allied unit combination and attack sequence. **High level:** Frames the problem as “locating the town center and destroying the turret”, and focuses on improving efficiency.
**Team Support:** Help and support for team peers	**The discussion is limited to resource exchange, unit formation, and turret attack.** **Example:** When a teammate transports units to the frontline. **Low level:** Refuses to provide transport ship assistance. **Medium level:** Sends transport ships to assist troop transportation only when requested by the teammate. **High level:** Takes the initiative to dispatch transport ships in advance to help the teammate transport troops.

**Table 3:** Illustrative examples of the draft of the observation scoring rubric.

Item Type	Behavioral Indicators	Scoring Method
Multiple-select Item	A. Decomposes the problem into “pathfinding—destroying external buildings.” B. Only chooses to destroy enemy buildings near the river	Award 2 points for full selection, 1 point for single selection, and 0 points for other cases. Score in this example: 1.
Frequency-count Item	A. Passively responds or expresses views ambiguously B. Expresses views coherently and clearly without explaining the rationale to peers C. Clearly states views and rationale to peers using explanatory language	Calculate the weighted mean of frequencies for indicators A, B, and C, then add 1 and round to an integer. Weights: −1, 0, 1 respectively. Theoretical scores: 0, 1, 2. Example calculation: round[((−1 × 3) + (0 × 4) + (1 × 2))/(3 + 4 + 2) + 1] = 1 point.
Single-select Item	A. Provides no support when peers ask for help B. Encounters no difficulty or provides support when peers ask for help C. Detects peers’ difficulty and proactively offers support	Score 0 for A; 1 for B; 2 for C. Score in this example: 0.

Note: For each item type, observers first identified the behavioral evidence from gameplay recordings, then selected the corresponding rubric options, and finally applied the scoring formulas to obtain proficiency-level scores (0, 1, or 2). See Supplementary Materials C for the complete scoring form.

**Table 4:** Coded transcript excerpt related to the Turret Destruction Task.

Question 1: Please Describe the Process You Used to Complete the Turret Destruction Task.
**Transcript:** At first, when I entered the city, I didn’t really know what to do. My teammate told me to find the town center, so I sent my infantry to scout. But they had only walked a few steps when they were killed by the turret. Then we decided we had to destroy the turret first. We produced units—rams and cavalry—and sent them all in at once, but every unit was killed. We wondered if we could bypass it as we did earlier, so we destroyed the small buildings around it, only to find it useless. There were trees behind the buildings blocking the way, so we still couldn’t get through. We had no choice but to figure out how to destroy the turret. I noticed the turret fired only one shell at a time, with a relatively slow trajectory. So we tried sending rams in three separate groups to attack, hoping to take it down. But the rams were too slow; they were all destroyed before getting close. Horse archers could run and shoot, but their arrows did almost no damage to the turret. Finally, my teammate suggested sending cavalry first to draw fire, then sending in the rams. That worked.
**Coding (Cause–Behavior):** (1) Searching for the town center—Sent infantry to scout. (2) Attacked by the turret—Decided to destroy the turret. (3) Massed attack resulted in total loss—Attempted to bypass the turret. (4) Bypass failed—Explored weaknesses of the turret. (5) Discovered the turret fired single rounds with slow speed and long trajectory—Sent rams to attack in three groups. (6) Rams were too slow and failed—Switched to fast horse archers. (7) Horse archers could evade shells but dealt almost no damage—Tried using cavalry to draw fire while rams attacked the turret.
**Question 2: What difficulties or problems did you experience with game difficulty or operations?**
**Transcript:** This stage was indeed a bit more difficult than the previous ones, but it was okay—not too hard. It was just about figuring out how to destroy the turret; careful thinking would solve it. But two things felt off. First, the turret’s splash radius was too large; you had to react very quickly. Even after we knew the correct strategy, we failed once because we reacted too slowly. Second, a minor issue: since we didn’t destroy the turrets on the city walls, sometimes cavalry that circled too widely would be killed by outer turrets or arrow towers.
**Coding:** (1) Difficulty was slightly greater but acceptable. (2) The turret’s splash damage radius was too large.
**Question 3: What flaws or areas for improvement do you see in the game design?**
**Transcript:** Could you slightly increase the movement speed of rams? They move too slowly, and every time we restarted, we spent a lot of time just traveling.
**Coding:** (1) Rams are too slow; suggest a moderate speed increase.
**Question 4: Why did you rate the observability of task performance as poor, and what suggestions?**
**Transcript:** The indicator is supposed to measure mistakes during collaboration. Theoretically, based on the written description alone, you should be able to observe who made the mistake. But the shell has such a large splash radius that you can get hit even if you’re careful. This is not a mistake but a design issue. It would be better if the shell’s damage radius were reduced. Also, the description of collaborative performance is too general; it should be more specific.
**Coding:** (1) Overly large turret splash radius caused excessively high error rates. (2) The behavioral description of collaborative performance was too general; recommend making it more specific.

**Table 5:** The correlation analysis between sub-facets of the pilot study (*N* = 36).

Sub-Facets	*Mean*	*SD*	1	2	3	4	5	6
1. Comprehension and Representation	17.47	5.62	1					
2. Communication and Exchange	15.69	4.97	0.41 *	1				
3. Planning	15.08	5.21	0.31	0.50 **	1			
4. Execution	15.53	4.82	0.26	0.35 *	0.54 **	1		
5. Team Responsibility	16.53	6.49	0.34 *	0.53 **	0.26	0.38 *	1	
6. Peer Relationship	10.56	3.27	0.24	0.45 **	0.35 *	0.28	0.59 **	1

Note: * *p* < 0.05; ** *p* < 0.01.

**Table 6:** Element quality analysis (*N* = 298).

Element	Item Difficulty *b*		Thresholds *d*_1_	
	*μ*	*σ*	*μ*	*σ*
Task Context	0.12	0.93	−0.60	0.63
Own Role	0.35	0.29	−2.40	0.33
Peer Roles	−0.02	0.67	−2.34	0.76
View Expression	−0.36	1.97	−1.56	0.47
Perspective-Taking	0.83	0.59	−1.83	0.41
View Negotiation	−0.21	1.05	−0.48	0.34
Goal Setting	0.35	0.36	−1.05	0.71
Problem Decomposition	0.62	0.02	−1.17	0.77
Resource Management	0.57	0.06	−1.76	0.80
Task Performance	0.37	0.31	−3.06	1.15
Coordination	−0.23	1.16	−0.79	0.25
Outcome Evaluation	−0.47	0.77	−1.76	1.53
Collective Awareness	−0.24	0.91	−0.95	0.93
Concentration	0.14	0.35	−0.81	0.45
Task Advancement	−0.18	0.58	−0.65	0.60
Behavioral Norms	−0.63	0.33	−1.00	0.28
Team Support	−0.31	0.20	−2.01	0.14

**Table 7:** Model fit of CFA (*N* = 298).

Fit Indicator	Criterion	Model 0 3-Dimension	Model 1 1-Dimension	Model 2 Higher-Order	Model 3 Bi-Factor
*χ* ^2^	-	5.61	51.57	5.61	-
*df*	-	6	9	6	-
*χ*^2^/*df*	<2	0.94	5.73	0.94	-
TLI	≥0.95	0.99	0.86	0.99	-
CFI	≥0.90	0.99	0.91	0.99	-
RMSEA	≤0.05	0.01	0.13	0.01	-
SRMR	≤0.08	0.02	0.05	0.02	-

Note: The bi-factor Model 3 did not converge.

**Table 8:** Correlation coefficients between CPS competency and criterion variables (*N* = 298).

Criterion Variables	0	1	2	3	4	5	6	7	8	9
0. CPS Competency		**0.54** ***	**0.48** ***	**0.10** ^+^	0.02	**0.21** ***	**0.11** ^+^	0.01	−0.09	
1. Peer–Collaboration	0.52 ***		**0.63** ***	0.07	0.03	**0.11** ^+^	−0.04	0.00	−0.07	
2. Peer–Problem-solving	0.48 ***	0.63 ***		0.04	0.03	0.06	0.03	0.01	−0.06	
3. Raven’s Reasoning	0.14 *	0.08	0.06		−0.05	0.04	−0.00	0.09	0.06	
4. Extraversion	0.00	0.03	0.03	−0.04		**0.20** ***	0.04	0.11	**0.42** ***	
5. Agreeableness	0.26 ***	**0.11 ^+^**	0.07	0.06	0.18 **		0.09	−0.17	−0.00	
6. Conscientiousness	0.09	−0.04	0.02	−0.01	0.04	0.09		**0.37** ***	**0.33** ***	
7. Emotional Stability	−0.00	0.01	0.01	0.09	0.12 *	−0.18 **	0.37 ***		**0.38** ***	
8. Openness	−0.05	−0.05	−0.05	0.08	0.42 ***	0.00	0.32 ***	0.38 ***		
9. Game Flow Experience	0.34 ***	0.04	0.08	0.15 *	−0.03	0.21 **	−0.02	−0.05	0.06	
10. RTS Game Experience	0.25 ***	0.08	0.08	0.18 **	0.02	0.07	−0.03	0.04	0.13 *	0.61 ***

Note: + *p* < 0.1; * *p* < 0.05; ** *p* < 0.01; *** *p* < 0.001. Bold for all *p* < 0.1, *p* < 0.05, *p* < 0.01, and *p* < 0.001.

**Table 9:** Tests of differences in CPS competency by sex, major, and game role.

Variables	Level	Mean	*SD*	*F*	*df*	*p*
Sex	Male	0.06	0.54	1.47	1	.23
	Female	−0.04	0.47			
Major	Liberal arts	−0.08	0.49	1.08	3	.36
	Science	−0.02	0.48			
	Engineering	0.08	0.56			
	Other	0.11	0.40			
Game Role	Cavalry	0.01	0.49	0.06	1	.80
	Siege weapon	−0.01	0.52			
Sex × Major				1.27	3	.28
Sex × Game Role				0.16	1	.69
Major × Game Role				2.34	3	.07
Three-way Interaction				0.63	3	.60

## Data Availability

The raw data are openly available in the Open Science Framework repository at: https://osf.io/7hpjd/overview?view_only=8425053067b24361a0d1e172f99bce0a (accessed on 13 May 2026).

## Supplementary Materials

The following supporting information can be downloaded at: https://www.mdpi.com/article/10.3390/jintelligence14090197/s1, Supplementary Materials A: Guo and Yang (2025)’s mappings between behavioral indicators and CPS competency levels; Supplementary Materials B: The completed task design of Collaborative Siege Task. Supplementary Materials C: The Observation Scoring Form for Collaborative Siege Task. Supplementary Materials D: The item parameters of Collaborative Siege Task.
