# Supplementary material for: A Theory-Driven Game-Based Assessment of Collaborative Problem-Solving
Source: J Intell. 2026 Sep 1;14(9):197. doi: 10.3390/jintelligence14090197 (PMC13608352; doi:10.3390/jintelligence14090197)
Supplement: Supplementary file 1 [file jintelligence-14-00197-s001.zip › jintelligence-4438092-supplementary.pdf]

**Supplementary Materials**

This appendix includes following four sections:

Supplementary Materials A: Guo and Yang (2025)'s mappings between behavioral indicators and CPS competency levels

Supplementary Materials B: The completed task design of Collaborative Siege Task.

Supplementary Materials C: The Observation Scoring Form for Collaborative Siege Task.

Supplementary Materials D: The item parameters of Collaborative Siege Task.

**Supplementary Materials:** Guo and Yang (2025)'s mappings between behavioral indicators and CPS competency levels

Table A1. The Mappings for Reaching Team Consensus

| Sub-facet | Element | Indicator | Low Level | Medium Level | High Level |
|---|---|---|---|---|---|
| **Comprehension and Representation:** The ability of individuals to recognize and internalize various information required for problem-solving based on their general cognitive abilities and past knowledge and experience. | Task Context | Degree of understanding of the task context | Able to mechanically memorize and describe information about the task context but unable to understand and apply it | Able to correctly understand and minimally utilize key elements of the task context to solve problems | Able to efficiently apply acquired key elements of the task context to solve problems |
| | Own Role | Degree of understanding of the role of one's own role in collaboration | Able to mechanically memorize and describe information about one's own role but unable to understand and apply it | Able to correctly understand and minimally utilize key elements of one's own role to solve problems | Able to efficiently apply acquired key elements of one's own role to solve problems |
| | Peer Roles | Degree of understanding of the role of peer roles in collaboration | Able to mechanically memorize and describe information about peer roles but unable to understand and apply it | Able to correctly understand and minimally utilize key elements of peer roles to solve problems | Able to efficiently apply acquired key elements of peer roles to solve problems |
| **Communication and Exchange:** The ability of collaborators to exchange opinions through communication. | View Expression | Expression of one's own viewpoints or opinions | Tends to respond passively, with fragmented and chaotic viewpoints | Able to express viewpoints coherently and clearly from one's own perspective without explaining the basis for the viewpoints to peers | Able to clearly state one's own viewpoints and basis to peers using explanatory language |
| | Perspective-Taking | Listening to, adopting or refuting others' viewpoints | Ignores or follows others' viewpoints and opinions without consideration | Able to adopt or refute others' viewpoints and opinions based on evidence | Able to integrate others' opinions into one's own thinking to propose new viewpoints |
| | View Negotiation | Mediating differences and conflicts among team members | Quarrels only to emphasize the correctness of one's own viewpoint, neither explaining reasons nor attempting to resolve conflicts | No disputes arise, or disputes can be mediated through compromise, concession or suspension | Able to resolve disputes through practice or proposing schemes to verify viewpoints |

Table A2. The Mappings for Organizing Team Actions

| Sub-facet | Element | Indicator | Low Level | Medium Level | High Level |
|---|---|---|---|---|---|
| **Planning:** The ability of individuals to formulate action steps and plans for the team to achieve team goals based on the joint problem space. | Goal Setting | Level of goal-setting according to the current team situation | No goals set or only macro and vague goals set | Able to set clear and specific goals without considering whether the goals meet the needs of the current situation | Able to set specific, proximal and constructive goals according to the current situation |
| | Problem Deconstruction | Level of analyzing problems, exploring ideas and determining solutions | Restates or describes problems in one's own words but fails to propose problem-solving methods | Able to identify necessary approaches to solve problems and propose suboptimal solutions | Able to analyze key factors for problem-solving and propose optimal solutions |
| | Resource Management | Level of understanding, sharing and utilizing resources possessed by team members | Unaware of peers' strengths and resources, and only considers one's own resources when formulating plans | Aware of strengths and resources possessed by team members, and able to minimally utilize team resources to formulate plans | Able to fully and rationally utilize team resources according to problem needs and resource characteristics to formulate plans |
| **Execution:** The ability of individuals to collaboratively solve problems through concrete actions. | Task performance | Efficiency and quality level in implementing action plans collaboratively | Unable to collaborate with peers to implement the plan immediately after formulation | Able to start collaborating with peers immediately after plan formulation but fail to properly execute the plan to achieve goals | Able to start collaborating with peers immediately after plan formulation and properly execute the plan to achieve goals |
| | Coordination | Degree of consistency in actions when collaborating with peers | Requires peers to adapt to one's own behavioral patterns to take action | Able to cooperate with peers in accordance with the plan | Able to proactively adapt to peers' behavioral patterns to cooperate |
| | Outcome Evaluation | Evaluation and reflection on execution results | Does not evaluate or reflect on experiences of success or failure | Only evaluates outcomes as success or failure or fails to correctly analyze the causes of success or failure | Able to correctly analyze the causes of success or failure and adjust actions adaptively |

Table A3. The Mappings for Maintaining Team Function

| Sub-facet | Element | Indicator | Low Level | Medium Level | High Level |
|---|---|---|---|---|---|
| **Team Responsibility:** The ability of individuals to take responsibility when solving problems as a team. | Collective Awareness | Awareness of thinking and acting from the team's perspective | Conducts activities independently and does not report progress to peers | Able to conduct activities with the team and report progress to peers | Able to proactively understand peers' activity progress and provide feedback to the team based on one's own situation |
| | Concentration | Degree of concentration in problem-solving | Unable to concentrate on problem-solving, easily distracted or off-topic | Able to concentrate on problem-solving but may be distracted or go off-topic due to peer influence | Able to concentrate highly on problem-solving and timely correct peers' distractions and off-topic behaviors |
| | Task Advancement | Level of promoting the team to solve problems together | Takes no proactive actions to advance the task | Able to promote problem-solving through one's own unilateral efforts | Able to jointly advance the process through interaction with peers |
| **Peer Relationship:** The ability of individuals to maintain harmonious relationships with peers when collaboratively solving problems. | Behavioral Norms | Commonly observed behavioral norms in social interactions | Interrupts, teases or orders others' speeches or actions | Respects others' contributions, speeches or actions | Praises or affirms others' key contributions, speeches or actions |
| | Team Support | Help and support for team peers | Does not support peers when they seek help | Able to provide support to peers when they seek help | Able to detect peers' difficulties and proactively provide support |

**Supplementary Materials B:** The completed task design of Collaborative Siege Task.

Before starting the assessment task, participants first need to learn and adapt to the game environment and operation mode of Age of Empires II (AOE2). To this end, this study adopts the built-in tutorial campaign William Wallace in AOE2 as the template, and develops tutorial tasks and demonstration videos that provide real-time voice and text instructions to help participants master the game rules. By learning these materials, participants can quickly grasp the basic operations in the AOE2 system, including map exploration, resource gathering, as well as military training and command.

As it shown in Figure B1, prior to rule tutoring, participants are first required to watch a pre-recorded demonstration video of the tutorial task. Watching the video enables participants to form a macro understanding and psychological preparation for the upcoming tutorial task, and also serves as procedural guidance when they subsequently experience the tutorial task in person. After viewing the video, participants officially enter the tutorial scenario and follow the real-time voice and text prompts to operate independently until they have mastered all essential skills required for the gamified assessment task. In general, the entire tutoring session takes approximately 15 minutes to complete.

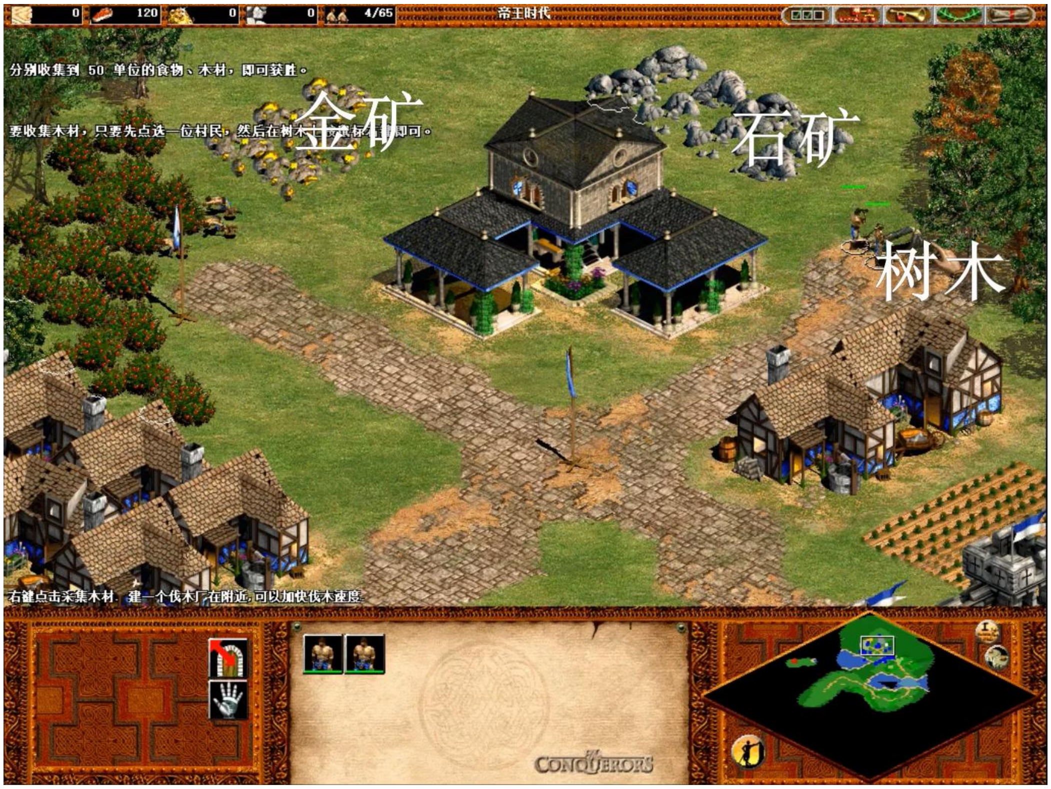


Figure B1. Screenshots of the demonstration video for the tutorial task

**Video and printed instructions for the cooperative siege mission**

**Cavalry Commander Video Instruction**

Hello! Thank you for participating in this experiment.

Next, I will briefly explain the tasks you need to complete. If you do not fully understand the instructions, you may replay the video or read the paper version.

In this task, you act as a cavalry commander, and your partner acts as a Siege Weapon Commander. Your joint mission is to communicate, cooperate in combat, and destroy the enemy's Town Center together in the shortest possible time.

At the start of the task, your view is fixed on the main base on the left side of the map. You control the blue Stable, Archery Range and Dock, while your partner controls the green Siege Workshop and Barracks.

In the Stable, you can produce Elite Scout Cavalry by consuming 100 units of Food. The Elite Scout Cavalry is a light and fast reconnaissance unit with a wide field of vision and moderate resistance to ranged attacks, yet it has low combat effectiveness.

You may also click the Dock to produce Transport Ships by consuming 100 units of Wood and Food, which can quickly transport your units across rivers.

Move your view to the upper-left corner, and you will see the logistics supply base shared by you and your partner. You own an orchard and a gold mine, while your partner owns a forest and a stone mine. You can click the olive branch icon in the upper-right corner of the screen to access the Market and deliver resources to your partner.

Pan your view to the right, and you will see four Watchtowers built by the red enemy along four pathways. The enemy's Town Center is likely located at the end of the unexplored roads.

In addition, your Archery Range is training a group of Horse archers specialized in ranged combat. These Horse archers will become available to you once you destroy any one of the enemy's Castles.

**Important Note:** You may have a maximum of only 3 Scout Cavalry or Horse archers at the same time. Therefore, you must communicate actively with your teammate and rely on coordinated combat to successfully destroy the enemy's Town Center.

You have 90 minutes to complete this task. Good luck!

## Cavalry Commander Paper Instruction

In this task, you are a cavalry commander, and your partner is a Siege Weapon Commander.

**Your mission:** Communicate and cooperate in combat to destroy the red enemy's Town Center as quickly as possible.

**Time Limit:** 90 minutes

**Notes:**

1. You must communicate and collaborate with your partner to complete the task.

2. You command all blue units; your partner commands all green units.

3. Please refer to the white text prompts in the bottom-left corner during the game.

4. At the start of the game, you can only train Scout Cavalry and Transport Ships at the Stable and Dock.

5. You may only train Horse Archers at the Archery Range (bottom-right corner) after destroying any enemy Castle.

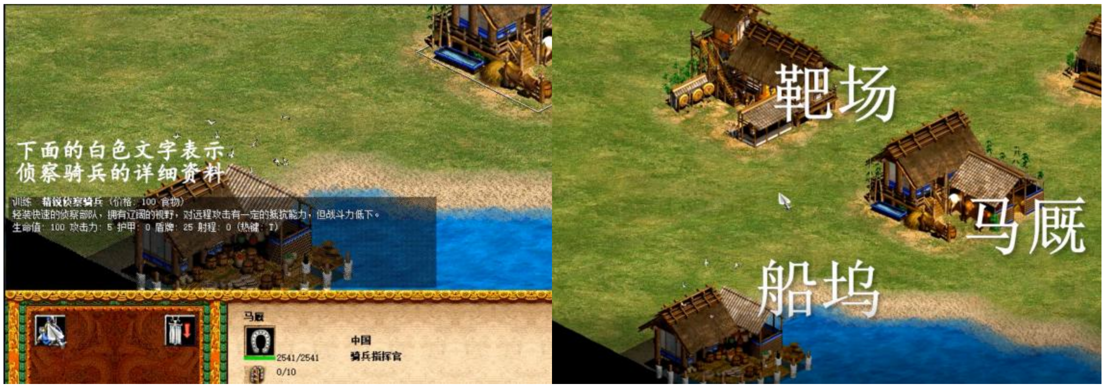


(Note：靶场：Archery Range；马厩：Stable；船坞：Dock)

6. Your logistics supply base is located above the Archery Range. Click the olive branch icon in the upper-right corner of the interface to send resources to your partner.

7. You may have a maximum of 3 Scout Cavalry or Horse archers at one time. Fallen units can be retrained at the Stable and other production buildings.

**Hint:** The enemy's Town Center is likely situated at the end of the roads with the four Watchtowers.

**Siege Weapon Commander Video Instruction**

Hello! Thank you for participating in this experiment.

Next, I will briefly explain the tasks you need to complete. If you do not fully understand the instructions, you may replay the video or read the paper version.

In this task, you act as a Siege Weapon Commander, and your partner acts as a cavalry commander. Your joint mission is to communicate, cooperate in combat, and destroy the enemy's Town Center together in the shortest possible time.

At the start of the task, your view is fixed on the main base on the left side of the map. You control the green Siege Workshop and Barracks, while your partner controls the blue Stable, Archery Range and Dock.

In the Siege Workshop, you can produce Heavy Battering Rams by consuming 100 units of Wood. The Heavy Siege Ram is a slow-moving siege unit specialized in destroying buildings. It has strong defense against arrow attacks but is vulnerable to melee strikes and blunt weapon attacks (e.g., cannonballs, battle axes).

Move your view to the upper-left corner, and you will see the logistics supply base shared by you and your partner. You own a forest and a stone mine, while your partner owns an orchard and a gold mine. You can click the olive branch icon in the upper-right corner of the screen to access the Market and deliver resources to your partner.

Pan your view to the right, and you will see four Watchtowers built by the red enemy along four pathways. The enemy's Town Center is likely located at the end of the unexplored roads.

In addition, your Barracks is training a group of Heavy Infantry specialized in melee combat. These infantry units will become available to you once you destroy any one of the enemy's Castles.

**Important Note:** You may have a maximum of only 3 Battering Rams or Heavy Infantry at the same time. Therefore, you must communicate actively with your teammate and rely on coordinated combat to successfully destroy the enemy's Town Center.

You have 90 minutes to complete this task. Good luck!

**Siege Weapon Commander Paper Instruction**

In this task, you are a Siege Weapon Commander, and your partner is a cavalry commander.

**Your mission:** Communicate and cooperate in combat to destroy the red enemy's Town Center as quickly as possible.

**Time Limit:** 90 minutes

**Notes:**

1. You must communicate and collaborate with your partner to complete the task.

2. You command all green units; your partner commands all blue units.

3. Please refer to the white text prompts in the bottom-left corner during the game.

4. At the start of the game, you can only train battering rams at the Siege Workshop.

5. You may only train Heavy Infantry at the Barracks (bottom-right corner) after destroying any enemy Castle.

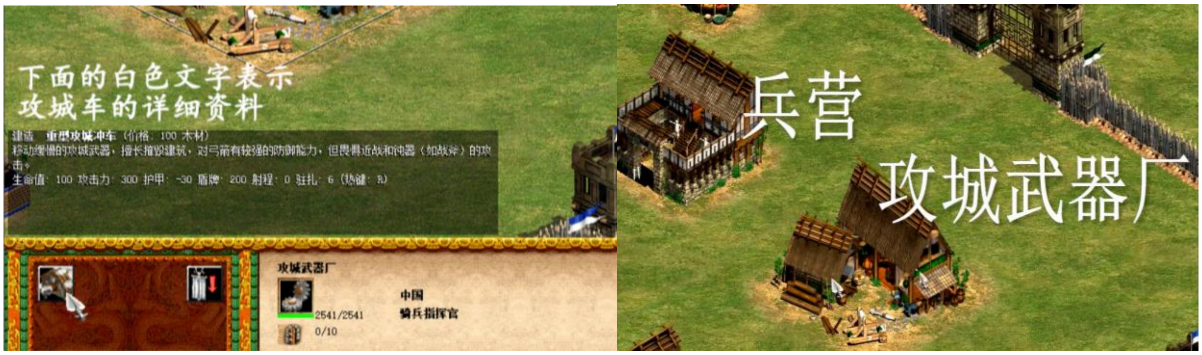

(Note：兵营：Barracks；攻城武器厂：Siege Workshop)

6. Your logistics supply base is located above the Barracks. Click the olive branch icon in the upper-right corner of the interface to send resources to your partner.

7. You may have a maximum of 3 Battering Rams or Heavy Infantry at one time. Fallen units can be replenished at the Siege Workshop and other production buildings.

**Hint:** The enemy's Town Center is likely situated at the end of the roads with the four Watchtowers.

**Overview of the Collaborative Siege Task**

This study designed a Collaborative Siege Task set against the backdrop of ancient warfare, tailored for two-person collaborative problem-solving. The Figure B2 presents the top-down map of the Collaborative Siege Task. As illustrated, the task involves three forces. Two collaborators form an allied force, respectively playing the role of the Cavalry Commander (Blue) and Siege Weapon Commander (Green) stationed on the left side of the map. The enemy force (Red) on the right side of the map is controlled by pre-set artificial intelligence, acting only in passive defense.

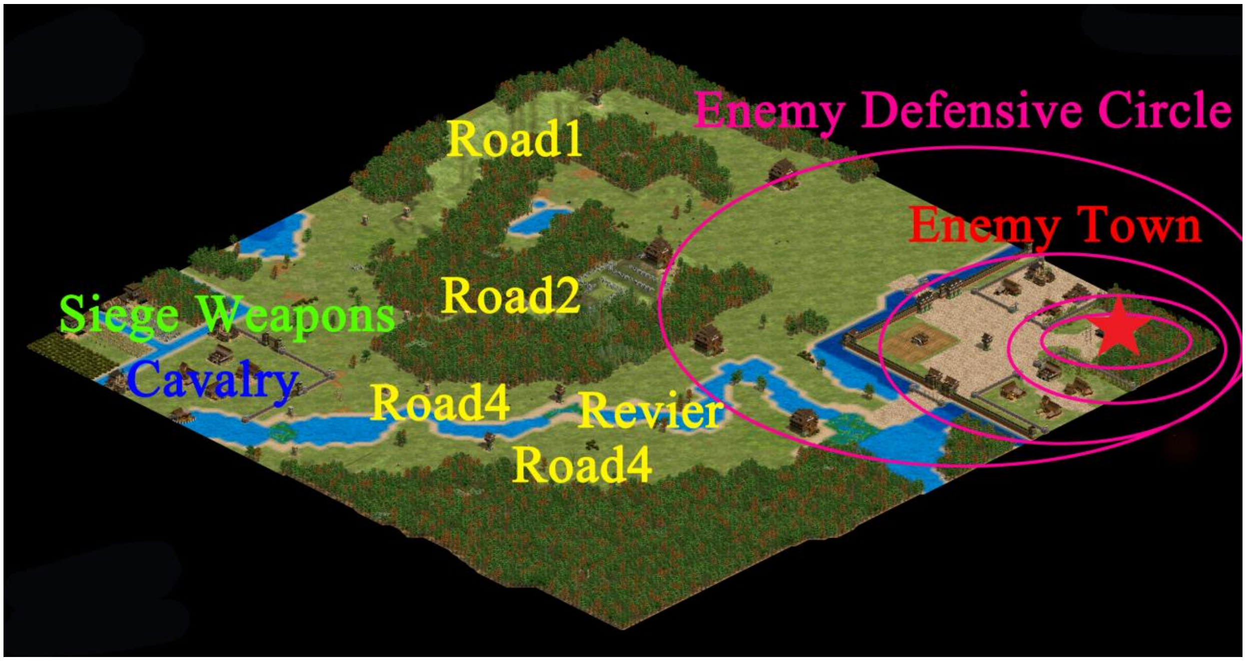

Figure B2. Task Map of the Gamified Assessment

At the start of the task, each member of the allied collaboration team owns an independent base for troop training and logistical supply. The enemy occupies a heavily fortified stronghold surrounded by a moat (marked by the pink defensive perimeter). The core task for the two collaborators is to communicate, engage in coordinated combat, and jointly destroy the enemy's Town Center (marked by the red five-pointed star) as quickly as possible.

Between the alliance and the enemy lies a large forest and a river. The alliance may launch offensives via four land routes through the forest and one river route. Meanwhile, the enemy has deployed various defensive structures along these routes for perimeter defense. Following the principle of task completion sequence and progressive difficulty, the task is divided into five phases or subtasks:

**Resource Preparation and Unit Training Task:** Watch and read the video and paper-based instructions, enter the game interface, recognize, collect and utilize resources within the allied base to train respective troops.

**Exploration and Path Selection Task**: Lacking battlefield intelligence, the alliance is unaware of the rebel stronghold's location. They need to dispatch units to explore the map, determine the advance route, break through the outermost defensive perimeter of the rebels, and clear pathways for the subsequent siege phase.

**Outer Gate Breaching Task**: Due to the moat, only two city gates are accessible for attack. The alliance needs to probe the firepower of both gates, select one to launch a concentrated assault, and break through the second defensive perimeter.

**Turret Destruction Task**: After breaking through the city gates, the rebels have built powerful turrets inside the inner city. The alliance must collaborate to destroy these turrets and breach the third defensive perimeter.

**Town Center Capture Task**: Following the destruction of the turrets, the remaining enemy main forces will hold firm at the fourth defensive perimeter in front of their headquarters. The alliance achieves victory by eliminating the enemy troops and destroying the Town Center.

Subsequently, this appendix elaborates on the detailed task design of the five phases and the problem-solving process experienced by the two collaborating parties respectively.

**Design of Task 1: Resource Preparation and Unit Training Task**

Before the start of the task, the two collaborators shall first watch the video and read the printed instructions separately and clarify that the current problem to be solved is to communicate, cooperate in combat, and jointly destroy the red enemy's Town Center as quickly as possible. The two collaborators then enter the task operation interface as shown in Figures B3 and B4 at the same time.

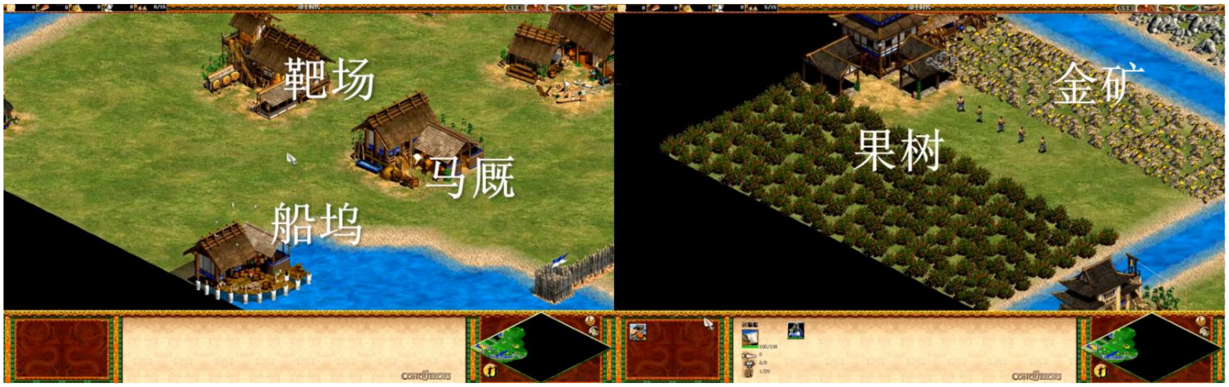

Figures B3. The view of Cavalry Commander

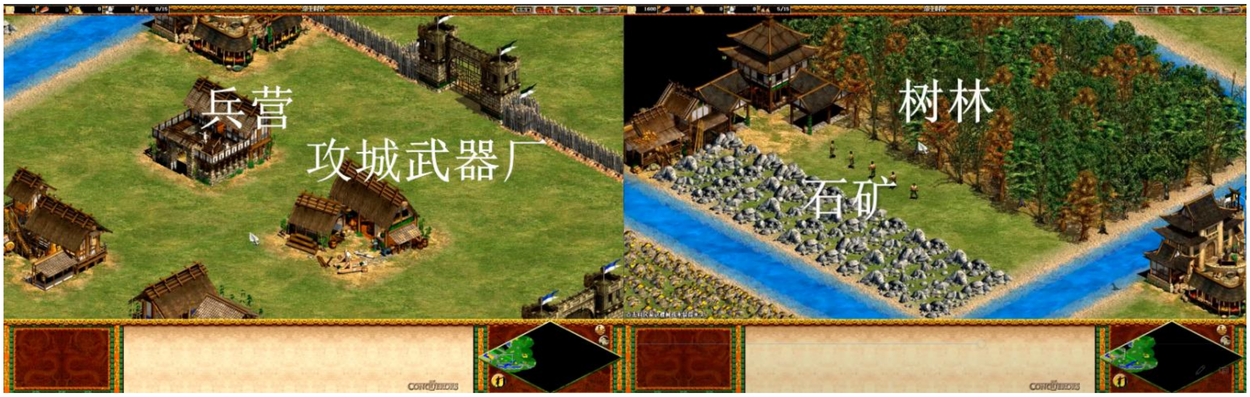


Figures B4. The view of Siege Weapon Commander

Table B1. Key elements owned and operated by the two collaborators in the Task 1.

| Role Identity | Type | Key Elements | Description |
|---|---|---|---|
| Cavalry Commander | Resources | Initial Resources | Wood, Food, Gold, and Stone all start at 0 |
| | | Orchard | Provides Food (essential); Food can be gifted to the partner |
| | | Gold Mine | Provides Gold (temporarily useless); Gold can be gifted to the partner |
| | Buildings | Stable | Building for training Scout Cavalry (initially available) |
| | | Dock | Building for training Transport Ships (initially available) |
| | Support | 5 Villagers | Each villager can gather 25 units of one resource at a time (initially available) |
| | | Transport Ship | Ship that quickly transports units (mainly Battering Rams) across rivers; requires 100 Food and 100 Wood to train |
| | Combat (Max. 3) | Scout Cavalry | Light, fast reconnaissance unit with wide vision, moderate resistance to ranged attacks, but low combat effectiveness; requires 100 Food to train |
| Siege Weapon Commander | Resources | Initial Resources | Wood, Food, Gold, and Stone all start at 0 |
| | | Forest | Provides Wood (essential); Wood can be gifted to the partner |
| | | Stone Mine | Provides Stone (temporarily useless); Stone can be gifted to the partner |
| | Buildings | Siege Workshop | Building for training Battering Rams (initially available) |
| | Support | 5 Villagers | Each villager can gather 25 units of one resource at a time (initially available) |
| | Combat (Max. 3) | Siege Ram | Slow siege unit specialized in destroying buildings, strong defense against arrows, but vulnerable to melee and blunt attacks; requires 100 Wood to train |

After entering the game, the two collaborators first view their currently available units and resources, and can then check the detailed information of these units by clicking on them. In addition, due to shared vision, the

collaborators can also see each other's units and resources, but cannot view the detailed data of each unit in the bottom-left corner or the function descriptions listed in Table B1.

Throughout the task, each commander may field a maximum of 3 combat units at the same time. Specifically, during the preparation phase, the Cavalry Commander can train Scout Cavalry and non-combat Transport Ships, while the Siege Weapon Commander can train Battering Rams.

The training methods for the three types of units are as follows:

**Siege Ram:** Villagers gather 100 units of Wood; select the Siege Workshop to train a Siege Ram.

**Scout Cavalry:** Villagers gather 100 units of Food; select the Stable to train a Scout Cavalry.

**Transport Ship (non-combat):** Villagers gather 100 units of Wood and 100 units of Food; select the Dock to train a Transport Ship. Notably, training a Transport Ship requires both Wood and Food. Food can be gathered by the Cavalry Commander himself, but Wood must be supplied by the Siege Weapon Commander.

After a brief adaptation, participants shall combine the above problem context with the experience acquired during the instruction and practice phases, and then conclude that to destroy the Town Center, they must first train military units. Accordingly, the practical problem in the preparation phase is transformed into how to train military units. The Figure B5 provides a further analysis of this problem of in the preparation phase. Its essence is to establish a circular system of resource gathering–resource storage–resource consumption, laying a material foundation for the continuous training of military units in the subsequent phases.

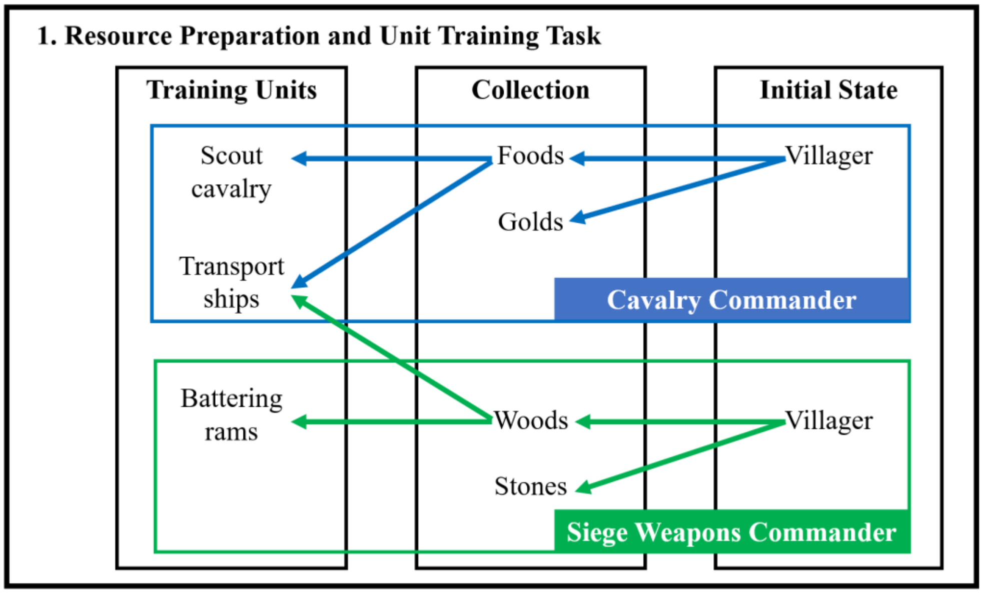


Figures B5. The task decomposition of Task 1.

The main purpose of designing the preparation stage is to help participants become familiar with and adapt to the testing environment. Therefore, the collaborative task at this stage is actually very simple, and participants can pass it only by slightly transferring the knowledge they have learned in teaching and training. In the most extreme case, the two collaborators can even enter the exploration stage directly by gathering resources and training units on their own without any communication. Of course, for the needs of CPS competency assessment, several factors affecting task completion efficiency are also designed in the preparation stage, among which the three most important factors are:

First, the allocation ratio of the 5 villagers when gathering resources. For the preparation stage, training one combat unit consumes 100 units of Food (or Wood), with no demand for Gold (or Stone). However, for participants, the resources available for the 5 villagers to gather include both Food and Gold (or Wood and Stone) at the same time. This mismatch between supply and demand will cause efficiency problems. Since each villager can only gather 25 units of resources at a time, at least 4 villagers are needed to collect 100 units of resources at one time to meet the resource demand for training a combat unit. Therefore, if participants dispatch more than 4 villagers to gather the necessary Food (or Wood) resources, it indicates that they can "take targeted measures" to improve efficiency. On the contrary, if participants only send 1 villager to gather Food (or Wood), although they can slowly save up 100 units, it will obviously reduce efficiency. In addition, villagers' resource gathering is a round-trip process from the Town Center to the resource location. Since the resources are distributed over a relatively wide area, the distance between the Town Center and the resource location directly affects the speed of resource gathering, making it more important to gather resources according to the proximity principle.

Second, the construction and use of Transport Ships. Although the preparation stage does not mandate the construction of Transport Ships to pass the stage, the existence of Transport Ships can greatly improve the efficiency of task completion. Although Transport Ships are trained by the Cavalry Commander, their high-speed advantage brings no practical benefit to the Cavalry Commander himself. The two collaborators need to realize through communication that the greatest function of Transport Ships is to carry Battering Rams to compensate for their slow movement speed, thereby improving efficiency. In addition, training Transport Ships requires the support of two types of resources from both collaborators, which also provides a topic for communication between the two parties.

Third, resource exchange. Although the two collaborators can freely exchange their own resources, in the preparation stage, only the supply of Wood resources from the Siege Weapon Commander to the Cavalry Commander for training Transport Ships is practically meaningful. Except for this situation, any other resource exchange will lead to delays in one's own development and thus reduce the efficiency of task completion.

From the perspective of completing the final task, the theoretical optimal solution for the preparation stage is as follows:(1) After recognizing the specific resources needed to train one's own units (100 units of Wood or Food), command more than 4 villagers to gather the specific resources nearby (4 villagers can exactly gather 100 units of resources at a time), and train 3 military units (Scout Cavalry or Battering Rams).(2) Understand the strengths of the units under one's own command (see Table B1 for details), and understand the strengths and resource consumption of the units under the partner's command through communication with the partner, especially recognizing the speed advantage of Transport Ships, and that training Transport Ships consumes both Wood and Food, while Wood resources can only be gathered and supplied by the Siege Weapon Commander.(3) Recognize that slow-moving Battering Rams can move quickly with the help of Transport Ships; the Siege Weapon Commander provides more than 100 units of Wood to the Cavalry Commander, so as to train at least 1 Transport Ship.

From the perspective of smoothly entering the next stage, the theoretical minimum requirements for the preparation stage are as follows:(1) Although they may not recognize the specific resources needed to train military units (100 units of Wood or Food), they still command more than 1 villager to gather the specific resources.(2) Although they do not understand the characteristics of the units under their own and their partner's command, they have trained at least 1 military unit (Scout Cavalry or Siege Ram).

On the basis of the above text, Table B2 further analyzes the relationship between task design and CPS competency assessment in the preparation stage in combination with the theoretical requirements of the CPS competency assessment framework. These analyses will contribute to the subsequent cognitive task analysis and scoring model design.

Table B2. Relationship between task design and CPS competency assessment in Task 1

| Sub-facet | Element | Relationship with Task Design |
|---|---|---|
| Comprehension and Representation | Task Context | **Low:** Be overwhelmed or mechanically apply tutorials from the practice phase.<br>**Medium:** Recognize the need to gather resources to train units.<br>**High:** Recognize the different priorities of gathering various resources. |
| | Own Role | **Low:** Mechanically apply the role identity from the practice phase.<br>**Medium:** Understand the characteristics of one's own resources and units, and meet or exceed the theoretical minimum requirements.<br>**High:** Based on the medium level, analyze the uniqueness of one's own role (see Table B1) and act in line with the theoretical optimal solution. |
| | Peer Role | **Low:** Mechanically apply the peer's role identity from the practice phase.<br>**Medium:** Understand the characteristics of the peer's resources and units, and meet or exceed the theoretical minimum requirements.<br>**High:** Based on the medium level, analyze the uniqueness of the peer's role (see Table B1) and act in line with the theoretical optimal solution. |
| Communication and Exchange | View Expression | Topics are limited to resource types and unit characteristics.<br>**Example:** Requesting the partner to provide Wood to build ships.<br>**Low:** Do not initiate proposals; only passively agree if the partner proposes first.<br>**Medium:** "Give me 100 Wood."<br>**High:** "Building a ship requires 100 Wood. Could you send it to me?" |
| | Perspective-Taking | Topics are limited to resource types and unit characteristics.<br>**Example:** The partner proposes to receive all of one's Wood resources.<br>**Low:** Pretend not to hear or agree directly.<br>**Medium:** "What do you need Wood for? All of it?"<br>**High:** "Are you going to build a ship? How much Wood does it need?" |
| | View Negotiation | Topics are limited to resource types and unit characteristics.<br>Example: Being refused by the partner for insufficient Wood when requesting support.<br>**Low:** "I need it urgently. Just give it to me first."<br>**Medium:** "Then send me some extra when you are done using it."<br>**High:** "Didn't you say your Siege Ram is slow? I'll use this Wood to build a ship and carry your unit to speed it up." |
| Planning | Goal Setting | **Low:** Set no goal or only aim to complete the task quickly.<br>**Medium:** Set the goal to train units to search for the enemy's Town Center.<br>**High:** Set the goal to prepare logistics, stockpile resources, and train more units. |
| | Problem Deconstruction | **Low:** Restate or emphasize one's own problems (e.g., unable to train units, insufficient resources).<br>**Medium:** Deconstruct the problem as "villagers gather resources → consume resources to train units" but ignore efficiency (e.g., assign only 1 villager to gather Wood, do not build Transport Ships).<br>**High:** Deconstruct the problem as "villagers gather resources → consume resources to train units" and pursue efficiency (e.g., assign 4 villagers to gather Wood nearby, build Transport Ships). |
| | Resource Management | **Low:** Only consider one's own resources when planning (i.e., no plan to build Transport Ships).<br>**Medium:** Plan to use team resources to build Transport Ships but do not coordinate with Battering Rams.<br>**High:** Plan to use team resources to build Transport Ships and load Battering Rams to improve their speed. |
| Execution | Task Performance | **Low:** No plan to build Transport Ships, or the plan is rejected/shelved.<br>**Medium:** Build Transport Ships by exchanging both necessary and unnecessary resources.<br>**High:** Build Transport Ships by exchanging only necessary Wood resources. |

| | | |
|---|---|---|
| | Coordination | **Low:** No plan to build Transport Ships, or the plan requires the partner to wait.<br>**Medium:** Execute the plan to build Transport Ships together with the partner.<br>**High:** Anticipate the partner's needs (e.g., resource demands) and cooperate when building ships. |
| | Outcome Evaluation | When the system prompts insufficient resources to train units:<br>**Low:** Ignore the prompt and keep clicking the train button.<br>**Medium:** Stop clicking and try to find a solution but fail.<br>**High:** Stop clicking and successfully find a solution. |
| Team Responsibility | Collective Awareness | **Low:** Act independently without communicating with the partner.<br>**Medium:** Report one's own progress to the partner but ignore the partner's activities.<br>**High:** Monitor the partner's progress and give feedback based on one's own situation. |
| | Concentration | **Low:** Unable to focus on problem-solving; easily distracted or off-topic.<br>**Medium:** Do not initiate off-topic talks but may be distracted by the partner.<br>**High:** Stay focused and timely correct the partner's off-topic behaviors. |
| | Task Advancement | Topics are limited to resource types and unit characteristics.<br>**Example:** Exploring the solution direction.<br>**Low:** Take no action to explore the solution.<br>**Medium:** Analyze or attempt operations alone to explore the solution.<br>**High:** Collaboratively explore the solution through communication with the partner. |
| Peer Relationship | Behavioral Norms | Topics are limited to resource types and unit characteristics.<br>**Low:** Interrupt, tease, or order the partner's speech or actions.<br>**Medium:** Respect the partner's contributions, speech, and actions.<br>**High:** Praise or affirm the partner's key contributions, speech, and actions. |
| | Team Support | Topics are limited to resource types and unit characteristics.<br>**Example:** The partner asks for help about training units.<br>**Low:** "It's easy. Figure it out yourself."<br>**Medium:** "Click the Stable first, then the cavalry icon at the bottom left."<br>**High:** (Proactively notice the problem) "You don't know how to train units yet, right? First click the Stable, then the cavalry icon at the bottom left." |

## Design of Task 2: Exploration and Path Selection Task

In the exploration phase, the two collaborators need to command their troops to leave the allied base and locate the enemy's Town Center by exploring the map. Figure B6 shows a comparison between the map vision the allied forces can observe at the beginning of the exploration phase and the full map (black indicates unknown areas).

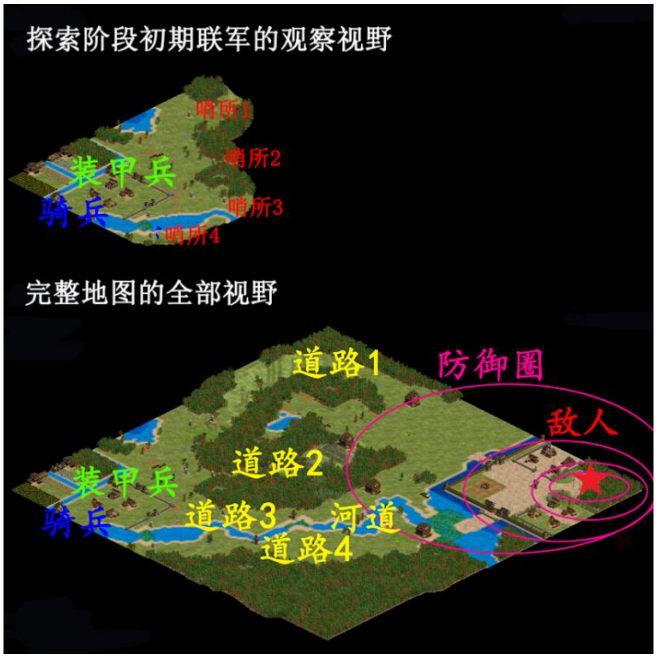


Figure B6. Comparison of the Allied Forces' Observation Field and the Full Map at the Beginning of the Exploration Phase

According to the hint in the instructions, "The enemy's Town Center is likely situated at the end of the roads with the four Watchtowers." The allied forces need to dispatch units to explore along four land routes and one river route. At the end of the roads, they will discover the enemy's town surrounded by a moat. Along the four routes shown in Figure B6, the enemy has deployed different defensive structures at intervals to counter the allied offensive. From left to right, these are Outposts, Watchtowers, and Castles. The characteristics of these defensive structures and their unit-level counter relationships with the allied forces are listed in Table B3. Additionally, Table B4 presents the geographical features of each route. Note that since the river runs between Route 3 and Route 4, the Watchtowers and Castles on both routes can attack passing ships within their range.

Table B3. Unit-Level Counter Relationships Between the Allied Forces and Enemy Troops in the Exploration Phase

| **Allied Forces** | **Enemy** | | |
|---|---|---|---|
| | Watchtower (No attack capability, provides vision only) | Guard Tower (Ranged single arrow) | Castle (Ranged multiple arrows) |
| **Siege Ram** (Melee) | Battering Rams can rapidly destroy Watchtowers. | Battering Rams can rapidly destroy Guard Towers, while Guard Towers can hardly damage Battering Rams. | Battering Rams can deal high damage to Castles; however, sturdy Castles can withstand their attacks for a while, and their multiple ranged arrows can heavily damage Battering Rams before they close in. |
| **Scout Cavalry** (Melee) | Scout Cavalry can slowly destroy Watchtowers. | Scout Cavalry can hardly damage Guard Towers in a head-on attack and will be quickly eliminated, but they can dodge ranged arrows with their fast movement speed. | Scout Cavalry can hardly damage Castles in a head-on attack and will be quickly eliminated, but they can dodge the multiple ranged arrows with their fast movement speed. |
| **Transport Ship** (No attack capability) | Cannot damage each other. | Transport Ships will be quickly eliminated by Guard Towers, but they can dodge the ranged arrows with their fast movement speed. | Transport Ships will be quickly eliminated by Castles, but they can dodge the ranged arrows with their fast movement speed. |

Table B4. Comparison of Geographical Conditions of the Five Routes

| Route | Distance to Enemy Town | Terrain Features | Notes |
|---|---|---|---|
| Route 1 | Far | Hilly area with open undulating terrain | None |
| Route 2 | Near | Mountain forest path, narrow and rugged terrain | None |
| Route 3 | Moderate | Riverside plain, flat and narrow terrain | None |
| Route 4 | Moderate | Riverside plain, flat and open terrain | None |
| River Route | Moderate | River waterway, flat and narrow terrain | The river is simultaneously blockaded by the defensive structures of Route 3 and Route 4. |

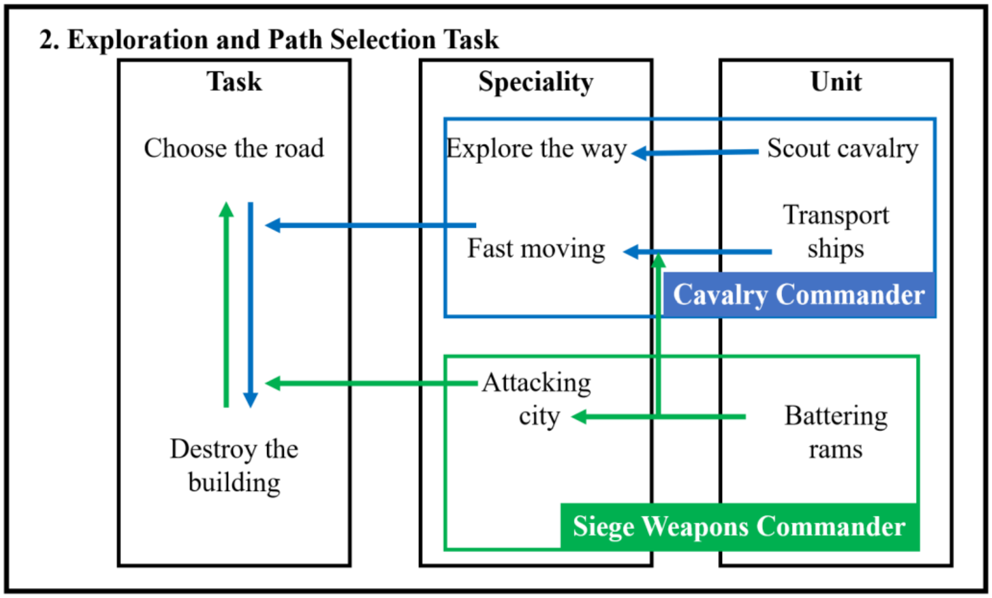


Figures B7. The task decomposition of Task 2.

Figure B7 further deconstructs the problem of "how to explore the map" in the exploration phase. The main task that the allied forces need to complete at this stage is to select a route to attack the town and dispatch troops to destroy the defensive structures the enemy has placed along the way, thereby reaching the moat on the outskirts of the enemy town. Compared with the preparation phase, the task difficulty and the necessity of collaborative problem-solving in the exploration phase have significantly increased, reaching a moderate level. The primary purpose of designing the exploration phase is to focus on individuals' behavioral performance when task difficulty and collaboration necessity are both relatively moderate. For the needs of CPS competency assessment, several factors affecting task completion efficiency have been designed in the exploration phase, three of which are the most important:

First, the utilization of unit characteristics. To meet the overall task requirement (i.e., destroy the Town Center in the shortest possible time within 90 minutes), the two collaborators need to work closely together, leveraging strengths and avoiding weaknesses based on an understanding of unit characteristics. Simply put, map exploration requires efficient use of Scout Cavalry's "fast movement speed and wide field of vision" advantage, while avoiding using the "slow-moving, narrow-vision" Battering Rams for scouting. Conversely, destroying defensive structures requires relying on the Siege Ram's expertise in "destroying buildings," and avoiding sending "low combat effectiveness" Scout Cavalry to attack. If the collaborators fail to effectively

utilize the strengths of their respective units, they might still barely pass at the cost of significant time and losses. However, even if they forcibly enter the next phase, they will inevitably fail to achieve the task goal due to insufficient time or excessive losses.

Second, the choice of attack route. Although the defensive structures on the four routes are identical, as shown in Table B4, the five routes differ significantly in geographical conditions (distance and terrain). First, to minimize time wasted on the road, Route 1, the farthest and with undulating terrain, should obviously be excluded. Second, although Route 2 is the closest, it is a narrow and rugged mountain forest path, clearly not the best choice. Route 3, Route 4, and the River Route have moderate distances and flat terrain. Considering terrain openness alone, Route 4 undoubtedly stands out. However, due to the slow movement speed of units like Battering Rams, compared to terrain openness and the time spent destroying defensive structures on the two routes, the River Route, which can quickly transport troops, clearly has a competitive advantage. In summary, choosing the River Route and simultaneously destroying the defensive structures on Route 3 and Route 4 is undoubtedly the optimal solution for the allied forces.

Third, the use of Transport Ships. Transport Ships can quickly transport troops, and their efficient use can significantly reduce the time wasted due to the slow movement speed of Battering Rams. Beyond whether or not Transport Ships are used, the collaborators' choice of embarkation point and landing location also affects task completion efficiency. For example, the Cavalry Commander might choose to dock the Transport Ship far from the Battering Rams instead of as close as possible, thereby reducing efficiency.

**From the perspective of completing the final task, the theoretical optimal solution for the exploration phase is as follows:**

(1) Both collaborators recognize that Scout Cavalry are fast, have a wide field of vision, and are adept at dodging arrows. Considering the placement of the Dock, they negotiate and prioritize sending one Scout Cavalry along the River Route to simultaneously explore Route 3 and Route 4 (or split the Scout Cavalry into three groups to explore simultaneously). When encountering enemy defensive structures, they avoid direct confrontation, using their speed advantage to evade attacks and continue scouting. Scout Cavalry are immediately replenished after being killed.

(2) Both collaborators recognize that Battering Rams are adept at attacking buildings but are slow. They negotiate to move all three Battering Rams together to destroy the closest Outposts to the allied base while the Scout Cavalry scouts. Battering Rams are immediately replenished after being killed and transported to the front line by Transport Ships from the nearest location.

(3) In the early stage, Transport Ships can also be sent to explore the river. However, if a Transport Ship is quickly sunk by enemy defensive structures, they stop forcing exploration and only operate outside the range of enemy buildings until those defensive structures are destroyed. Transport Ships are immediately replenished after being sunk.

(4) After initially obtaining map location information, choose the River Route as the main attack direction. Leveraging the speed advantage of Transport Ships, the Cavalry Commander dispatches Transport Ships to quickly transport three Battering Rams to a landing location outside the range of enemy defensive structures. Then, the Siege Weapon Commander commands the three Battering Rams to attack together, sequentially destroying the "Guard Towers" and "Castles" on Route 3 and Route 4 to ensure Transport Ships can travel unhindered on the river.

**From the perspective of smoothly entering the next phase, the theoretical minimum requirements for the exploration phase are as follows:**

(1) Randomly choose a route to advance, destroying or using speed advantage to forcibly bypass the defensive structures along the way.

(2) At least two or more Battering Rams are needed to attack together to have a chance of destroying a Castle.

Based on the above text, Table B5 further analyzes the relationship between task design and CPS competency assessment in the exploration phase, combining it with the theoretical requirements of the CPS competency assessment framework. These analyses will contribute to the subsequent cognitive task analysis and scoring model design.

Table B5. Relationship between Task Design and CPS Competency Assessment in the Exploration Phase

| Sub-facet | Element | Relationship with Task Design |
|---|---|---|
| Comprehension and Representation | Task Context | **Low:** Be overwhelmed or mechanically apply the tutorials from the practice phase.<br>**Medium:** Recognize the need to dispatch troops to explore the map to find the enemy's base.<br>**High:** Based on the medium level, prioritize exploration along the vicinity of the river. |
| | Own Role | **Cavalry Commander:**<br>**Low:** Thinks Scout Cavalry are mainly used to attack enemy buildings.<br>**Medium:** Thinks Scout Cavalry are mainly used for exploring routes and should retreat when encountering enemies.<br>**High:** Thinks Scout Cavalry are mainly for exploring routes, and when encountering enemies, should use numerical and speed advantages to evade attacks and continue deep exploration behind enemy lines.<br>**Siege Weapon Commander:**<br>**Low:** Thinks Battering Rams are mainly used for exploring routes.<br>**Medium:** Thinks Battering Rams are mainly used to destroy enemy buildings, but does not realize that all three Battering Rams should be assembled for a joint attack when assaulting.<br>**High:** Thinks Battering Rams are mainly used to destroy enemy buildings, and realizes that all three Battering Rams should be assembled for a joint attack when assaulting. |
| | Peer Role | **Cavalry Commander:**<br>**Low:** Only knows the peer can train Battering Rams but lacks in-depth understanding.<br>**Medium:** Recognizes that Battering Rams are good at siege but slow and not suitable for scouting.<br>**High:** Based on the medium level, gives the peer correct action suggestions, such as concentrating forces for a joint attack.<br>**Siege Weapon Commander:**<br>**Low:** Only knows the peer can train Scout Cavalry but lacks in-depth understanding.<br>**Medium:** Recognizes that Scout Cavalry are good at scouting but have low attack power and are not suitable for siege. |

| | | |
|---|---|---|
| | | **High:** Based on the medium level, gives the peer correct action suggestions, such as using speed advantage to force deep reconnaissance behind enemy lines. |
| Communication and Exchange | View Expression | Topics are limited to route selection and building destruction.<br>**Example:** Hoping to clear the river route together with the peer.<br>**Low:** Do not initiate proposals; only passively agree if the peer proposes first.<br>**Medium:** “Let’s attack along the river first.”<br>**High:** “After clearing the river route, we can take the ships. Let’s attack the river first.” |
| | Perspective-Taking | Topics are limited to route selection and building destruction.<br>**Example:** The peer proposes prioritizing an attack on Route 1.<br>**Low:** Pretend not to hear or agree directly.<br>**Medium:** “Route 1 is the farthest from our base. Shouldn’t we choose a closer one first?”<br>**High:** “I suggest sending cavalry to scout three routes simultaneously first, then make a decision.” |
| | View Negotiation | Topics are limited to route selection and building destruction.<br>**Example:** The peer demands destroying all Castles before attacking the town.<br>**Low:** “Why attack Castles? We should attack the town first.”<br>**Medium:** “Fine, whatever you say.”<br>**High:** “Our mission requires finding the Town Center, not destroying all Castles. We’ve already unlocked hidden troops; we can attack the town now. No need to waste extra time.” |
| Planning | Goal Setting | **Low:** Set no goal or only aim to complete the task quickly.<br>**Medium:** Set the goal to destroy all enemy buildings outside the town.<br>**High:** Set the goal to find and clear a path to the enemy town. |

| | | |
|---|---|---|
| | Problem Deconstruction | **Low:** Restate or emphasize one's own problems (e.g., where is the enemy, which path to take).<br>**Medium:** Able to deconstruct the problem as "scout – destroy buildings outside the town," but ignore efficiency (e.g., choose a farther route, destroy all buildings outside the town).<br>**High:** Able to deconstruct the problem as "scout – clear a fast attack route," and pay attention to efficiency (e.g., only destroy enemy buildings near the river route). |
| | Resource Management | **Low:** Only consider one's own resources when planning.<br>**Medium:** Proposes joint action when planning but fails to provide a coordination plan that leverages both sides' strengths (see theoretical optimal solution).<br>**High:** Proposes joint action when planning and provides a coordination plan that leverages both sides' strengths (see theoretical optimal solution). |
| Execution | Task Performance | **Low:** No plan formulated, or actual actions during execution do not match the plan.<br>**Medium:** Can generally follow the plan, but errors or omissions occur during execution.<br>**High:** Can follow the plan, and no errors or omissions occur during execution. |
| | Coordination | **Low:** Transport Ships not used, or Battering Rams need to actively approach to board.<br>**Medium:** Can board the Battering Rams onto Transport Ships at the agreed-upon location.<br>**High:** Can actively move the Transport Ship closer to the location of the Battering Rams to facilitate boarding. |
| | Outcome Evaluation | After one's own units are killed by enemy attacks:<br>**Low:** Continue attacking according to the original action pattern.<br>**Medium:** Try to analyze the cause of death and adjust actions, but without success.<br>**High:** Try to analyze the cause of death and adjust actions, successfully scouting or destroying enemy structures. |
| Team Responsibility | Collective Awareness | **Low:** Conduct activities independently without communicating with the peer.<br>**Medium:** Able to report one's own progress to the peer but ignore the peer's activities.<br>**High:** Able to monitor the peer's progress and give feedback based on one's own situation. |

| | | |
|---|---|---|
| | Concentration | **Low:** Unable to focus on problem-solving; easily distracted or off-topic.<br>**Medium:** Do not initiate off-topic talks but may be distracted by the peer.<br>**High:** Do not initiate off-topic talks and can timely correct the peer's off-topic behaviors. |
| | Task Advancement | Topics are limited to route selection and building destruction.<br>**Example:** Exploring the solution direction.<br>**Low:** Take no action to explore the solution.<br>**Medium:** Analyze or attempt operations alone to explore the solution.<br>**High:** Collaboratively explore the solution through communication with the peer. |
| Peer Relationship | Behavioral Norms | Topics are limited to route selection and building destruction.<br>**Low:** Interrupt, tease, or order the peer's speech or actions.<br>**Medium:** Respect the peer's contributions, speech, and actions.<br>**High:** Praise or affirm the peer's key contributions, speech, and actions. |
| | Team Support | Topics are limited to route selection and building destruction.<br>**Example:** The peer consults about the slow movement speed of Battering Rams and asks for a way to speed them up.<br>**Low:** "I don't know, just crawl slowly."<br>**Medium:** "You can use my Transport Ship, it'll be faster."<br>**High:** (Proactively notices the problem) "Take my ship to the front line, otherwise you'll be too slow." |

## Design of Task 3: Outer Gate Breaching Task

With the Castles destroyed, the allied forces gain access to new hidden units: Heavy Infantry and Horse Archers. The Heavy Infantry, trainable by the Siege Weapon Commander at the Barracks, is a heavy, slow-moving infantry unit specialized in melee combat. The Mounted Archer, trainable by the Cavalry Commander at the Archery Range, is a light, fast-moving cavalry unit that can attack with long-range arrows, and is the alliance's only ranged unit. Neither unit possesses any resistance to ranged attacks.

**The training methods for the two new units are as follows:**

**Heavy Infantry:** Villagers gather 100 units of Wood and 100 units of Gold; select the Barracks to train a Heavy Infantry.

**Mounted Archer:** Villagers gather 100 units of Food and 100 units of Stone; select the Archery Range to train a Mounted Archer.

It is important to note that training these hidden units requires not only resources that the commander can gather themselves (Wood and Food) but also resources that only their partner can gather (Gold and Stone). Therefore, the training of hidden units can only be achieved with the partner's assistance.

Having successfully cleared one of the routes during the Exploration phase, the allied forces arrive outside the moat surrounding the red enemy's town. As shown in Figure B8, due to the moat, the alliance can only enter the town through Gate 1 or Gate 2. The outer defenses of both gates are identical, each guarded by 5 Throw Axemen capable of short-range axe throwing. However, the defenses inside the gates differ significantly. Gate 1 is defended by 6 Enemy's Turrets, while Gate 2 has only 4 Arrow Towers. Table B6 shows the unit-level counter relationships between the allied forces and the enemy defenses at the gates. In terms of defensive power, the Enemy's Turrets are devastating, making Gate 1 nearly impossible to breach. The alliance needs to realize this and then attack the relatively weaker Gate 2. This Outer Gate Breaching Task is essentially the stage of breaking through the defenses at Gate 2 to enter the town's interior.

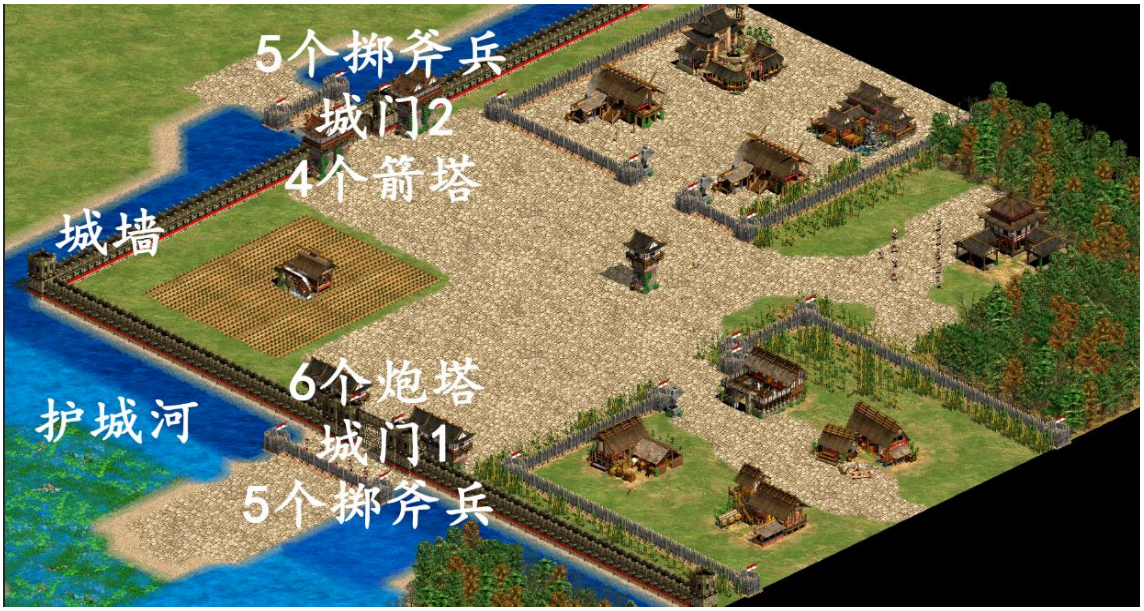


Figure B8. Outer defense map of the red enemy town during the Outer Gate Breaching Task.

As can be seen from Table B6, when attacking Gate 2, only the Siege Ram can withstand the attacks of four Arrow Towers simultaneously. However, the Siege Ram will be quickly destroyed by the Throw Axemen once it gets close to the city wall. Only the Mounted Archer can eliminate the Throw Axemen using its range advantage, but the Mounted Archer would be killed by the Arrow Towers before it could attack. Figure B9 further deconstructs the problem of "how to breach Gate 2" during the siege stage. The collaborators need to understand the enemy's attack priority and the importance of range: (1) The enemy will continuously attack the first allied unit that enters its range until that unit is destroyed or leaves the range. (2) The range of the Arrow Towers is greater than that of the Mounted Archer, which is greater than that of the Throw Axemen. Simply put, the alliance can first have the Battering Rams stop near the river to continuously draw fire from the Arrow Towers. Then, they can have the Horse Archers use their range advantage to eliminate the Throw Axemen from outside the Throw Axemen's range. Finally, the Battering Rams can attack and destroy the city wall and the four Arrow Towers.

Table B6. Unit-Level Counter Relationships Between the Allied Forces and Enemy Defenses in the Outer Gate Breaching Task

| **Allied Forces** | **Enemy** | | |
|---|---|---|---|
| | **Throw Axeman** (Short-range thrown axe) | **Arrow Tower** (Long-range single arrow) | **Cannon Tower** (Long-range single cannonball) |
| **Siege Ram** (Melee) | Siege Ram can barely damage Throw Axemen; Throw Axemen can quickly destroy Battering Rams. | Siege Ram can quickly destroy Arrow Towers, while Arrow Towers can hardly damage Battering Rams. | Siege Ram can quickly destroy Cannon Towers, but a cannonball from a Cannon Tower can also destroy a slow-moving Siege Ram with one hit before it gets close. |
| **Scout Cavalry** (Melee) | Scout Cavalry can barely damage Throw Axemen; Throw Axemen can quickly destroy Scout Cavalry. | Scout Cavalry can hardly damage Arrow Towers in a head-on attack and will be quickly eliminated, but they can dodge the ranged arrows with their fast movement speed. | Scout Cavalry can hardly damage Cannon Towers in a head-on attack and will be eliminated in one hit, but they can dodge single cannonballs with their fast movement speed. |
| **Heavy Infantry** (Melee) | Heavy Infantry can quickly eliminate Throw Axemen in melee combat, but Throw Axemen can use their range advantage to eliminate Heavy Infantry first. | Heavy Infantry can barely damage Arrow Towers; Arrow Towers can quickly eliminate slow-moving Heavy Infantry. | Heavy Infantry can barely damage Cannon Towers; Cannon Towers can eliminate slow-moving Heavy Infantry with one hit. |
| **Mounted Archer** (Long-range single arrow) | Horse Archers are suppressed when fighting Throw Axemen at close range, but can use their range and speed advantage to eliminate Throw Axemen from outside the Throw Axemen's range. | Horse Archers can hardly damage Arrow Towers; Arrow Towers can quickly eliminate Horse Archers. | Horse Archers can hardly damage Cannon Towers in a head-on attack and will be eliminated in one hit, but they can dodge single cannonballs with their fast movement speed. |

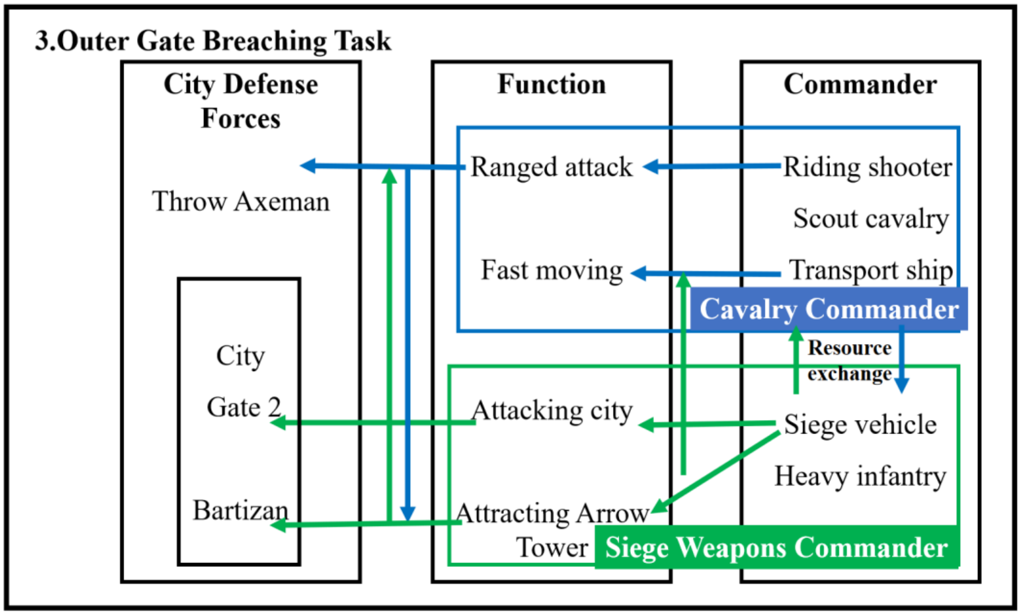


Figure B9. Task decomposition of the Outer Gate Breaching Task.

Starting from the Outer Gate Breaching Task, the difficulty of the tasks and the demands on collaborative skills gradually increase. The level of collaboration and the time limit become increasingly important for successfully completing the task. The main purpose of designing the Outer Gate Breaching Task, the Turret Destruction Task, and the Town Center Capture Task is to focus on individuals' behavioral performance in difficult tasks that can only be completed through collaboration. For the needs of CPS competency assessment, several factors affecting task completion efficiency are designed in the Outer Gate Breaching Task, of which the three most important are:

First, the allocation ratio of the 5 villagers when gathering resources and resource exchange. Upon entering the siege stage, collaborators can train hidden units, which consume resources that are only useful to the partner and are otherwise useless to the commander who gathers them. For the partner, these useless resources might not be gathered at all or only by a few villagers, creating a "short supply" problem that prevents the training of hidden units due to a lack of resources, thus reducing efficiency. Collaborators need to adjust the allocation of their villagers in a timely manner, dispatch more villagers to gather the resources needed by their partner, and provide them promptly to ensure the smooth training of hidden units.

Second, understanding the enemy's attack patterns. The siege stage presents two attack directions: Gate 1 and Gate 2. The defensive power at Gate 2 is significantly weaker than at Gate 1, so choosing Gate 2 as the main attack direction is clearly more efficient. The defenses at Gate 2 consist of Throw Axemen, the city gate, and Arrow Towers, from outside to inside. Because the Arrow Towers have a significantly longer range than the Throw Axemen, they will attack the allied units approaching the moat first. The Throw Axemen will only start attacking once the alliance moves near them. Therefore, to breach the defenses at Gate 2, the alliance must be able to eliminate the Throw Axemen while ensuring they themselves are not destroyed by the Arrow Towers during the attack. This requires a deep understanding of the enemy's attack patterns to formulate a targeted siege plan. Furthermore, because Gate 1 is defended by six devastating Cannon Towers and is nearly impossible to breach head-on, choosing Gate 1 as the main attack direction is very inefficient.

Third, unit combinations. After obtaining the hidden units, each collaborator can choose from two types of units, giving the alliance at least 9 different unit combinations. In this context, choosing the correct unit combination becomes a key factor affecting problem-solving efficiency. For the siege stage, the Mounted Archer, which excels at ranged attacks and has fast movement speed, can replace the Scout Cavalry. However, the Heavy Infantry's melee advantage cannot replace the Siege Ram's role in siege warfare. Therefore, the combination of Horse Archers and Battering Rams is the optimal solution for the siege stage. Blindly pursuing hidden units while ignoring their characteristics will reduce collaborative efficiency.

From the perspective of completing the final task, the theoretical optimal solution for the Outer Gate Breaching Task is as follows:

（1）Explain the characteristics and resource requirements of each other's hidden units to the partner. Gather and provide the necessary resources. Use Scout Cavalry or Horse Archers to scout the enemy situation and firepower at Gate 1 and Gate 2, determining that both gates have strong defenses, but Gate 2 is easier to breach.

（2）Test the enemy's attack patterns by sending a small force to attack, identifying the enemy's attack priority and range. Through testing, realize that only Horse Archers can eliminate Throw Axemen using their range advantage, and only Battering Rams can draw fire from and destroy Arrow Towers. The Heavy Infantry is of little value in the siege stage. Quickly replenish fallen units and use Transport Ships to rapidly transport units to the front line.

（3）Under this premise, purposefully train 3 Battering Rams (rapidly transported by Transport Ships) and at least 2 Horse Archers. First, have the Battering Rams stop near the river to continuously draw fire from the Arrow Towers. Then, have the Horse Archers use their range advantage to eliminate the Throw Axemen from outside the Throw Axemen's range. Finally, have the Battering Rams attack and destroy the city wall and the 4 Arrow Towers.

From the perspective of smoothly entering the next stage, the theoretical minimum requirements for the Outer Gate Breaching Task are as follows:

（1）Expend a significant amount of time and resources, using trial and error to first eliminate the 5 Throw Axemen with Horse Archers, and then destroy the city gate and 4 Arrow Towers with Battering Rams.

Based on the above text, Table B7 further analyzes the relationship between task design and CPS competency assessment in the Outer Gate Breaching Task, combining it with the theoretical requirements of the CPS competency assessment framework. These analyses will contribute to the subsequent cognitive task analysis and scoring model design.

Table B7. Relationship between Task Design and CPS Competency Assessment in the Outer Gate Breaching Task

| Sub-facet | Element | Relationship with Task Design |
|---|---|---|
| Comprehension and Representation | Task Context | **Low:** Overwhelmed or mechanically apply tutorials from the practice phase.<br>**Medium:** Realize the need to dispatch troops to destroy the defenses at the city gate.<br>**High:** Based on the medium level, realize the need to prioritize attacking Gate 2. |
| | Own Role | **Cavalry Commander:**<br>**Low:** Misunderstand or are unaware of the role of Horse Archers.<br>**Medium:** Believe Horse Archers can replace Scout Cavalry.<br>**High:** Based on the medium level, realize the need for Horse Archers to use their range advantage to suppress the enemy.<br>**Siege Weapon Commander:** |

| | | |
|---|---|---|
| | | **Low:** Misunderstand or are unaware of the role of Heavy Infantry.<br>**Medium:** Believe Heavy Infantry cannot replace the function of Battering Rams.<br>**High:** Based on the medium level, realize the need for Battering Rams to actively draw fire from Arrow Towers. |
| | Peer Role | **Cavalry Commander:**<br>**Low:** Only know the peer can train Heavy Infantry, but lack in-depth understanding.<br>**Medium:** Recognize that Heavy Infantry excel in melee but are slow and unsuitable for siege warfare.<br>**High:** Based on the medium level, give the peer correct action suggestions, such as needing Battering Rams to draw fire from Arrow Towers.<br>**Siege Weapon Commander:**<br>**Low:** Only know the peer can train Horse Archers, but lack in-depth understanding.<br>**Medium:** Recognize that Horse Archers excel at ranged attacks but are not good at siege warfare.<br>**High:** Based on the medium level, give the peer correct action suggestions, such as using range advantage to suppress enemy ranged units. |
| Communication and Exchange | View Expression | Topics are limited to resource exchange, unit training, and siege warfare.<br>**Example:** Hoping to attack Gate 2 together with the peer.<br>**Low:** Do not initiate proposals; only passively agree if the peer proposes first.<br>**Medium:** "Let's attack Gate 2 first."<br>**High:** "Gate 2 has no cannon towers and weaker firepower. Let's attack Gate 2." |
| | Perspective-Taking | Topics are limited to resource exchange, unit training, and siege warfare.<br>**Example:** The peer proposes prioritizing an attack on Gate 2.<br>**Low:** Pretend not to hear or agree directly.<br>**Medium:** "Yes, Gate 2 does look easier to attack."<br>**High:** "Okay, we can first have the Horse Archers clear the Throw Axemen guarding the gate." |
| | View Negotiation | Topics are limited to resource exchange, unit training, and siege warfare.<br>**Example:** The peer proposes prioritizing an attack on Gate 1. |

| | | |
|---|---|---|
| | | **Low:** "No, we attack Gate 2 first."<br>**Medium:** "Fine, whatever you say."<br>**High:** "Let's first test the firepower of both gates, then decide which one to attack, okay?" |
| Planning | Goal Setting | **Low:** Set no goal or only aim to complete the task quickly.<br>**Medium:** Set the goal to destroy all enemy city defenses.<br>**High:** Set the goal to find and clear a path into the enemy town's interior. |
| | Problem Deconstruction | **Low:** Restate or emphasize one's own problems (e.g., how to attack, what to do if we can't win).<br>**Medium:** Able to deconstruct the problem as "destroy city defenses," but ignore efficiency (e.g., unit combinations and attack order of the alliance).<br>**High:** Able to deconstruct the problem as "enemy analysis - destroy city defenses," and pay attention to efficiency (see theoretical optimal solution). |
| | Resource Management | **Low:** Only consider one's own resources when planning.<br>**Medium:** Propose joint action when planning but fail to provide a coordination plan that leverages both sides' strengths (see theoretical optimal solution).<br>**High:** Propose joint action when planning and provide a coordination plan that leverages both sides' strengths (see theoretical optimal solution). |
| Execution | Task Performance | **Low:** No plan formulated, or actual actions during execution do not match the plan.<br>**Medium:** Can generally follow the plan, but errors or omissions occur during execution.<br>**High:** Can follow the plan, and no errors or omissions occur during execution. |
| | Coordination | **Low:** Do not provide or delay providing specific resources to the peer when the peer needs them.<br>**Medium:** Promptly provide specific resources to the peer when the peer needs them.<br>**High:** Can coordinate actions based on the peer's resource requirements for training hidden units (e.g., adjusting the number of villagers gathering specific resources). |
| | Outcome Evaluation | After one's own units are killed by enemy attacks:<br>**Low:** Continue attacking according to the original action pattern.<br>**Medium:** Try to analyze the cause of death and adjust actions, but without success. |

| | | |
|---|---|---|
| | | **High:** Try to analyze the cause of death and adjust actions, successfully destroying the enemy's city defenses. |
| Team Responsibility | Collective Awareness | **Low:** Conduct activities independently without communicating with the peer.<br>**Medium:** Able to report one's own progress to the peer but ignore the peer's activities.<br>**High:** Able to monitor the peer's progress and give feedback based on one's own situation. |
| | Concentration | **Low:** Unable to focus on problem-solving; easily distracted or off-topic.<br>**Medium:** Do not initiate off-topic talks but may be distracted by the peer.<br>**High:** Do not initiate off-topic talks and can timely correct the peer's off-topic behaviors. |
| | Task Advancement | Topics are limited to resource exchange, unit training, and siege warfare.<br>**Example:** Exploring the solution direction.<br>**Low:** Take no action to explore the solution.<br>**Medium:** Analyze or attempt operations alone to explore the solution.<br>**High:** Collaboratively explore the solution through communication with the peer. |
| Peer Relationship | Behavioral Norms | Topics are limited to resource exchange, unit training, and siege warfare.<br>**Low:** Interrupt, tease, or order the peer's speech or actions.<br>**Medium:** Respect the peer's contributions, speech, and actions.<br>**High:** Praise or affirm the peer's key contributions, speech, and actions. |
| | Team Support | Topics are limited to resource exchange, unit training, and siege warfare.<br>**Example:** When the peer loses confidence due to repeated failures.<br>**Low:** "Maybe we should just give up."<br>**Medium:** "It's okay, we've already found the problem. Let's try a bit harder and we'll succeed."<br>**High:** (Proactively notices the problem) "We're already halfway there. I believe we'll make a breakthrough soon. Hang in there!" |

**Design of Task 4: Turret Destruction Task**

After breaking through the city gate defense, the allied forces can enter the city. They can scout and discover that the enemy's Town Center is located on the far right of the city. However, to advance further, they will be attacked by a Turret as shown in Figure B11. During the Breaching Phase, the Turret is located on the only path to the Town Center (other paths are blocked). Therefore, the allies must collaborate to launch a breaching operation, completely destroying this Turret to safely continue advancing.

Figure B12 further deconstructs the problem of "how to destroy the Turret" in this phase. As discussed in the study's section on trigger mechanisms, although the Turret has extremely high attack power, its attack interval and projectile speed are slow and predictable. This allows fast-moving units (such as Scout Cavalry or Horse Archers) to use their speed advantage to continuously attract and dodge the cannonballs, thus creating an opportunity for Battering Rams to destroy the Turret.

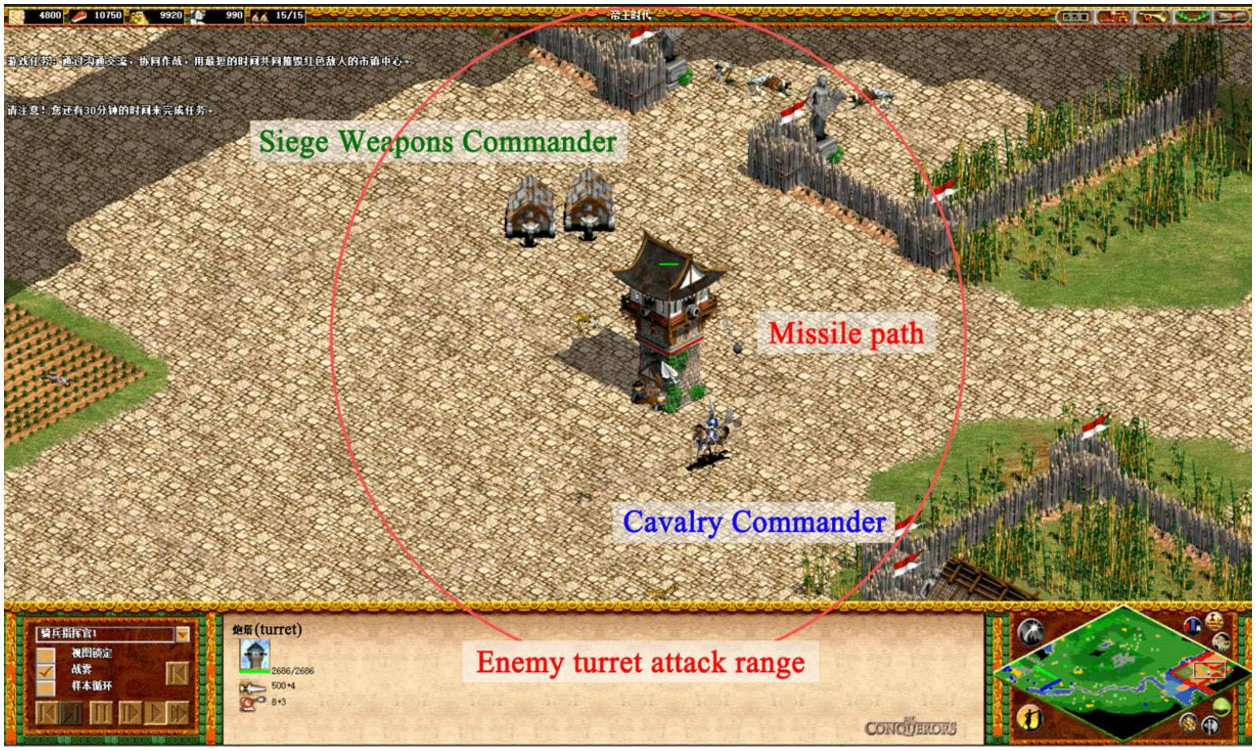


Figure B11. The Inner-City Turret and its Attack Range during the Breaching Phase

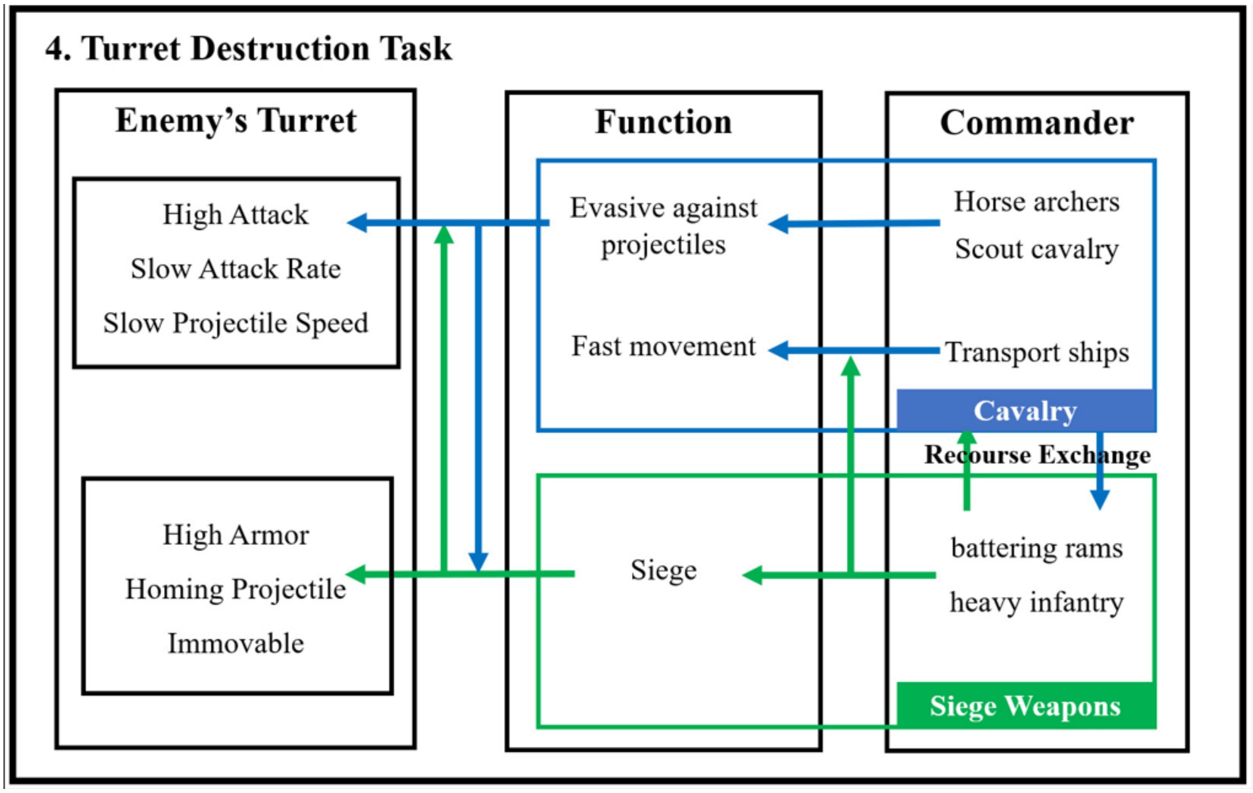


Figure B12. The Task Decomposition of the Breaching Phase

For the needs of CPS competency assessment, several factors affecting task completion efficiency were designed in the Breaching Phase. The three most important factors are similar to those in the previous phases:

First, the analysis of the Turret's attack mode. The collaborators need to analyze the enemy's attack and ballistic characteristics through observation and exploratory attacks: (1) The Turret will continuously fire cannonballs, dealing splash damage, at the first allied unit to enter its range until that unit is destroyed or leaves the range. (2) The Turret's cannonballs are deadly to allied units in one hit, but its attack interval is long, the projectile speed is slow, and the trajectory is easily predictable. Obviously, the Turret combines high attack and high defense, while also causing splash damage in the area near the cannonball's impact point. Therefore, if the allies fail to fully understand the Turret's attack mode and attempt a frontal assault, they will be immediately wiped out by its firepower. In other words, whether the allies can discover the Turret's weakness and adjust their attack strategy accordingly is crucial to completing the task.

Second, the method for determining the Turret's attack mode. In the task design, the allies can only determine the Turret's attack mode through observation. To observe the Turret's attack process, they must first

send units into its attack range. Since the Turret is deadly in one hit, and rebuilding units takes significant time to transport to the front line, the ability to trigger and observe the Turret's attack with minimal unit loss greatly affects task completion efficiency. Under ideal conditions, the most efficient method is for the Cavalry Commander to send a single Scout Cavalry or Mounted Archer to trigger the Turret's attack, allowing both collaborators to observe simultaneously. The least efficient methods are for the collaborators to test and observe independently, or to only use Battering Rams for the test.

Third, consistency of action steps. Destroying the Turret requires two conditions to be met simultaneously. First, the Cavalry Commander must use the speed advantage of Horse Archers or Scout Cavalry to attract and dodge the cannonballs. Second, the Siege Weapon Commander must command Battering Rams to destroy the Turret. Both conditions are indispensable. During this process, the action steps of the two collaborators need to be highly consistent. A mistake by either party could lead to mission failure.

**From the perspective of completing the final task, the theoretical optimal solution for the Breaching Phase is as follows:**

(1) Both collaborators can use a single Scout Cavalry or Mounted Archer for probing, observing and analyzing the Turret's attack mode. They discover that the Turret's weakness lies in its predictable attack interval and projectile trajectory.

(2) One Scout Cavalry or Mounted Archer first enters the Turret's range, using its speed advantage to attract and dodge the Turret's attacks. Then, three siege vehicles disperse close to and destroy the turret.

**From the perspective of smoothly entering the next phase, the theoretical minimum requirements for the Breaching Phase are as follows:**

(1) Consume a large amount of time and resources, fighting a war of attrition by piecemeal encirclement. Deploy units from 6 different directions and then attack simultaneously. Of course, due to the slow movement speed of Battering Rams, even if this method barely allows the Battering Rams to approach the Turret and attack, it will only cause limited damage. It often requires dozens of attempts to succeed, making it very difficult to meet the time limit.

Based on the above text, Table B8 further analyzes the relationship between task design and CPS competency assessment in the Breaching Phase, combining it with the theoretical requirements of the CPS

competency assessment framework. These analyses will contribute to the subsequent cognitive task analysis and scoring model design.

Table B8. Relationship between Task Design and CPS Competency Assessment in the Breaching Phase

| Sub-facet | Element | Relationship with Task Design |
|---|---|---|
| Comprehension and Representation | Task Context | **Low:** Be overwhelmed or mechanically apply the tutorials from the practice phase.<br>**Medium:** Recognize the need to destroy the Turret and other city buildings.<br>**High:** Recognize that only the Turret's defense needs to be breached to attack the Town Center. |
| | Own Role | **Cavalry Commander:**<br>**Low:** Misunderstand or be unaware of the role of Horse Archers or Scout Cavalry.<br>**Medium:** Understand the role of Scout Cavalry or Horse Archers as attracting and dodging cannonballs.<br>**High:** Based on the medium level, use a single unit to probe the Turret's attack mode.<br>**Siege Weapon Commander:**<br>**Low:** Misunderstand or be unaware of the role of Heavy Infantry.<br>**Medium:** Recognize the need to only use Battering Rams to destroy the Turret.<br>**High:** Based on the medium level, command Battering Rams to attack the Turret simultaneously from three directions. |
| | Peer Role | **Cavalry Commander:**<br>**Low:** Only know that the peer can train Battering Rams and Heavy Infantry, but lack in-depth understanding.<br>**Medium:** Recognize that Battering Rams are needed to attack the Turret, but they are too slow to get close.<br>**High:** Based on the medium level, provide correct action suggestions to the peer, such as attacking simultaneously from different directions.<br>**Siege Weapon Commander:**<br>**Low:** Only know that the peer can train Scout Cavalry and Horse Archers, but lack in-depth understanding.<br>**Medium:** Recognize that cavalry can probe the enemy, but their attack is low and they cannot destroy the Turret.<br>**High:** Based on the medium level, provide correct action suggestions to the peer, such as using speed advantage to dodge cannonballs. |

| | | |
|---|---|---|
| Communication and Exchange | View Expression | Topics are limited to resource exchange, unit training, and attacking the Turret.<br>**Example:** Hoping to probe the Turret's attack mode together with the peer.<br>**Low:** Do not initiate proposals; only passively agree if the peer proposes first.<br>**Medium:** "You go up and try attacking the Turret first."<br>**High:** "Cavalry is fast. Let the cavalry go first to probe the Turret's firepower." |
| | Perspective-Taking | Topics are limited to resource exchange, unit training, and attacking the Turret.<br>**Example:** The peer proposes directly attacking the Turret with Battering Rams.<br>**Low:** Pretend not to hear or agree directly.<br>**Medium:** "That's not good. We don't know how strong the Turret is yet. A direct attack will cause losses."<br>**High:** "It's best to let the cavalry probe the firepower first, then send in the Battering Rams." |
| | View Negotiation | Topics are limited to resource exchange, unit training, and attacking the Turret.<br>**Example:** The peer proposes destroying all buildings in the city.<br>**Low:** "No, you do it yourself if you want."<br>**Medium:** "Alright, whatever you say."<br>**High:** "We don't need to attack irrelevant buildings. Just find out where the Town Center is." |
| Planning | Goal Setting | **Low:** Set no goal or only aim to complete the task quickly.<br>**Medium:** Set the goal to destroy the Turret and other city buildings.<br>**High:** Set the goal to find the Town Center and destroy the Turret. |
| | Problem Deconstruction | **Low:** Restate or emphasize one's own problems (e.g., how to attack, what to do if can't win).<br>**Medium:** Able to deconstruct the problem as "destroy the Turret," but ignore efficiency (e.g., unit mix and attack order of the allies).<br>**High:** Able to deconstruct the problem as "find the Town Center – destroy the Turret," and pay attention to efficiency (see theoretical optimal solution). |
| | Resource Management | **Low:** Only consider one's own resources when planning. |

| | | |
|---|---|---|
| | | **Medium:** Propose joint action when planning but fail to provide a coordination plan that leverages both sides' strengths (see theoretical optimal solution).<br>**High:** Propose joint action when planning and provide a coordination plan that leverages both sides' strengths (see theoretical optimal solution). |
| Execution | Task Performance | **Low:** No plan formulated, or actual actions during execution do not match the plan.<br>**Medium:** Can generally follow the plan, but errors or omissions occur during execution.<br>**High:** Can follow the plan, and no errors or omissions occur during execution. |
| | Coordination | **Low:** Need the peer to accommodate one's own actions when attacking the Turret.<br>**Medium:** Can coordinate actions with the peer according to the plan when attacking the Turret.<br>**High:** Can adjust one's own actions based on the peer's actual performance. |
| | Outcome Evaluation | **After one's own units are killed by enemy attacks:**<br>**Low:** Continue attacking according to the original action pattern.<br>**Medium:** Try to analyze the cause of death and adjust actions, but without success.<br>**High:** Try to analyze the cause of death and adjust actions, successfully destroying the enemy turret. |
| Team Responsibility | Collective Awareness | **Low:** Conduct activities independently without communicating with the peer.<br>**Medium:** Able to report one's own progress to the peer but ignore the peer's activities.<br>**High:** Able to monitor the peer's progress and give feedback based on one's own situation. |
| | Concentration | **Low:** Unable to focus on problem-solving; easily distracted or off-topic.<br>**Medium:** Do not initiate off-topic talks but may be distracted by the peer.<br>**High:** Do not initiate off-topic talks and can timely correct the peer's off-topic behaviors. |
| | Task Advancement | Topics are limited to resource exchange, unit training, and attacking the Turret.<br>**Example:** Exploring the solution direction.<br>**Low:** Take no action to explore the solution. |

| | | |
|---|---|---|
| | | **Medium:** Analyze or attempt operations alone to explore the solution.<br>**High:** Collaboratively explore the solution through communication with the peer. |
| Peer Relationship | Behavioral Norms | Topics are limited to resource exchange, unit training, and attacking the Turret.<br>**Low:** Interrupt, tease, or order the peer's speech or actions.<br>**Medium:** Respect the peer's contributions, speech, and actions.<br>**High:** Praise or affirm the peer's key contributions, speech, and actions. |
| | Team Support | Topics are limited to resource exchange, unit training, and attacking the Turret.<br>**Example:** When the peer is transporting units to the front line.<br>**Low:** Do not provide help with Transport Ships.<br>**Medium:** Send Transport Ships to help the peer move troops when requested.<br>**High:** Proactively send Transport Ships in advance to help the peer move troops. |

**Design of Task 5: Town Center Capture Task**

After destroying the turrets inside the city, continue advancing along the road, and you will see the enemy's Town Center, entering the final phase. In front of the Town Center, the last 10 enemy troops are putting up a desperate resistance. As shown in Figure B13, the enemy consists of 5 Sword Shield Infantry (left) and 5 Axe Throwers (right). The allied forces only need to eliminate the remaining enemies and destroy the Town Center to win.

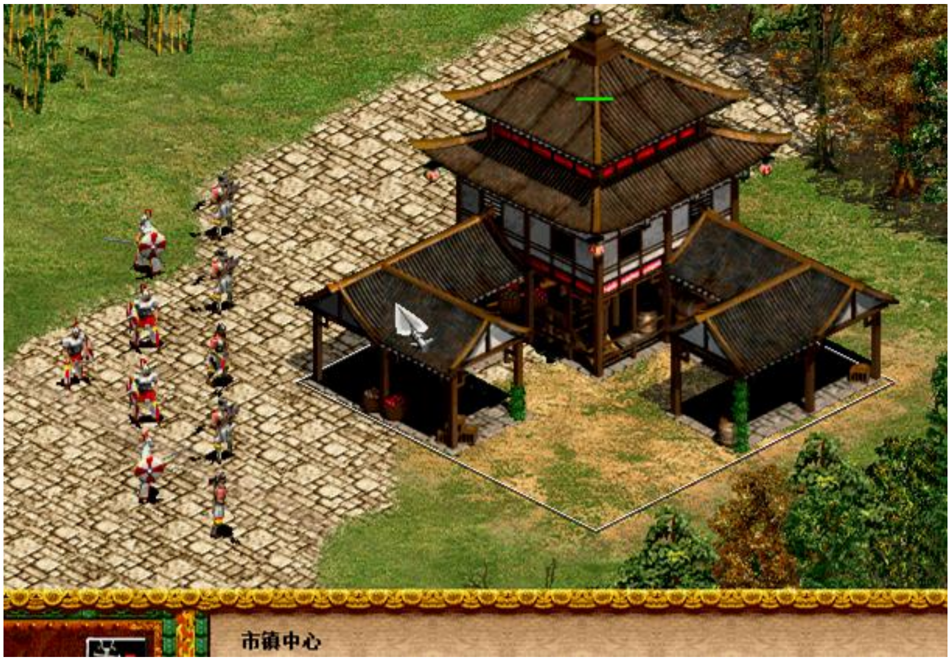


Figure B1. Location of the Town Center and distribution of enemy defenders

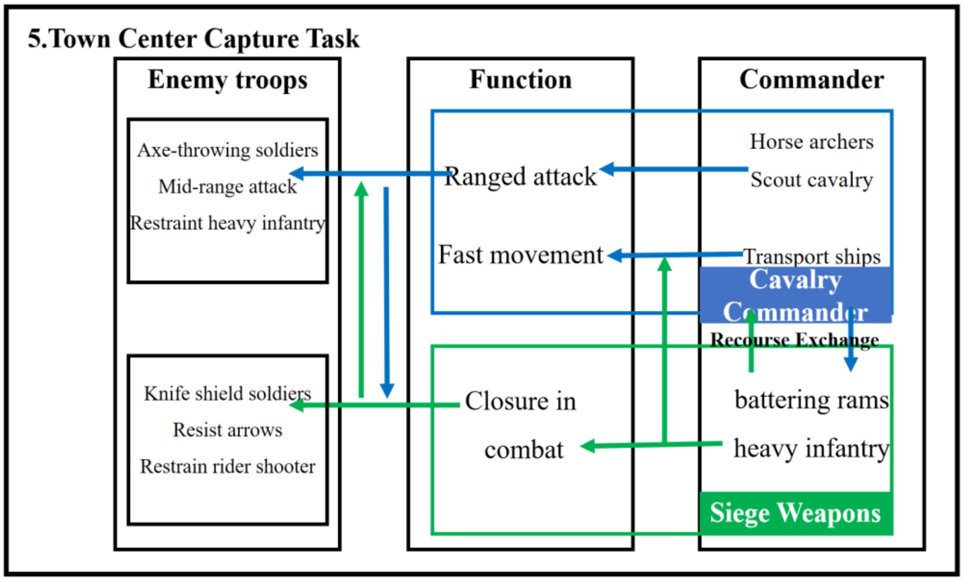


Figure B14. Problem decomposition of the final phase

The Sword Shield Infantry is a light and fast infantry unit. Although its melee combat capability is weak and cannot counterbalance heavy infantry, it is equipped with long swords and shields, making it particularly good at resisting and countering the ranged attacks of Horse Archers. Behind the Sword Shield Infantry, Axe Throwers are deployed. The combination of the two can both eliminate Heavy Infantry that threaten the Sword Shield Infantry and effectively resist the ranged suppression of Horse Archers. Figure B14 further decomposes the problem of "how to destroy the Town Center" in the final phase. As shown, the units of the allied forces and the enemy are mutually countering. Only by adopting the correct attack strategy can victory be achieved. To eliminate the Sword Shield Infantry, the allied forces need to bring back the "forgotten" hidden unit, the Heavy Infantry, which was shelved during the siege and breaching phases. Of course, since Heavy Infantry will be attacked by Axe Throwers before getting close to the Sword Shield Infantry, it is necessary to first sacrifice some Horse Archers to forcibly eliminate the Axe Throwers, and then have the Heavy Infantry attack to eliminate the Sword Shield Infantry.

For the need of CPS capability evaluation, several factors affecting the completion efficiency are designed in the final stage. Similar to the attack stage, the three most important factors are as follows:

First, the analysis of the enemy attack mode. The two sides of the collaboration need to analyze the characteristics of the Knife Shield and the Axe Thrower through experience, observation and tentative attack : (1) The Knife Shield can resist the archery of the archer, and approach and destroy the archer through high-speed movement, only heavy infantry can compete with it. (2) Axemen can use the mid-range attack to eliminate heavy infantry trying to approach, but its range is less than the archer, and cannot resist the arrow. (3) Under the protection of the axe-thrower, the heavy infantry will be eliminated before approaching the shield, but the archer can attack the axe-thrower by virtue of the range advantage. In the final stage, whether the coalition can recognize the characteristics of the enemy 's attack mode determines whether it can successfully complete the task.

Second, the method of determining the enemy attack mode. In the final stage, the coalition can predict the attack mode of the axe by the experience gained in the siege stage, and determine the attack mode of the shield by observation. In order to observe the attack process of the combination of the two, it is necessary to send troops into its attack range. Due to the restraint of arms and the cost of time, the ability to induce and observe enemy attacks with less loss of troops will greatly affect the efficiency of mission completion. Under ideal conditions,

the cavalry commander sends a single riding shooter to induce an enemy attack, and the efficiency of both sides of the collaboration to observe at the same time is the highest; the efficiency of cooperation between the two sides to test and observe independently, or to test only by siege vehicles or heavy infantry is the lowest.

Third, the consistency of action pace. The priority of eliminating knife shield soldiers and axe throwing soldiers is different. The cavalry commander must first use the range advantage of the mounted shooter to forcibly eliminate the axe throwing soldiers, and then the armored commander can dispatch the heavy infantry to eliminate the knife shield soldiers. The inconsistency of the attack order or the failure of any party may lead to the failure of the task.

**From the perspective of completing the final task, the theoretical optimal solution of the siege stage is as follows:**

（1）The two sides of the collaboration can use the experience gained during the siege stage to predict the attack mode of the axe tossers, and then use a single unit to conduct a test. It is found that the weakness of the enemy 's guards is that it is difficult for the shield to eliminate all the riders and shooters in the first time due to the distance problem. Therefore, the riders and shooters can use the range advantage to forcibly attack the axe tossers, depriving the enemy of long-range attack capabilities.

（2）Using the advantage of heavy infantry good at melee, the elimination of shield soldiers, and ultimately destroy the town center to win.

**From the perspective of successfully passing this stage, the minimum theoretical requirements for the final stage are as follows:**

Consuming a lot of time and resources, using oiling tactics to eliminate the war of attrition, at the expense of the whole army to destroy the enemy 's 1-2 soldiers each time, until the enemy is completely destroyed, and ultimately destroy the town center to win. Due to time constraints, it is difficult to use this method to complete the task before the end of the countdown.

On the basis of the above, Table B9 further analyzes the relationship between task design and CPS ability evaluation in the final stage in combination with the theoretical requirements of the CPS ability evaluation framework. These analyses will contribute to the subsequent cognitive task analysis and scoring model design.

Table B9. Relationship between Task Design and CPS Competency Assessment in the Final Phase

| Sub-facet | Element | Relationship with Task Design |
|---|---|---|
| Comprehension and Representation | Task Context | **Low:** Overwhelmed or mechanically apply tutorials from the practice phase.<br>**Medium:** Recognize the need to eliminate the defenders and destroy the Town Center.<br>**High:** Based on the medium level, predict that Axe Throwers can perform ranged attacks. |
| | Own Role | **Cavalry Commander:**<br>**Low:** Misunderstand the role of Horse Archer as eliminating Sword Shield Infantry.<br>**Medium:** Understand the role of Horse Archer as eliminating Axe Throwers, but worry about Sword Shield Infantry.<br>**High:** Recognize the need to prioritize eliminating Axe Throwers with Horse Archers at all costs.<br>**Siege Weapon Commander:**<br>**Low:** Misunderstand or fail to realize the role of Heavy Infantry.<br>**Medium:** Recognize the need to use Heavy Infantry to eliminate Sword Shield Infantry, but ignore Axe Throwers.<br>**High:** Recognize that Heavy Infantry should be used to eliminate Sword Shield Infantry only after the Axe Throwers are eliminated. |
| | Peer Role | **Cavalry Commander:**<br>**Low:** Only know that the peer can train Battering Rams and Heavy Infantry, but lack in-depth understanding.<br>**Medium:** Recognize that Heavy Infantry are needed to eliminate Sword Shield Infantry, but they are countered by Axe Throwers.<br>**High:** Based on the medium level, give correct action suggestions to the peer, such as suggesting the peer train Heavy Infantry.<br>**Siege Weapon Commander:**<br>**Low:** Only know that the peer can train Scout Cavalry and Horse Archers, but lack in-depth understanding.<br>**Medium:** Recognize that Horse Archers can eliminate Axe Throwers but are countered by Sword Shield Infantry. |

| | | |
|---|---|---|
| | | **High:** Based on the medium level, give correct action suggestions to the peer, such as eliminating Axe Throwers first at all costs. |
| Communication and Exchange | View Expression | Topics limited to resource exchange, unit training, and eliminating defenders.<br>**Example:** Hoping to attack the defenders together with the peer.<br>**Low:** Do not initiate proposals; only passively agree if the peer proposes first.<br>**Medium:** "Let's attack them together."<br>**High:** "Let's concentrate our forces and defeat them with numerical advantage." |
| | Perspective-Taking | Topics limited to resource exchange, unit training, and eliminating defenders.<br>**Example:** The peer proposes to attack Sword Shield Infantry directly with Heavy Infantry.<br>**Low:** Pretend not to hear or agree directly.<br>**Medium:** "There are Axe Throwers behind the Sword Shield Infantry. Heavy Infantry will die if they go up."<br>**High:** "It's better to eliminate the Axe Throwers first, then send in the Heavy Infantry." |
| | View Negotiation | Topics limited to resource exchange, unit training, and eliminating defenders.<br>**Example:** The peer proposes to eliminate Sword Shield Infantry first.<br>**Low:** "No, you do it yourself if you want."<br>**Medium:** "Fine, whatever you say."<br>**High:** "To kill Sword Shield Infantry you need melee, but Heavy Infantry can't even get close. Impossible." |
| Planning | Goal Setting | **Low:** Set no goal or only aim to complete the task quickly.<br>**Medium:** Set the goal to eliminate Axe Throwers and Sword Shield Infantry.<br>**High:** Set the goal to prioritize eliminating Axe Throwers, then Sword Shield Infantry, and then destroy the Town Center. |
| | Problem Deconstruction | **Low:** Restate or emphasize one's own problems (e.g., how to fight, can't win).<br>**Medium:** Able to deconstruct the problem as "eliminate defenders," but ignore efficiency (e.g., unit mix and attack order). |

| | | |
|---|---|---|
| | | **High:** Able to deconstruct the problem as "eliminate defenders – destroy Town Center" and pay attention to efficiency (see theoretical optimal solution). |
| | Resource Management | **Low:** Only consider one's own resources when planning.<br>**Medium:** Propose joint action when planning but fail to provide a coordination plan that leverages both sides' strengths (see theoretical optimal solution).<br>**High:** Propose joint action when planning and provide a coordination plan that leverages both sides' strengths (see theoretical optimal solution). |
| Execution | Task Performance | **Low:** No plan formulated, or actual actions during execution do not match the plan.<br>**Medium:** Can generally follow the plan, but errors or omissions occur during execution.<br>**High:** Can follow the plan, and no errors or omissions occur during execution. |
| | Coordination | **Low:** Require the peer to accommodate their own actions when attacking the turret.<br>**Medium:** Can coordinate actions with the peer according to the plan when attacking the turret.<br>**High:** Can adjust their own actions based on the peer's actual performance. |
| | Outcome Evaluation | After one's own units are killed by enemy attacks:<br>**Low:** Continue attacking according to the original action pattern.<br>**Medium:** Try to analyze the cause of death and adjust actions, but without success.<br>**High:** Try to analyze the cause of death and adjust actions, successfully destroying the enemy's defensive forces. |
| Team Responsibility | Collective Awareness | **Low:** Conduct activities independently without communicating with the peer.<br>**Medium:** Able to report one's own progress to the peer but ignore the peer's activities.<br>**High:** Able to monitor the peer's progress and give feedback based on one's own situation. |
| | Concentration | **Low:** Unable to focus on problem-solving; easily distracted or off-topic.<br>**Medium:** Do not initiate off-topic talks but may be distracted by the peer.<br>**High:** Do not initiate off-topic talks and can timely correct the peer's off-topic behaviors. |

| | | |
|---|---|---|
| | Task Advancement | Topics limited to resource exchange, unit training, and eliminating defenders.<br>**Example:** Exploring the solution direction.<br>**Low:** Take no action to explore the solution.<br>**Medium:** Analyze or attempt operations alone to explore the solution.<br>**High:** Collaboratively explore the solution through communication with the peer. |
| Peer Relationship | Behavioral Norms | Topics limited to resource exchange, unit training, and eliminating defenders.<br>**Low:** Interrupt, tease, or order the peer's speech or actions.<br>**Medium:** Respect the peer's contributions, speech, and actions.<br>**High:** Praise or affirm the peer's key contributions, speech, and actions. |
| | Team Support | Topics limited to resource exchange, unit training, and eliminating defenders.<br>**Example:** When the peer feels anxious due to time pressure.<br>**Low:** Do not help alleviate the peer's anxiety.<br>**Medium:** Provide support and help when the peer expresses anxiety.<br>**High:** Notice the peer's anxiety and proactively provide support and help. |

Supplementary Materials C: The Observation Scoring Form for Collaborative Siege Task.

Scoring Methods:

| Item Type | Behavioral Indicators | Observation | Scoring Method |
|---|---|---|---|
| Multiple-select Item | A. Decomposes the problem into "pathfinding – destroying external buildings"<br>B. Only chooses to destroy enemy buildings near the river | ☑<br>☐ | Award 2 points for full selection, 1 point for single selection, and 0 points for other cases. Score in this example: 1. |
| Frequency-count Item | A. Passively responds or expresses views ambiguously<br>B. Expresses views coherently and clearly without explaining the rationale to peers<br>C. Clearly states views and rationale to peers using explanatory language | A:3<br>B:4<br>C:2 | Calculate the weighted mean of frequencies for indicators A, B, and C, then add 1 and round to integer. Weights: − 1, 0, 1 respectively. Theoretical scores: 0, 1, 2. Example calculation: round[((−1 × 3)+(0 × 4)+(1×2))/(3+4+2)+1] = 1 point. |
| Single-select Item | A. Provides no support when peers ask for help<br>B. Encounters no difficulty or provides support when peers ask for help<br>C. Detects peers' difficulty and proactively offers support | A | Score 0 for A; 1 for B; 2 for C.<br>Score in this example: 0. |

**The Observation Scoring Form for Resource Preparation and Unit Training Task**

| Element | ItemID | Behavioral Indicators | Observation |
|---|---|---|---|
| Task Context | Task1_Task Context | A. Voluntarily assign villagers to resource collection and form at least one military unit<br>B. Voluntarily recognize that priority should be given to collecting food/timber over gold/stone | ☐<br>☐ |
| Own Role | Task1_Own Role | A. Voluntarily understand that own military units consume specific resources (Scout Cavalry: food; Siege Vehicle: timber)<br>B. Voluntarily recognize the functions of own military units (Scout Cavalry: reconnaissance; Siege Vehicle: siege) | ☐<br>☐ |
| Peer Roles | Task1_Peer Roles | A. Voluntarily ask partner about the characteristics of their military units (Cavalry partner: Siege Vehicle; Armor partner: Scout Cavalry)<br>B. Voluntarily give partner advice on using military units (Scout Cavalry: reconnaissance; Siege Vehicle: siege) | ☐<br>☐ |
| View Expression | Task1_View Expression | A. Respond passively or express views ambiguously<br>B. Express views coherently and clearly without explaining the rationale to partner<br>C. Clearly state own views and rationale to partner with explanatory language | A：<br>B：<br>C： |
| Perspective-Taking | Task1_PerspectiveTaking | A. Ignore or follow others' views without consideration<br>B. Adopt or refute others' views based on evidence<br>C. Incorporate others' opinions into own thinking to propose new views | A：<br>B：<br>C： |
| View Negotiation | Task1_View Negotiation | A. Argue only to emphasize correctness of own view, no reasoning or conflict resolution<br>B. No dispute, or resolve dispute via compromise, middle ground or suspension<br>C. Eliminate dispute through practice or proposing verification schemes | A：<br>B：<br>C： |

| | | |
|---|---|---|
| Goal Setting | Task1_Goal Setting | A. No goal set or goal is to complete task quickly<br>B. Goal is to train troops to find enemy's Town Center<br>C. Goal is to prepare logistics, stockpile resources and recruit more troops |
| Problem Deconstruction | Task1_ProblemDeconstruction | A. Deconstruct problem into "villagers collect resources – consume resources to form troops" □<br>B. Assign at least 4 villagers to collect nearby resources such as timber or food □ |
| Resource Management | Task1_ResourceManagement | A. Plan to use team resources to build Transport Ships □<br>B. Plan to use Transport Ships to carry Siege Vehicles to improve speed □ |
| Task Performance | Task1_Task Performance | A. No plan made or actual actions inconsistent with plan<br>B. Act roughly according to plan but with errors/omissions<br>C. Act strictly according to plan with no errors/omissions |
| Coordination | Task1_Coordination | A. No Transport Ship construction plan or need partner to wait to execute plan<br>B. No delay or procrastination in building Transport Ships<br>C. Proactively provide timber to partner when knowing Transport Ships need timber / proactively approach Siege Vehicles when using ships |
| Outcome Evaluation | Task1_OutcomeEvaluation | A. Keep clicking recruit button when unable to form more troops, ignoring reasons<br>B. Stop clicking recruit button when unable to form more troops but fail to identify correct reason (default if conditions unmet)<br>C. Stop clicking recruit button when unable to form more troops and identify correct reason |
| Collective Awareness | Task1_CollectiveAwareness | A. Act independently without communicating with partner<br>B. Report own progress to partner but ignore partner's activities<br>C. Monitor partner's progress and give feedback based on own situation |
| Concentration | Task1_Concentration | A. Unable to focus on problem-solving, easily distracted or off-topic<br>B. Do not initiate off-topic talk but easily influenced by partner to stray<br>C. Do not initiate off-topic talk and timely correct partner's off-topic behavior |
| Task Advancement | Task1_Task Advancement | A. Take no action to find solution direction<br>B. Analyze independently or attempt operations to find solution direction<br>C. Communicate with partner to jointly find solution direction |
| Behavioral Norms | Task1_BehavioralNorms | A. Interrupt, mock or order others' speech/actions<br>B. Respect others' contributions, speech or actions<br>C. Praise or affirm others' key contributions, speech or actions |
| Team Support | Task1_Team Support | A. Do not support partner when help is requested<br>B. No difficulty encountered or support partner when help is requested<br>C. Notice partner's difficulty and proactively offer support |

**The Observation Scoring Form for Exploration and Path Selection Task**

| Element | ItemID | Behavioral Indicators | Observation |
|---|---|---|---|
| Task Context | Task2_Task Context | A. Voluntarily send troops to explore map and locate enemy base<br>B. Voluntarily prioritize exploration along riverbanks | □<br>□ |
| Own Role | Task2_Own Role | A. Mainly use Scout Cavalry to attack enemy buildings<br>B. Mainly use Scout Cavalry to explore paths; retreat when encountering enemies<br>C. Mainly use Scout Cavalry to explore paths; use numerical/speed advantage to evade attacks and penetrate deeper when encountering enemies | (Cavalry Commander Only) |
| | | A. Mainly use Siege Vehicle to explore paths<br>B. Mainly use Siege Vehicle to destroy enemy buildings but unaware to gather all 3 Siege Vehicles for joint attack<br>C. Mainly use Siege Vehicle to destroy enemy buildings; aware to gather all 3 Siege Vehicles for joint attack | (Siege Weapons Commander Only) |
| Peer Roles | Task2_Peer Roles | A. Only know partner can train Siege Vehicles without in-depth understanding<br>B. Recognize Siege Vehicles excel at siege but are slow, unsuitable for exploration<br>C. Give partner correct advice such as concentrating forces for attack | (Cavalry Commander Only) |
| | | A. Only know partner can train Scout Cavalry without in-depth understanding<br>B. Recognize Scout Cavalry excel at exploration but have low attack, unsuitable for siege<br>C. Give partner correct advice such as using speed to penetrate deep behind enemy lines | (Siege Weapons Commander Only) |
| View Expression | Task2_View Expression | A. Respond passively or express views ambiguously<br>B. Express views coherently and clearly without explaining the rationale to partner<br>C. Clearly state own views and rationale to partner with explanatory language | A：<br>B：<br>C： |
| Perspective-Taking | Task2_PerspectiveTaking | A. Ignore or follow others' views without consideration<br>B. Adopt or refute others' views based on evidence<br>C. Incorporate others' opinions into own thinking to propose new views | A：<br>B：<br>C： |
| View Negotiation | Task2_View Negotiation | A. Argue only to emphasize correctness of own view, no reasoning or conflict resolution<br>B. No dispute, or resolve dispute via compromise, middle ground or suspension<br>C. Eliminate dispute through practice or proposing verification schemes | A：<br>B：<br>C： |
| Goal Setting | Task2_Goal Setting | A. No goal set or goal is to complete task quickly<br>B. Goal is to destroy all enemy buildings outside the city<br>C. Goal is to find and open a path to enemy town | A：<br>B：<br>C： |
| Problem Deconstruction | Task2_ProblemDeconstruction | A. Deconstruct problem into "explore paths – destroy outer-city buildings"<br>B. Only choose to destroy enemy buildings near rivers | □<br>□ |

| | | | |
|---|---|---|---|
| Resource Management | Task2_ResourceManagement | A. Propose joint actions for both sides when planning | □ |
| | | B. Provide coordination plans to leverage both sides' strengths | □ |
| Task Performance | Task2_TaskPerformance | A. No plan made or actual actions inconsistent with plan | |
| | | B. Act roughly according to plan but with errors/omissions | |
| | | C. Act strictly according to plan with no errors/omissions | |
| Coordination | Task2_Coordination | A. Do not use Transport Ships or need Siege Vehicle to approach to board | |
| | | B. Board Siege Vehicles at agreed location | |
| | | C. Proactively approach Siege Vehicle location for partner to board | |
| Outcome Evaluation | Task2_OutcomeEvaluation | A. Continue original attack mode after troops are killed by enemy | |
| | | B. Analyze cause of death and adjust actions after troops are killed but fail | |
| | | C. Analyze cause of death and adjust actions after troops are killed; successfully explore or destroy enemy buildings | |
| Collective Awareness | Task2_CollectiveAwareness | A. Act independently without communicating with partner | |
| | | B. Report own progress to partner but ignore partner's activities | |
| | | C. Monitor partner's progress and give feedback based on own situation | |
| Concentration | Task2_Concentration | A. Unable to focus on problem-solving, easily distracted or off-topic | |
| | | B. Do not initiate off-topic talk but easily influenced by partner to stray | |
| | | C. Do not initiate off-topic talk and timely correct partner's off-topic behavior | |
| Task Advancement | Task2_TaskAdvancement | A. Take no action to find solution direction | |
| | | B. Analyze independently or attempt operations to find solution direction | |
| | | C. Communicate with partner to jointly find solution direction | |
| Behavioral Norms | Task2_BehavioralNorms | A. Interrupt, mock or order others' speech/actions | |
| | | B. Respect others' contributions, speech or actions | |
| | | C. Praise or affirm others' key contributions, speech or actions | |
| Team Support | Task2_TeamSupport | A. Do not support partner when help is requested | |
| | | B. No difficulty encountered or support partner when help is requested | |
| | | C. Notice partner's difficulty and proactively offer support | |

**The Observation Scoring Form for Outer Gate Breaching Task**

| Element | ItemID | Behavioral Indicators | Observation |
|---|---|---|---|
| Task Context | Task3_Task Context | A. Voluntarily send troops to destroy gate defenses<br>B. Prioritize attacking left gate | □<br>□ |
| Own Role | Task3_Own Role | (Cavalry Commander Only)<br>A. Think Cavalry Archers can replace Scout Cavalry<br>B. Think Cavalry Archers should suppress enemies with range advantage | □<br>□ |
| | | (Siege Weapons Commander Only)<br>A. Think Heavy Infantry cannot replace Siege Vehicles<br>B. Think Siege Vehicles should proactively attract tower fire | □<br>□ |
| Peer Roles | Task3_Peer Roles | (Cavalry Commander Only)<br>A. Recognize Heavy Infantry excel at melee but are slow, unsuitable for siege<br>B. Give partner correct advice such as using Siege Vehicles to attract tower fire | □<br>□ |
| | | (Siege Weapons Commander Only)<br>A. Recognize Cavalry Archers excel at ranged attack but unsuitable for siege<br>B. Give partner correct advice such as using range to suppress enemy ranged units | □<br>□ |
| View Expression | Task3_View Expression | A. Respond passively or express views ambiguously<br>B. Express views coherently and clearly without explaining the rationale to partner<br>C. Clearly state own views and rationale to partner with explanatory language | A：<br>B：<br>C： |
| Perspective-Taking | Task3_PerspectiveTaking | A. Ignore or follow others' views without consideration<br>B. Adopt or refute others' views based on evidence<br>C. Incorporate others' opinions into own thinking to propose new views | A：<br>B：<br>C： |
| View Negotiation | Task3_View Negotiation | A. Argue only to emphasize correctness of own view, no reasoning or conflict resolution<br>B. No dispute, or resolve dispute via compromise, middle ground or suspension<br>C. Eliminate dispute through practice or proposing verification schemes | A：<br>B：<br>C： |
| Goal Setting | Task3_Goal Setting | A. No goal set or goal is to complete task quickly<br>B. Goal is to destroy all enemy city defenses<br>C. Goal is to find and open a path into enemy town | A：<br>B：<br>C： |
| Problem Deconstruction | Task3_ProblemDeconstruction | A. Deconstruct problem into "destroy gate defenses"<br>B. Identify range difference and arrow resistance as key to destroying left gate defenses | □<br>□ |
| Resource Management | Task3_ResourceManagement | A. Propose joint actions for both sides when planning<br>B. Provide coordination plans to leverage both sides' strengths | □<br>□ |
| Task Performance | Task3_Task Performance | A. No plan made or actual actions inconsistent with plan<br>B. Act roughly according to plan but with errors/omissions<br>C. Act strictly according to plan with no errors/omissions | |

| | | |
|---|---|---|
| Coordination | Task3_Coordination | A. Do not provide or delay specific resources when partner needs<br>B. Provide specific resources timely when partner needs<br>C. Coordinate actions (e.g., adjust villager count) based on partner's resource needs for hidden units |
| Outcome Evaluation | Task3_OutcomeEvaluation | A. Continue original attack mode after troops are killed by enemy<br>B. Analyze cause of death and adjust actions after troops are killed but fail<br>C. Analyze cause of death and adjust actions after troops are killed; successfully destroy enemy defenses |
| Collective Awareness | Task3_CollectiveAwareness | A. Act independently without communicating with partner<br>B. Report own progress to partner but ignore partner's activities<br>C. Monitor partner's progress and give feedback based on own situation |
| Concentration | Task3_Concentration | A. Unable to focus on problem-solving, easily distracted or off-topic<br>B. Do not initiate off-topic talk but easily influenced by partner to stray<br>C. Do not initiate off-topic talk and timely correct partner's off-topic behavior |
| Task Advancement | Task3_TaskAdvancement | A. Take no action to find solution direction<br>B. Analyze independently or attempt operations to find solution direction<br>C. Communicate with partner to jointly find solution direction |
| Behavioral Norms | Task3_BehavioralNorms | A. Interrupt, mock or order others' speech/actions<br>B. Respect others' contributions, speech or actions<br>C. Praise or affirm others' key contributions, speech or actions |
| Team Support | Task3_TeamSupport | A. Do not support partner when help is requested<br>B. No difficulty encountered or support partner when help is requested<br>C. Notice partner's difficulty and proactively offer support |

**The Observation Scoring Form for Turret Destruction Task**

| Element | ItemID | Behavioral Indicators | Observation |
|---|---|---|---|
| Task Context | Task4_Task Context | A. Voluntarily destroy inner-city buildings including Turrets<br>B. Voluntarily propose only central Turret needs destruction | □<br>□ |
| Own Role | Task4_Own Role | (Cavalry Commander Only)<br>A. Understand Scout Cavalry/Cavalry Archers function to attract and evade cannon fire<br>B. Use single unit to test Turret attack pattern | □<br>□ |
| | | (Siege Weapons Commander Only)<br>A. Recognize only Siege Vehicles needed to destroy Turret<br>B. Order Siege Vehicles to attack Turret from three directions simultaneously | □<br>□ |
| Peer Roles | Task4_Peer Roles | (Cavalry Commander Only)<br>A. Recognize need Siege Vehicles to attack Turret but they are too slow to approach<br>B. Give partner correct advice such as simultaneous attack from different directions | □<br>□ |
| | | (Siege Weapons Commander Only)<br>A. Recognize Cavalry can test enemies but have low attack unable to destroy Turret<br>B. Give partner correct advice such as using speed to evade cannon fire | □<br>□ |
| View Expression | Task4_View Expression | A. Respond passively or express views ambiguously<br>B. Express views coherently and clearly without explaining the rationale to partner<br>C. Clearly state own views and rationale to partner with explanatory language | A：<br>B：<br>C： |
| Perspective-Taking | Task4_PerspectiveTaking | A. Ignore or follow others’ views without consideration<br>B. Adopt or refute others’ views based on evidence<br>C. Incorporate others’ opinions into own thinking to propose new views | A：<br>B：<br>C： |
| View Negotiation | Task4_View Negotiation | A. Argue only to emphasize correctness of own view, no reasoning or conflict resolution<br>B. No dispute, or resolve dispute via compromise, middle ground or suspension<br>C. Eliminate dispute through practice or proposing verification schemes | A：<br>B：<br>C： |
| Goal Setting | Task4_Goal Setting | A. No goal set or goal is to complete task quickly<br>B. Goal is to destroy Turret and other inner-city buildings<br>C. Goal is to find Town Center and destroy Turret | A：<br>B：<br>C： |
| Problem Deconstruction | Task4_ProblemDeconstruction | A. Deconstruct problem into “destroy Turret”<br>B. Discover Turret weaknesses: predictable attack interval and ballistic trajectory | □<br>□ |
| Resource Management | Task4_ResourceManagement | A. Propose joint actions for both sides when planning<br>B. Provide coordination plans to leverage both sides’ strengths | □<br>□ |

| | | |
|---|---|---|
| Task Performance | Task4_Task Performance | A. No plan made or actual actions inconsistent with plan<br>B. Act roughly according to plan but with errors/omissions<br>C. Act strictly according to plan with no errors/omissions |
| Coordination | Task4_Coor dination | A. Need partner to accommodate self when attacking Turret<br>B. Coordinate with partner per plan when attacking Turret<br>C. Adjust own actions based on partner's actual performance |
| Outcome Evaluation | Task4_Outc omeEvaluati on | A. Continue original attack mode after troops are killed by enemy<br>B. Analyze cause of death and adjust actions after troops are killed but fail<br>C. Analyze cause of death and adjust actions after troops are killed; successfully destroy enemy defenses |
| Collective Awareness | Task4_Colle ctiveAwaren ess | A. Act independently without communicating with partner<br>B. Report own progress to partner but ignore partner's activities<br>C. Monitor partner's progress and give feedback based on own situation |
| Concentration | Task4_Conc entration | A. Unable to focus on problem-solving, easily distracted or off-topic<br>B. Do not initiate off-topic talk but easily influenced by partner to stray<br>C. Do not initiate off-topic talk and timely correct partner's off-topic behavior |
| Task Advancement | Task4_Task Advanceme nt | A. Take no action to find solution direction<br>B. Analyze independently or attempt operations to find solution direction<br>C. Communicate with partner to jointly find solution direction |
| Behavioral Norms | Task4_Beha vioralNorms | A. Interrupt, mock or order others' speech/actions<br>B. Respect others' contributions, speech or actions<br>C. Praise or affirm others' key contributions, speech or actions |
| Team Support | Task4_Team Support | A. Do not support partner when help is requested<br>B. No difficulty encountered or support partner when help is requested<br>C. Notice partner's difficulty and proactively offer support |

**The Observation Scoring Form for Town Center Capture Task**

| Element | ItemID | Behavioral Indicators | Observation |
|---|---|---|---|
| Task Context | Task5_Task Context | A. Voluntarily eliminate defenders and destroy Town Center | □ |
| | | B. Voluntarily predict Axe Throwers can perform ranged attacks | □ |
| Own Role | Task5_Own Role | (Cavalry Commander Only)<br>A. Understand Cavalry Archers eliminate Axe Throwers but wary of Shield Infantry | □ |
| | | B. Recognize priority to use Cavalry Archers to eliminate Axe Throwers at all costs | □ |
| | | (Siege Weapons Commander Only)<br>A. Recognize need Heavy Infantry to eliminate Shield Infantry but unaware of Axe Throwers | □ |
| | | B. Recognize use Heavy Infantry to eliminate Shield Infantry after Axe Throwers are defeated | □ |
| Peer Roles | Task5_Peer Roles | (Cavalry Commander Only)<br>A. Recognize need Heavy Infantry to eliminate Shield Infantry but vulnerable to Axe Throwers | □ |
| | | B. Give partner correct advice such as training Heavy Infantry | □ |
| | | (Siege Weapons Commander Only)<br>A. Recognize Cavalry Archers eliminate Axe Throwers but vulnerable to Shield Infantry | □ |
| | | B. Give partner correct advice such as eliminate Axe Throwers first at all costs | □ |
| View Expression | Task5_View Expression | A. Respond passively or express views ambiguously | A： |
| | | B. Express views coherently and clearly without explaining the rationale to partner | B： |
| | | C. Clearly state own views and rationale to partner with explanatory language | C： |
| Perspective-Taking | Task5_PerspectiveTaking | A. Ignore or follow others' views without consideration | A： |
| | | B. Adopt or refute others' views based on evidence | B： |
| | | C. Incorporate others' opinions into own thinking to propose new views | C： |
| View Negotiation | Task5_View Negotiation | A. Argue only to emphasize correctness of own view, no reasoning or conflict resolution | A： |
| | | B. No dispute, or resolve dispute via compromise, middle ground or suspension | B： |
| | | C. Eliminate dispute through practice or proposing verification schemes | C： |
| Goal Setting | Task5_Goal Setting | A. No goal set or goal is to complete task quickly | A： |
| | | B. Goal is to destroy Town Center | B： |
| | | C. Goal is to eliminate Axe Throwers and Shield Infantry | C： |
| Problem Deconstruction | Task5_ProblemDeconstruction | A. Deconstruct problem into "eliminate defenders" | □ |
| | | B. Identify Cavalry Archers counter Axe Throwers, Heavy Infantry counter Shield Infantry | □ |
| Resource Management | Task5_ResourceManagement | A. Propose joint actions for both sides when planning | □ |
| | | B. Provide coordination plans to leverage both sides' strengths | □ |

| | | |
|---|---|---|
| Task Performance | Task5_Task Performance | A. No plan made or actual actions inconsistent with plan |
| | | B. Act roughly according to plan but with errors/omissions |
| | | C. Act strictly according to plan with no errors/omissions |
| Coordination | Task5_Coordination | A. Need partner to accommodate self when attacking Turret |
| | | B. Coordinate with partner per plan when attacking Turret |
| | | C. Adjust own actions based on partner's actual performance |
| Outcome Evaluation | Task5_OutcomeEvaluation | A. Continue original attack mode after troops are killed by enemy |
| | | B. Analyze cause of death and adjust actions after troops are killed but fail |
| | | C. Analyze cause of death and adjust actions after troops are killed; successfully destroy enemy defenses |
| Collective Awareness | Task5_CollectiveAwareness | A. Act independently without communicating with partner |
| | | B. Report own progress to partner but ignore partner's activities |
| | | C. Monitor partner's progress and give feedback based on own situation |
| Concentration | Task5_Concentration | A. Unable to focus on problem-solving, easily distracted or off-topic |
| | | B. Do not initiate off-topic talk but easily influenced by partner to stray |
| | | C. Do not initiate off-topic talk and timely correct partner's off-topic behavior |
| Task Advancement | Task5_TaskAdvancement | A. Take no action to find solution direction |
| | | B. Analyze independently or attempt operations to find solution direction |
| | | C. Communicate with partner to jointly find solution direction |
| Behavioral Norms | Task5_BehavioralNorms | A. Interrupt, mock or order others' speech/actions |
| | | B. Respect others' contributions, speech or actions |
| | | C. Praise or affirm others' key contributions, speech or actions |
| Team Support | Task5_Team Support | A. Do not support partner when help is requested |
| | | B. No difficulty encountered or support partner when help is requested |
| | | C. Notice partner's difficulty and proactively offer support |

Supplementary Materials D: The item parameters of Collaborative Siege Task.

| Sub-facets | Item Parameters | | | | MNSQ | | ZSTD | |
|---|---|---|---|---|---|---|---|---|
| | Item | $r_{xy}$ | $b$ | $d_1$ | Infit | Outfit | Infit | Outfit |
| Comprehension and Representation | Task1_TaskContext | 0.66 | -0.80 | -0.35 | 0.82 | 0.75 | -2.16 | -2.22 |
| | Task2_TaskContext | 0.76 | 1.01 | -1.57 | 0.72 | 0.71 | -3.65 | -3.83 |
| | Task3_TaskContext | 0.76 | 0.03 | -0.66 | 0.70 | 0.67 | -4.31 | -4.37 |
| | Task4_TaskContext | 0.69 | 0.64 | -0.37 | 0.76 | 0.70 | -3.36 | -3.37 |
| | Task5_TaskContext | 0.53 | -0.27 | -0.16 | 1.10 | 1.15 | 1.19 | 1.38 |
| | Task1_OwnRole | 0.48 | 0.04 | -2.23 | 0.96 | 0.95 | -0.41 | -0.43 |
| | Task2_OwnRole | 0.39 | 0.71 | -2.94 | 1.03 | 1.10 | 0.24 | 0.65 |
| | Task3_OwnRole | 0.55 | 0.78 | -2.04 | 0.99 | 0.99 | -0.06 | -0.09 |
| | Task4_OwnRole | 0.60 | 0.16 | -2.29 | 0.84 | 0.80 | -1.60 | -1.82 |
| | Task5_OwnRole | 0.53 | 0.11 | -2.80 | 0.91 | 0.82 | -0.65 | -1.20 |
| | Task1_PeerRoles | 0.59 | 0.54 | -2.07 | 0.85 | 0.83 | -1.60 | -1.69 |
| | Task2_PeerRoles | 0.54 | 0.61 | -1.89 | 0.96 | 0.96 | -0.46 | -0.41 |
| | Task3_PeerRoles | 0.51 | -0.45 | -2.38 | 0.94 | 0.93 | -0.56 | -0.58 |
| | Task4_PeerRoles | 0.52 | -0.71 | -2.43 | 0.93 | 0.92 | -0.65 | -0.66 |
| | Task5_PeerRoles | 0.27 | 0.00 | -3.50 | 1.07 | 1.15 | 0.41 | 0.69 |
| Communication and Exchange | Task1_ViewExpression | 0.73 | 0.81 | -1.63 | 0.78 | 0.76 | -2.71 | -2.95 |
| | Task2_ViewExpression | 0.68 | -2.09 | -1.15 | 0.78 | 0.72 | -2.19 | -1.90 |
| | Task3_ViewExpression | 0.70 | -0.93 | -1.69 | 0.83 | 0.81 | -2.21 | -2.36 |
| | Task4_ViewExpression | 0.57 | 0.07 | -2.35 | 0.90 | 0.90 | -0.93 | -0.85 |
| | Task5_ViewExpression | 0.82 | 0.12 | -1.22 | 0.58 | 0.57 | -6.07 | -6.12 |
| | Task1_PerspectiveTaking | 0.74 | 1.62 | -1.57 | 0.68 | 0.64 | -4.15 | -4.29 |
| | Task2_PerspectiveTaking | 0.59 | 0.86 | -1.71 | 1.02 | 1.03 | 0.23 | 0.32 |
| | Task3_PerspectiveTaking | 0.68 | 1.11 | -2.59 | 0.71 | 0.63 | -3.02 | -3.31 |
| | Task4_PerspectiveTaking | 0.67 | 0.49 | -1.54 | 0.83 | 0.82 | -2.10 | -2.22 |
| | Task5_PerspectiveTaking | 0.58 | 0.20 | -1.96 | 0.94 | 0.95 | -0.64 | -0.57 |
| | Task1_ViewNegotiation | 0.70 | -0.38 | -0.85 | 0.90 | 0.84 | -1.29 | -1.79 |
| | Task2_ViewNegotiation | 0.76 | -0.96 | -0.96 | 0.66 | 0.65 | -4.43 | -3.74 |
| | Task3_ViewNegotiation | 0.68 | 0.98 | -0.38 | 0.89 | 0.84 | -1.39 | -1.29 |
| | Task4_ViewNegotiation | 0.73 | -0.84 | 0.01 | 0.77 | 0.63 | -2.44 | -2.39 |
| | Task5_ViewNegotiation | 0.69 | -0.04 | -0.25 | 0.92 | 0.87 | -0.88 | -1.16 |
| Planning | Task1_GoalSetting | 0.55 | 0.96 | -1.49 | 0.94 | 0.93 | -0.77 | -0.89 |
| | Task2_GoalSetting | 0.64 | -0.06 | -0.30 | 0.93 | 0.88 | -1.02 | -1.38 |
| | Task3_GoalSetting | 0.65 | 0.23 | -0.94 | 0.89 | 0.89 | -1.40 | -1.50 |
| | Task4_GoalSetting | 0.59 | 0.11 | -1.89 | 0.84 | 0.83 | -1.75 | -1.80 |
| | Task5_GoalSetting | 0.61 | 0.56 | -0.85 | 0.88 | 0.88 | -1.56 | -1.58 |
| | Task1_ProblemDeconstruct | 0.66 | 0.67 | -0.30 | 0.87 | 0.82 | -1.70 | -1.98 |
| | Task2_ProblemDeconstruct | 0.68 | 0.52 | -1.20 | 0.82 | 0.83 | -2.40 | -2.34 |
| | Task3_ProblemDeconstruct | 0.60 | 0.67 | -2.14 | 0.83 | 0.82 | -1.76 | -1.78 |
| | Task4_ProblemDeconstruct | 0.68 | 0.61 | -1.26 | 0.77 | 0.78 | -3.04 | -3.04 |
| | Task5_ProblemDeconstruct | 0.67 | 0.65 | -1.14 | 0.78 | 0.78 | -2.99 | -3.05 |
| | Task1_ResourceManageme | 0.46 | 0.43 | -1.52 | 0.98 | 0.98 | -0.25 | -0.28 |
| | Task2_ResourceManageme | 0.56 | 0.49 | -1.36 | 1.00 | 1.00 | 0.04 | -0.01 |
| | Task3_ResourceManageme | 0.52 | 0.71 | -1.98 | 0.94 | 0.94 | -0.63 | -0.58 |
| | Task4_ResourceManageme | 0.50 | 0.49 | -1.42 | 0.98 | 0.98 | -0.24 | -0.25 |
| | Task5_ResourceManageme | 0.32 | 0.73 | -2.96 | 1.03 | 1.01 | 0.24 | 0.12 |

To be continue,

| Sub-facets | Item Parameters | | | | MNSQ | | ZSTD | |
|---|---|---|---|---|---|---|---|---|
| | Item | $r_{xy}$ | *b* | $d_1$ | Infit | Outfit | Infit | Outfit |
| Execution | Task1_TaskPerformance | 0.20 | 0.05 | -4.59 | 1.24 | 1.18 | 0.72 | 0.54 |
| | Task2_TaskPerformance | 0.40 | 0.57 | -3.23 | 0.97 | 0.96 | -0.11 | -0.14 |
| | Task3_TaskPerformance | 0.41 | 0.90 | -3.29 | 0.78 | 0.67 | -1.41 | -1.85 |
| | Task4_TaskPerformance | 0.43 | 0.30 | -2.52 | 0.91 | 0.88 | -0.72 | -0.85 |
| | Task5_TaskPerformance | 0.27 | 0.02 | -2.93 | 1.10 | 1.15 | 0.70 | 0.94 |
| | Task1_Coordination | 0.62 | 0.20 | -0.53 | 0.88 | 0.86 | -1.63 | -1.83 |
| | Task2_Coordination | 0.66 | -1.05 | -1.23 | 0.74 | 0.71 | -3.47 | -3.78 |
| | Task3_Coordination | 0.57 | 0.00 | -1.04 | 0.99 | 0.99 | -0.06 | -0.15 |
| | Task4_Coordination | 0.63 | 0.79 | -0.43 | 0.88 | 0.82 | -1.65 | -2.03 |
| | Task5_Coordination | 0.52 | -1.11 | -0.77 | 0.95 | 0.89 | -0.55 | -1.06 |
| | Task1_OutcomeEvaluation | 0.53 | 0.09 | -3.17 | 0.68 | 0.52 | -1.97 | -2.94 |
| | Task2_OutcomeEvaluation | 0.62 | -1.43 | -1.31 | 0.81 | 0.78 | -2.35 | -2.61 |
| | Task3_OutcomeEvaluation | 0.61 | -0.11 | -0.74 | 0.92 | 0.90 | -1.07 | -1.29 |
| | Task4_OutcomeEvaluation | 0.61 | -0.08 | -1.91 | 0.83 | 0.82 | -1.81 | -1.91 |
| | Task5_OutcomeEvaluation | 0.57 | -0.86 | -2.48 | 0.85 | 0.84 | -1.60 | -1.44 |
| Team Responsibility | Task1_CollectiveAwareness | 0.63 | -1.11 | -0.73 | 0.80 | 0.74 | -2.36 | -2.42 |
| | Task2_CollectiveAwareness | 0.65 | -0.56 | -1.04 | 0.86 | 0.83 | -1.79 | -2.04 |
| | Task3_CollectiveAwareness | 0.66 | 0.82 | -1.49 | 0.83 | 0.83 | -2.13 | -2.18 |
| | Task4_CollectiveAwareness | 0.57 | 0.02 | -1.76 | 0.89 | 0.89 | -1.18 | -1.20 |
| | Task5_CollectiveAwareness | 0.66 | -0.43 | 0.05 | 0.79 | 0.74 | -2.83 | -2.31 |
| | Task1_Concentration | 0.69 | 0.10 | -0.91 | 0.79 | 0.78 | -2.88 | -2.91 |
| | Task2_Concentration | 0.55 | -0.08 | -0.67 | 1.09 | 1.08 | 1.10 | 0.97 |
| | Task3_Concentration | 0.68 | 0.74 | -1.54 | 0.79 | 0.79 | -2.59 | -2.68 |
| | Task4_Concentration | 0.60 | -0.36 | -0.27 | 0.95 | 0.89 | -0.68 | -1.02 |
| | Task5_Concentration | 0.58 | 0.30 | -0.84 | 0.94 | 0.91 | -0.82 | -1.08 |
| | Task1_TaskAdvancement | 0.66 | -0.39 | -0.27 | 0.82 | 0.75 | -2.42 | -2.62 |
| | Task2_TaskAdvancement | 0.63 | 0.09 | -0.12 | 0.98 | 0.98 | -0.26 | -0.18 |
| | Task3_TaskAdvancement | 0.66 | 0.39 | -0.98 | 0.83 | 0.81 | -2.35 | -2.47 |
| | Task4_TaskAdvancement | 0.54 | -1.00 | -1.46 | 0.90 | 0.89 | -1.23 | -1.39 |
| | Task5_TaskAdvancement | 0.56 | 0.07 | -0.46 | 1.01 | 0.98 | 0.14 | -0.20 |
| Peer Relationship | Task1_BehavioralNorms | 0.77 | -0.27 | -1.40 | 0.65 | 0.65 | -4.74 | -4.80 |
| | Task2_BehavioralNorms | 0.62 | -0.20 | -1.22 | 0.88 | 0.88 | -1.52 | -1.60 |
| | Task3_BehavioralNorms | 0.64 | -0.77 | -1.10 | 0.83 | 0.80 | -2.14 | -2.54 |
| | Task4_BehavioralNorms | 0.49 | -1.13 | -0.52 | 1.03 | 0.96 | 0.31 | -0.28 |
| | Task5_BehavioralNorms | 0.61 | -0.82 | -0.88 | 0.84 | 0.83 | -2.01 | -1.98 |
| | Task1_TeamSupport | 0.63 | -0.23 | -1.67 | 0.86 | 0.86 | -1.63 | -1.66 |
| | Task2_TeamSupport | 0.52 | -0.39 | -2.31 | 0.94 | 0.92 | -0.52 | -0.62 |
| | Task3_TeamSupport | 0.49 | -0.01 | -2.06 | 0.93 | 0.92 | -0.65 | -0.69 |
| | Task4_TeamSupport | 0.60 | -0.17 | -2.02 | 0.88 | 0.88 | -1.20 | -1.20 |
| | Task5_TeamSupport | 0.58 | -0.77 | -2.10 | 0.87 | 0.86 | -1.46 | -1.44 |

Note：All correlations $r_{xy}$ have at least significant correlation level at 0.01.

To be continue,

| Element | Item difficulty *b* | | Thresholds $d_1$ | |
|---|---|---|---|---|
| | *μ* | *σ* | *μ* | *σ* |
| Task Context | 0.12 | 0.93 | -0.60 | 0.63 |
| Own Role | 0.35 | 0.29 | -2.40 | 0.33 |
| Peer Roles | -0.02 | 0.67 | -2.34 | 0.76 |
| View Expression | -0.36 | 1.97 | -1.56 | 0.47 |
| Perspective-Taking | 0.83 | 0.59 | -1.83 | 0.41 |
| View Negotiation | -0.21 | 1.05 | -0.48 | 0.34 |
| Goal Setting | 0.35 | 0.36 | -1.05 | 0.71 |
| Problem Decomposition | 0.62 | 0.02 | -1.17 | 0.77 |
| Resource Management | 0.57 | 0.06 | -1.76 | 0.80 |
| Task Performance | 0.37 | 0.31 | -3.06 | 1.15 |
| Coordination | -0.23 | 1.16 | -0.79 | 0.25 |
| Outcome Evaluation | -0.47 | 0.77 | -1.76 | 1.53 |
| Collective Awareness | -0.24 | 0.91 | -0.95 | 0.93 |
| Concentration | 0.14 | 0.35 | -0.81 | 0.45 |
| Task Advancement | -0.18 | 0.58 | -0.65 | 0.60 |
| Behavioral Norms | -0.63 | 0.33 | -1.00 | 0.28 |
| Team Support | -0.31 | 0.20 | -2.01 | 0.14 |
